# Mapping fatal police violence across U.S. metropolitan areas: Overall rates and racial/ethnic inequities, 2013-2017

**DOI:** 10.1371/journal.pone.0229686

**Published:** 2020-06-24

**Authors:** Gabriel L. Schwartz, Jaquelyn L. Jahn

**Affiliations:** Department of Social & Behavioral Sciences, Harvard T.H. Chan School of Public Health, Boston, MA, United States of America; London School of Economics, UNITED KINGDOM

## Abstract

**Background *&* methods:**

Recent social movements have highlighted fatal police violence as an enduring public health problem in the United States. To solve it, the public requires basic information, such as understanding where rates of fatal police violence are particularly high, and for which groups. Existing mapping efforts, though critically important, often use inappropriate statistical methods and can produce misleading, unstable rates when denominators are small. To fill this gap, we use inverse-variance-weighted multilevel models to estimate overall and race-stratified rates of fatal police violence for all Metropolitan Statistical Areas (MSAs) in the U.S. (2013–2017), as well as racial inequities in these rates. We analyzed the most recent, reliable data from Fatal Encounters, a citizen science initiative that aggregates and verifies media reports.

**Results:**

Rates of police-related fatalities varied dramatically, with the deadliest MSAs exhibiting rates nine times those of the least deadly. Overall rates in Southwestern MSAs were highest, with lower rates in the northern Midwest and Northeast. Yet this pattern was reversed for Black-White inequities, with Northeast and Midwest MSAs exhibiting the highest inequities nationwide. Our main results excluded deaths that could be considered accidents (e.g., vehicular collisions), but sensitivity analyses demonstrated that doing so may underestimate the rate of fatal police violence in some MSAs by 60%. Black-White and Latinx-White inequities were slightly underestimated nationally by excluding reportedly ‘accidental’ deaths, but MSA-specific inequities were sometimes severely under- or over-estimated.

**Conclusions:**

Preventing fatal police violence in different areas of the country will likely require unique solutions. Estimates of the severity of these problems (overall rates, racial inequities, specific causes of death) in any given MSA are quite sensitive to which types of deaths are analyzed, and whether race and cause of death are attributed correctly. Monitoring and mapping these rates using appropriate methods is critical for government accountability and successful prevention.

## Introduction

Over the last decade, the Black Lives Matter movement has drawn renewed attention to fatal police violence as an urgent public health and racial justice problem, defined here as fatalities in police custody or involving the police that would not have occurred in the absence of police intervention. No national, publicly-funded data system has accurately tracked the number of people who die during contact with police, but these deaths are public health data and can be counted [1]. Police-related deaths have distinct causes, distributions, and consequences for population health from other forms of violence and currently number in the thousands every year [1,2]. Men, racial/ethnic minorities, young people, and those living in economically disadvantaged areas are particularly at risk, especially those at the intersection of these social stratifications [2]. Recent analysis of independently-validated data collected by the citizen science initiative Fatal Encounters showed police accounted for more than 1 in 12 of all homicides of adult men between 2012 and 2018 [3]. These same data show that 1 in 1000 Black men can expect to die of police violence over the course of their lifetime if present rates hold [2]. During the same time period, police-related fatalities killed more Black men in their 20s than diabetes, flu/pneumonia, chronic respiratory disease, or cerebrovascular disease [4]. Further, emerging evidence argues that police killings have population health impacts beyond the immediate consequences for decedents and their families. Bor and colleagues, for example, used a quasi-experimental design to find that police killings of unarmed Black Americans worsened the mental health of Black people living in the same state for months thereafter, although no similar effect was found among White people or for other types of police killings [5].

Geography matters for these deaths. Although rates of police killings are highest in neighborhoods with the greatest concentration of low-income residents and residents of color, Black people were recently found to be at greatest risk in predominantly White neighborhoods [6]. Indeed, others have shown that income inequality, degree of racial segregation, and racial make-up of police departments all predicted rates of fatalities involving police in distinct ways for different racial/ethnic minority groups [7]. Rates of police-related fatalities vary dramatically across US Census divisions and tend to be higher in more densely populated areas, compared to those that are less urbanized [3].

Although researchers have begun describing crude differences in rates of fatalities involving police across states and cities [8,9], these important analyses have often made comparisons between places without accounting for variability around point estimates (i.e., without calculating confidence intervals [CIs] around the estimates for each place). They may also estimate rates in unintentionally misleading ways: when race-specific denominators are small, and the number of events rare, a difference of one death can yield raw rates that are astronomical in one area and near zero in another area experiencing effectively the same underlying rate. Moreover, state-level analyses do not reflect important within-state variation in rates of fatal police violence between cities or between urban and rural areas, where policing practices are likely to differ. Indeed, our analysis of national data in this paper shows that 90% of fatalities involving police in this country occurred in metropolitan statistical areas (MSAs), which suggests that MSAs are a key geographic unit of analysis to identify areas where policy changes should be made to address fatal police violence. Model-based measurement of rates of fatalities involving police and the magnitude of racial inequities in these deaths across the country allows us to account for the volatility of those rates over time and provide information about how precisely estimated rates for a given place are. Model-based measurement is thus necessary to identify cities where these rates and inequalities are consistently severe or unusually low, a critical step towards targeting interventions and ensuring policy makers and law enforcement agencies can be held accountable to the public for lowering rates of fatal police violence.

We used multilevel models and 2013–2017 data from Fatal Encounters to estimate rates of fatal police violence for every Metropolitan Statistical Area (MSA), as well as MSA-specific proportional differences in these rates by race/ethnicity, comparing Black and Latinx rates to non-Latinx White rates. This inverse-variance-weighted method allowed us to borrow statistical strength from the full national sample in estimating city-level differences and smooth over volatility in our estimates induced by small populations of certain races or ethnicities in a given area along with rare events. We then calculated confidence intervals for each MSA’s estimate, allowing comparisons between MSAs.

## Methods

### Study population

Our police-related fatalities data is from Fatal Encounters, a citizen science initiative that systematically identified cases in a prospective manner using online media reports and public records; case coding is triple-checked by paid researchers who verify information across multiple media reports and public records [10–12]. The resulting data is more comprehensive than official vital statistics files [11] and is preferable to Bureau of Justice Statistics’ (BJS) Arrest-Related Deaths survey and the FBI’s Supplementary Homicide Reports, which underestimated the number of police-related fatalities during these years [13]. Indeed, Fatal Encounters has been endorsed by the BJS [13]. The Fatal Encounters dataset is also a source for the Mapping Police Violence and The Counted datasets [14,15], but differs from the Washington Post’s Fatal Force Database in that does not exclude deaths of people in police custody, fatal shootings by off-duty police officers, or non-firearm deaths [16]. Fatal Encounters also provides latitudes and longitudes for each incident, allowing aggregation to MSAs. In this analysis, we used their data from 2013 to 2017, downloaded May 1, 2019, as data prior to 2012 was collected retrospectively and race/ethnicity data from after 2017 was still being fact-checked (and thus appeared largely missing in the data Fatal Encounters released to the public).

From the complete Fatal Encounters data for these years, we followed Edwards, Lee, and Esposito [17] and excluded 1,670 cases (23.3%) that were reported to be suicides, accidents (including drug overdoses and other medical emergencies, drowning, or falling from a height), or were caused by a vehicular collision. Our analytic sample for calculating overall rates thus included 5494 police-related fatalities: 94.2% from gunshot wounds, 3.4% from tasering, 1.7% from asphyxiation, pepper spraying, or bludgeoning, and 0.7% from other causes. Our analyses of racial inequities in fatal police violence also excluded 547 deaths lacking race/ethnicity data. In sensitivity analyses, we compared estimates using these data to those from models that included all reported causes of death to assess whether MSA-specific rates of fatal police violence meaningfully differ depending on these inclusion criteria.

We geocoded the Fatal Encounters data using ArcGIS version 10.6.1, assigning each police-related fatality to a Metropolitan Statistical Area (MSA) using latitudes and longitudes from Fatal Encounters and MSA shapefiles provided by the 2010 US Census. We chose MSAs as our unit of analysis because (A) of all qualifying fatalities involving police during this period, 6 in 7 occurred within MSAs, and (B) the country’s 382 MSA boundaries are constructed to represent heavily interconnected urban economic zones. MSA boundaries thus reflect the broader geographic areas in which urban Americans live, work, and socialize; police violence is not constrained to the jurisdictional boundaries of police departments in which Americans’ home addresses lie. MSAs may thus better reflect the experience of U.S. urban residents’ daily lives than counties, police departments, or Census tracts would.

To calculate rates, we aggregated data on fatal police violence to the MSA level and merged it with race-specific population data from the Census Bureau’s American Community Survey 5-Year Estimates from 2013–2017. Due to the small absolute numbers of fatalities involving police in most MSAs, we used fatalities data from all these years to calculate an average annual rate and increase the stability of our estimates, as per Feldman and colleagues [1]. We use population denominators to align with, and allow comparisons to, previous demographic work in this area [2,3,18], and because using race-specific crime or arrest counts—themselves shaped by racial bias and segregation—yields estimates of a different and potentially biased contrast than the rather simple ones we answer here: in which metropolitan areas are people most likely to be killed by police, what is the difference in these rates by race, and how does this vary across MSAs? [19,20]

All racial/ethnic groups’ data were included when calculating overall annual incidence rates of fatal police violence, but from our models estimating racial/ethnic inequities in fatal police violence, we report only Black and Latinx rates compared to White rates. This is because the low total race/ethnicity-specific population and total number of deaths for Native American, Middle Eastern, Asian/Pacific Islander, and people of other races/ethnicities means we lacked sufficient statistical power to estimate meaningful MSA-specific rates for these groups, even aggregated into a single racial/ethnic category. Moreover, Fatal Encounters data do not disaggregate Asian/Pacific Islander people by ethnicity or by the country their family lines originally immigrated to the United States from, making their racial/ethnic group extremely heterogenous in terms of socioeconomic status and police violence risk.

### Statistical analysis

We estimated annual incident rates and incident rate ratios (IRR) for police killings in each MSA using multilevel Poisson models with a population offset, assuming normality of the random effects. Importantly, these empirical Bayesian models use inverse-variance-weighting to estimate national rates and ‘borrow strength’ from rates in the rest of the country to stabilize estimation of MSA-specific rates in places where denominators are low (e.g., when calculating an incidence rate ratio comparing Black and White rates of fatal police violence in MSAs with very small Black populations) [21,22]. In effect, this means that places with very low denominators—places where the variance of MSA-specific estimates would be high—are made less influential when estimating national rates, and MSA-specific rates in these places (or ratios of rates comparing one race to another) are ‘shrunk’ towards the overall national rate (or rate ratio) [23].

We estimated two sets of models. The first set estimated the overall annual incident rate for each MSA using random intercepts. These data were organized as years nested within MSAs, with one dataset for the general population and another for each race, in order to calculate more stable annual incidence rates for each subgroup with its corresponding population offset. The second set of models estimated the IRRs of fatal police violence for each MSA, comparing Black, Latinx, and Other Race/Ethnicity incidence rates to White rates. These racial inequity models used data organized as years nested within race nested within MSAS and included random intercepts at the MSA-level, random slopes for each race/ethnicity indicator, and offsets for the MSA-race-specific population. After estimating these models, we used MSA-level random intercepts and slopes to map (A) overall MSA-specific incidence rates and (B) racial inequity incidence rate ratios (IRRs), comparing Black and Latinx rates to the non-Latinx White rate.

Our main results are calculated using the *mepoisson* command in Stata MP v. 15.1, which uses a Laplacian approximation as its integration method (the default) [24,25]. Given that this estimation procedure can produce biased and imprecise estimates, we conducted a sensitivity analysis comparing the incident rates and standard errors we calculate using *mepoisson* to models that use Markov chain Monte Carlo (MCMC) estimation [26], run with MLwiN v. 2.34 [27] via the *runmlwin* call to the program through Stata [28]. First order marginal quasi-likelihood models were run first to provide starting estimates for MCMC estimation [26].

Finally, we formally examined whether the MSA-level rates we estimated exhibited spatial autocorrelation using the statistical software GeoDa [29]. We calculated global Moran’s I values [30] with a 10 nearest neighbors weights matrix, with weights equal to the inverse of the distance between MSA centroids, allowing the meaningful inclusion of MSAs that were geographically isolated. We repeated this calculation using our estimated Black-White IRR and Latinx-White IRR estimates.

## Results

Of the included 5494 fatalities involving police from 2013–2017, 2353 (42.83%) of the decedents were White, 1487 (27.07%) were Black, 939 were Latinx (17.09%), and 168 (3.06%) were other race/ethnicities, while 547 lacked data on race/ethnicity. Nationally, from our first set of models, the annual rate of fatal police violence was 0.39 per 100,000 (95% Confidence Interval [CI] = 0.37,0.42). Yet overall and race-specific rates varied from MSA to MSA: overall rates ranged from 0.13 (CI = 0.03,0.56) fatalities per 100,000 in Buffalo-Cheektowaga-Niagara Falls, NY to 1.17/100,000 (CI = 0.28,4.99) in Anniston-Oxford-Jacksonville, AL, meaning the most lethal MSA exhibited rates nine times those of the least lethal.

Race-specific rates varied less from MSA to MSA for Black people than for their White or Latinx counterparts. While MSA-specific annual rates for Black people ranged from 0.43/100,000 (Detroit-Warren-Dearborn, MI; CI = 0.12,1.59) to 2.10 (Oklahoma City, OK; CI = 0.54,8.08)—a ratio of maximum to minimum of 4.9—MSA-specific rates for White people ranged from 0.08 (New York-Newark-Jersey City, NY-NJ-PA; CI = 0.02,0.27) to 1.10 (Anniston-Oxford-Jacksonville, AL; CI = 0.24,5.02)—a maximum/minimum ratio of nearly 14—and for Latinx people ranged from 0.11 (New York-Newark-Jersey City, NY-NJ-PA; CI = 0.03,0.39) to 1.27 (Pueblo, CO; CI = 0.28,5.88)—a maximum/minimum ratio of more than 12.

Table 1 provides incidence rates of fatal police violence for the 10 MSAs with the highest incidence rates over the study period, overall (including deaths with no race/ethnicity information) and by race. Overall rankings appeared strongly driven by White rates, given their proportion of the population. Of the race-stratified results, some MSAs’ incidence rates ranked high across multiple racial/ethnic groups (e.g. Oklahoma City, OK), whereas others ranked highly for one racial/ethnic group but not others (e.g. Pueblo, CO). The variability in these estimates and their confidence intervals is visualized in S7 Fig (see Supporting information).

**Table 1:** Annual incidence rates of fatalities involving police per 100,000 and 95% CIs for MSAs with the highest incidence rates, 2013–2017.

Rank	Overall	Black	White	Latinx
1	Anniston-Oxford-Jacksonville, AL	Oklahoma City, OK	Anniston-Oxford-Jacksonville, AL	Pueblo, CO
	1.17 (0.28, 4.99)	2.10 (0.54, 8.08)	1.10 (0.24, 5.02)	1.27 (0.28, 5.88)
2	Farmington, NM	San Francisco-Oakland-Hayward, CA	Lake Havasu City-Kingman, AZ	Greeley, CO
	1.01(0.24, 4.31)	1.85 (0.53, 6.47)	0.90 (0.21, 3.90)	0.87 (0.18, 4.11)
3	Bakersfield, CA	St. Louis, MO-IL	Billings, MT	Bakersfield, CA
	1.01 (0.30, 3.37)	1.56 (0.46, 5.28)	0.86 (0.20, 3.80)	0.81 (0.21, 3.11)
4	Billings, MT	Dayton, OH	Yuba City, CA	Tucson, AZ
	1.01 (0.24, 4.18)	1.51 (0.37, 6.13)	0.82 (0.17, 3.83)	0.80 (0.20, 3.16)
5	Pueblo, CO	Reno, NV	Waco, TX	Wichita Falls, TX
	0.95 (0.23, 3.97)	1.47 (0.31, 6.92)	0.77 (0.17, 3.42)	0.79 (0.15, 4.14)
6	Oklahoma City, OK	Tulsa, OK	Bakersfield, CA	Oklahoma City, OK
	0.93 (0.29, 2.95)	1.45 (0.34, 6.17)	0.75 (0.19, 3.06)	0.79 (0.18, 3.46)
7	Albuquerque, NM	Los Angeles-Long Beach-Anaheim, CA	Longview, TX	Santa Fe, NM
	0.92 (0.27, 3.11)	1.38 (0.43, 4.44)	0.71 (0.16, 3.22)	0.78 (0.16, 3.76)
8	Anchorage, AK	Trenton, NJ	Albuquerque, NM	Albuquerque, NM
	0.90 (0.24, 3.39)	1.36 (0.32, 5.82)	0.71 (0.18, 2.85)	0.77 (0.20, 2.98)
9	Tulsa, OK	Tucson, AZ	Tulsa, OK	Phoenix-Mesa-Scottsdale, AZ
	0.88 (0.26, 2.95)	1.34 (0.30, 6.08)	0.71 (0.19, 2.62)	0.76 (0.23, 2.51)
10	Las Cruces, NM	Columbus, OH	Deltona-Daytona Beach-Ormond Beach, FL	Sierra Vista-Douglas, AZ
	0.86 (0.21, 3.52)	1.33 (0.36, 4.91)	0.70 (0.18, 2.71)	0.67 (0.13, 3.42)

On average, there were large racial/ethnic inequities in the rates at which White and Black people were killed during police contact. Across all MSAs, Black people were 3.23 times more likely to be killed compared to White people (95% CI: 2.95, 3.54, p<0.001). Latinx people were 1.05 times more likely, though this IRR was not statically significant (95% CI: 0.94, 1.17, p = 0.40).

MSA-specific incidence rate ratios also varied. Across all 382 MSAs, Black-White IRRs ranged from 1.81 (Atlanta-Sandy Springs-Roswell, GA; CI: 0.50,6.53) to 6.51 (Chicago-Naperville-Elgin, IL-IN-WI MSA; CI: 1.84, 23.09), a maximum/minimum ratio of 3.6. Latinx-White IRRs varied somewhat less, ranging from 0.71 (again, Atlanta-Sandy Springs-Roswell, GA; 0.18,2.77) to 1.50 (Pueblo, CO; CI: 0.37, 6.00), a large though statistically insignificant ratio of 2.1.

Estimates from the random part of our IRR model suggested that the incidence rates for White and non-White people are related, i.e., MSAs’ random intercepts and random slopes for race were negatively correlated (Black r = -0.32; Latinx r = -0.19). Given Black people’s much higher overall rate compared to White people, MSAs with high rates of fatal police violence against White people tended to have smaller disparities between White and Black rates; or, equivalently, MSAs with low rates of fatal police violence against White people tended to exhibit more extreme Black-White inequities. Among Latinx people, lower White rates tended to go along with higher, more severe Latinx-White inequities, while MSAs with higher White rates tended to have more equal rates across Latinx and White people, or even exhibit lower Latinx rates than White rates.

Table 2 provides estimates of the incidence rate ratios (IRR) comparing Black and Latinx incidence rates to White incidence rates in the 10 MSAs with the highest IRRs for each racial/ethnic comparison. First, the IRRs comparing Black to White incidence rates of fatal police violence are markedly high and are nearly all statistically significant. In the Chicago-Naperville-Elgin, IL-IN-WI MSA, for example, the Black incidence rate of fatal police violence was 6.51 times (95% CI 1.84, 23.09) higher than the White incidence rate. Second, the 10 largest IRRs comparing Latinx to White incidence rates were also high, but the lower bounds of their confidence intervals all fall below the null, potentially due to low power (a low absolute number of Latinx people in many MSAs and/or low numbers of Latinx fatalities).

**Table 2:** Annual incident rate ratios and 95% confidence intervals for MSAs with the largest racial inequities in police killings, 2013–2017.

Rank	Black-White Inequities	Latinx-White Inequities	Other-White Inequities
1	Chicago-Naperville-Elgin, IL-IN-WI	Pueblo, CO	Fairbanks, AK
	6.51 (1.84, 23.09)	1.50 (0.37, 6.00)	1.66 (0.29, 9.35)
2	San Francisco-Oakland-Hayward, CA	Greeley, CO	Minneapolis-St. Paul-Bloomington, MN-WI
	5.87 (1.57, 21.90)	1.41 (0.35, 5.69)	1.23 (0.25, 5.98)
3	New York-Newark-Jersey City, NY-NJ-PA	Tucson, AZ	Bismarck, ND
	5.38 (1.54, 18.75)	1.38 (0.36, 5.27)	0.88 (0.15, 5.14)
4	St. Louis, MO-IL	Los Angeles-Long Beach-Anaheim, CA	Anchorage, AK
	5.14 (1.39, 19.05)	1.37 (0.43, 4.39)	0.85 (0.16, 4.45)
5	Columbus, OH	Wichita Falls, TX	Rapid City, SD
	4.83 (1.22, 19.07)	1.32 (0.32, 5.40)	0.80 (0.14, 4.59)
6	Milwaukee-Waukesha-West Allis, WI	Denver-Aurora-Lakewood, CA	Billings, MT
	4.73 (1.14, 19.62)	1.32 (0.36, 4.87)	0.79 (0.13, 4.56)
7	Trenton, NJ	Phoenix-Mesa-Scottsdale, AZ	Redding, CA
	4.69 (1.06, 20.71)	1.30 (0.38, 4.40)	0.78 (0.14, 4.47)
8	Asheville, NC	Sierra Vista-Douglas, AZ	Seattle-Tacoma-Bellevue, WA
	4.48 (0.98, 20.57)	1.25 (0.31, 5.11)	0.77 (0.17, 4.41)
9	Dayton, OH	Santa Fe, NM	Merced, CA
	4.44 (1.06, 18.57)	1.25 (0.31, 5.04)	0.76 (0.13, 4.35)
10	Reno, NV	Chicago-Naperville-Elgin, IL-IN-WI	Akron, OH
	4.33 (0.95, 19.78)	1.24 (0.34, 4.61)	0.75 (0.13, 4.25)

**Fig 1:**
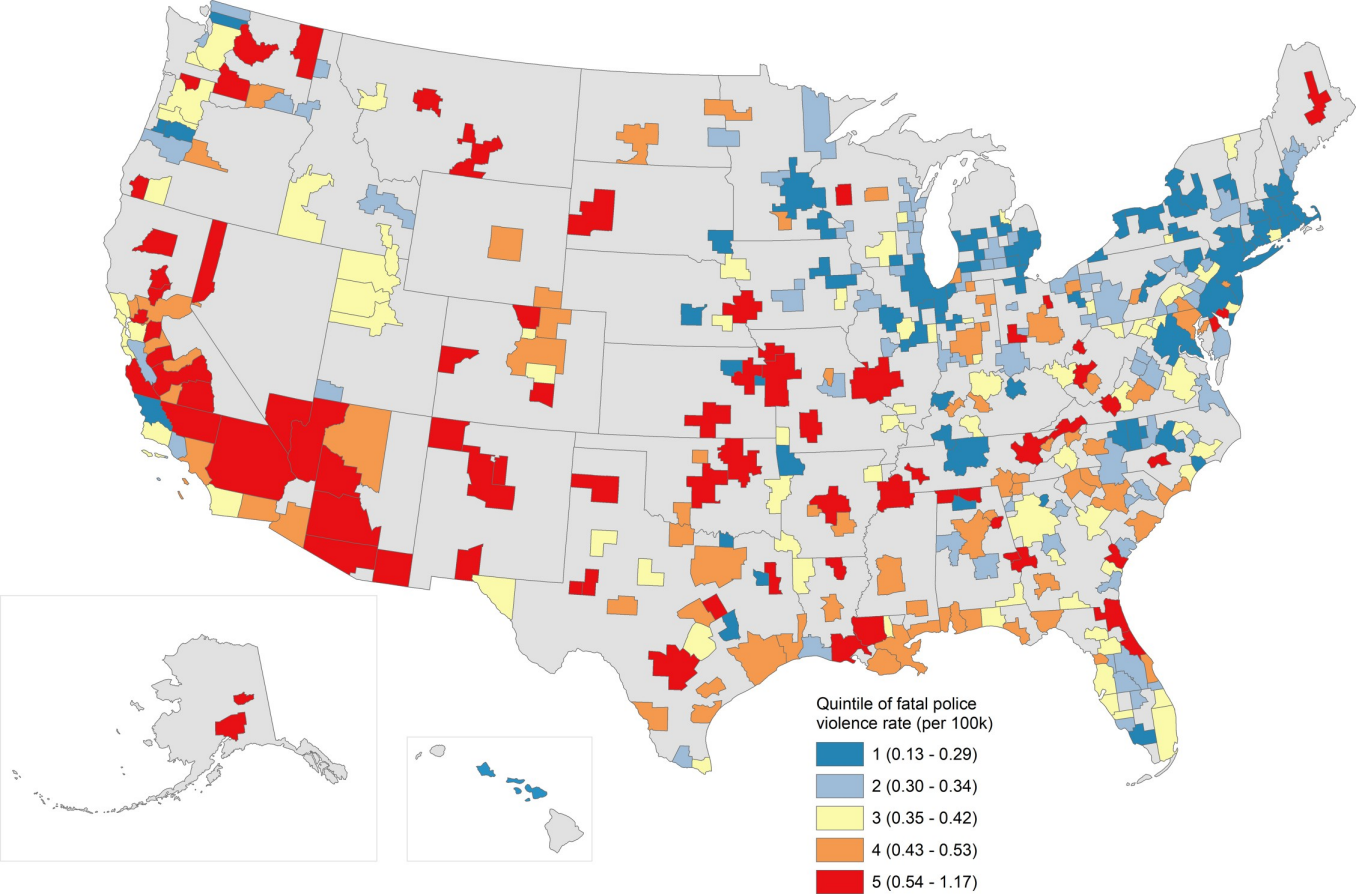
Estimated annual rates of fatal police violence per 100,000 residents, by MSA. Estimated MSA-specific rates of fatal police violence per 100,000 residents per year are mapped. Quintiles are labeled in the legend along with, in parentheses, the range of estimated rates included in that quintile.

**Fig 2:**
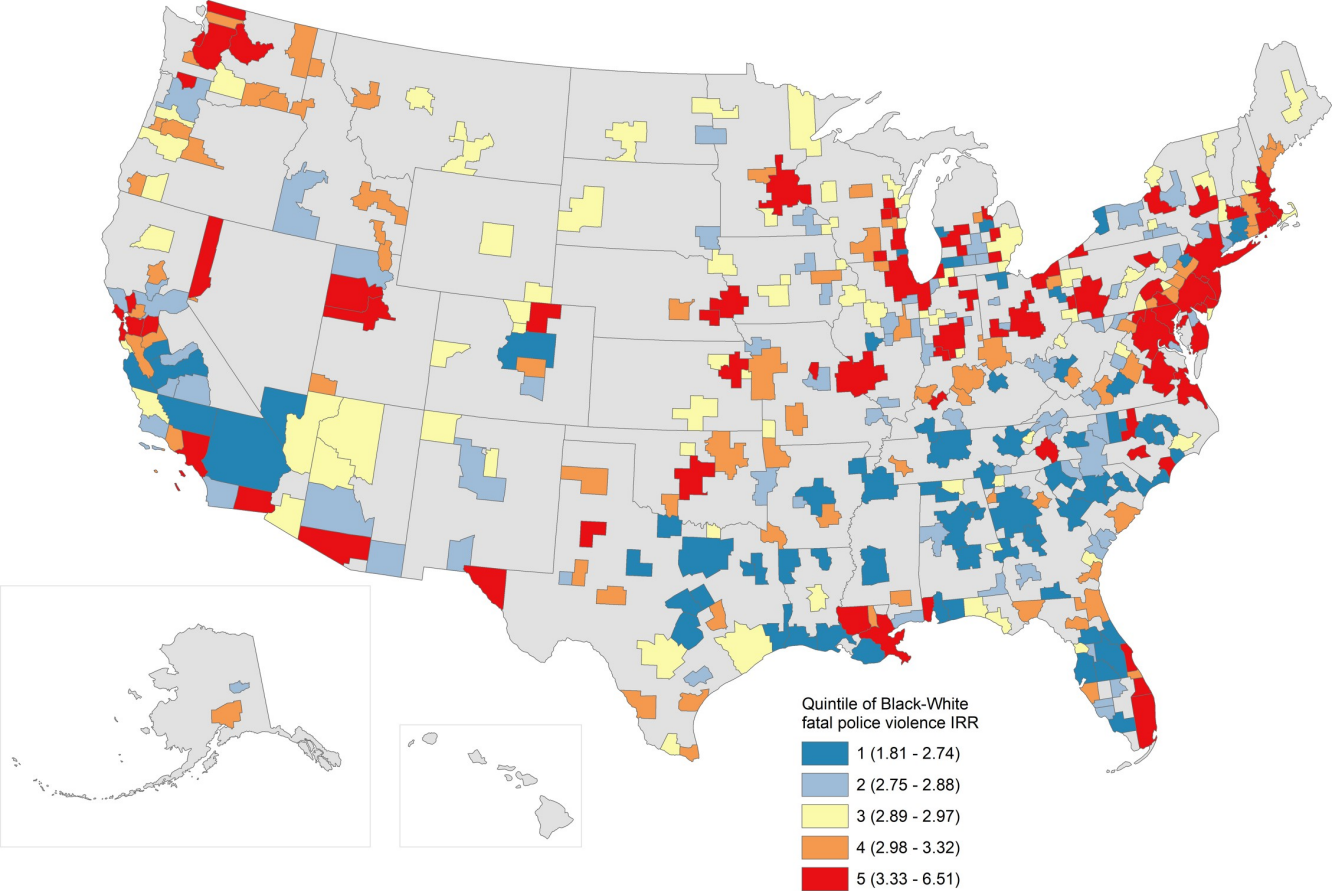
Estimated Black-White incidence rate ratios (annual) for fatal police violence, by MSA. Estimated MSA-specific incident rate ratios comparing rates of fatal police violence experienced by Black people relative to those experienced by White people are mapped. Quintiles are labeled in the legend along with, in parentheses, the range of IRR values included in that quintile.

There were clear spatial patterns to these estimates. Fig 1 displays MSAs by their estimated overall rates per 100,000 residents, colored by quintile, while Fig 2 displays MSAs by their estimated Black-White incidence rate ratios, also colored by quintile. Overall, MSAs in the western half of the country—including the Southwest, West, and Rocky Mountain states—and the South experienced much higher annual rates of fatal police violence than the northern Midwest and Northeast. Indeed, the global Moran’s I was 0.289 (p < 0.001), and the location of clusters of particularly high or particularly low MSAs map onto the patterns just described. But when examining Black-White inequities (with exceptions; e.g., Los Angeles, Seattle, the San Francisco Bay Area, or St. Louis), these patterns are reversed: the northern Midwest and Northeast exhibited the most extreme Black-White inequities in the nation, while in the South and Southwest these inequities were much lower. Black-White IRRs exhibited lower spatial autocorrelation overall, with a Moran’s I of 0.10 (p < 0.001).

**Fig 3:**
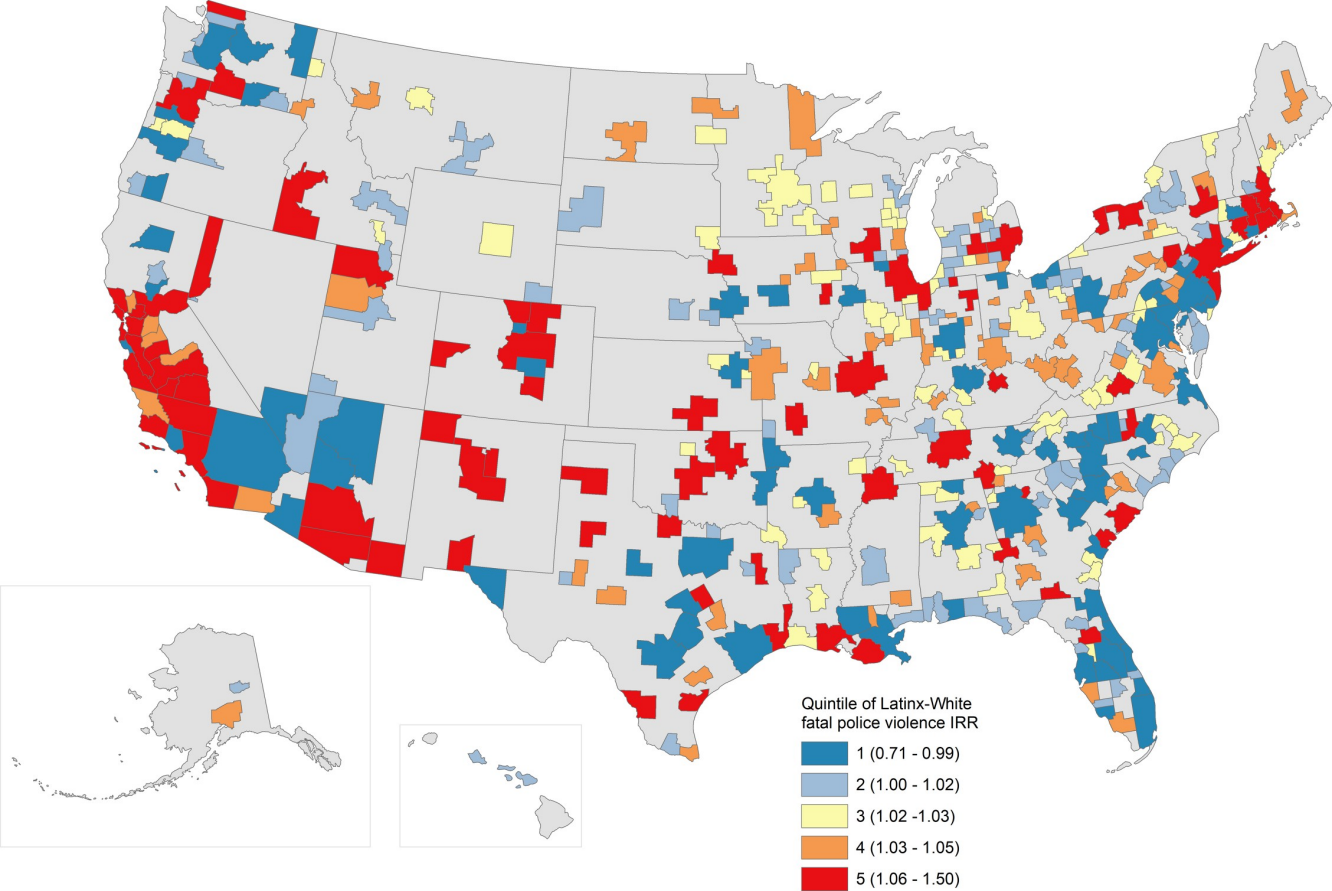
Estimated Latinx-White incidence rate ratios (annual) for fatal police violence, by MSA. Estimated MSA-specific incident rate ratios comparing rates of fatal police violence experienced by Latinx people relative to those experienced by White people are mapped. Quintiles are labeled in the legend along with, in parentheses, the range of IRR values included in that quintile.

Latinx-White IRRs are displayed in Fig 3, though we urge caution due to their lower statistical precision. Latinx-White IRRs showed relatively high ratios in the West and Northeast and variable rates across the middle of the country. Latinx-White clustering was somewhat higher than Black-White clustering overall, with a Moran’s I of 0.169 (p < 0.001), though again, the data we used to calculate it are themselves highly imprecise estimates.

Our first sensitivity analysis compared estimates from models analyzing a subset of causes of death (excluding deaths that could be considered accidents) to models analyzing all deaths (see S1–S6 Figs, S5 and S6 Tables in the Supporting information). We found that, on average, including fatalities from all causes increased MSA-specific overall fatality rates by 33%. The degree of this increase varied, from MSAs like Anniston-Oxford-Jacksonville, AL, where nearly all fatalities were non-“accidental”—yielding an increase of just 5% from non-accidental to all-cause estimated fatality rates—to Kalamazoo-Portage, MI, where the overall rate increased more than 2.5 times. Accordingly, our list of the MSAs with the highest rates and IRRs were largely the same, but new MSAs emerged with high rates of police violence in our all-cause analysis (S1 Table). For example, Kalamazoo-Portage, MI, emerged as having a particularly high rate for Black people and a wide Black-White IRR (rate: 2.03 [CI: 0.47, 8.82]; IRR: 5.23 [CI: 1.19, 23.06]).

Indeed, IRRs measuring racial/ethnic inequities in fatal police violence were often notably altered in models that included all causes of death compared to the subset included in the main analysis. On average, Black-White IRRs increased by 7% when analyzing all-cause fatalities, but this ranged from an IRR more than two times larger when analyzing all-cause fatalities vs. “accidental” fatalities in Kalamazoo-Portage, MI, to an IRR 0.83 times as large when analyzing all-cause fatalities in Seattle-Tacoma-Bellevue, WA. On average, Latinx-White IRRs increased by only 2% when analyzing all-cause fatalities, but again, geography mattered: the Latinx-White IRR was 39% higher when analyzing all-cause fatalities in Kansas City, MO-KS (the MSA with the largest proportional increase) but only 0.83 times as large when analyzing all-cause fatalities in Atlanta-Sandy Springs-Roswell, GA (the MSA with the largest proportional decrease).

Our second sensitivity analysis used MCMC estimation procedures to calculate MSA-specific rates and compared them with those calculated using Laplacian approximation as the integration method. MCMC model estimates were practically identical to those using *mepoisson*, both in terms of point estimates and standard errors.

## Discussion

Our analysis demonstrates wide geographic variation in the incidence of fatal police violence across the U.S., with the deadliest MSAs exhibiting annual rates 9 times those of the least deadly. These rates varied across the country, with the highest overall rates in the West and Southwest and the lowest overall rates in the Northeast and Midwest. We applied a precision-weighted multilevel modeling strategy to estimate the racial/ethnic inequities in these fatalities across MSAs and identify MSAs with marked Black-White inequities, and demonstrated that areas of the county with low rates overall tended to also exhibit the most severe Black-White inequities. Our findings were robust to two sets of sensitivity analyses: one that included all causes of death and another that used MCMC estimation procedures.

Monitoring these incidence rates and their racial/ethnic inequities allows public officials and the communities they represent to track the severity of the problem, devise preventive policies, and evaluate their efficacy. These monitoring efforts also have implications for racial/ethnic inequities in mental health and other health outcomes that are affected by stress from violence by state-sanctioned actors and law enforcement officer impunity [5,31]. For example, the hypervigilance and psychologic distress associated with stop and frisk policing [32] may also be greater in areas where there are known lethal consequences of police encounters.

Our results must be interpreted with the following cautions in mind. First, we have examined variation in fatal police violence across MSAs. Related research suggests this is not the only geographic level that matters: neighborhood context may be critical [2,6,7]. However, the distribution of neighborhoods’ characteristics across a given metropolitan area is determined, in part, by political, economic, and social dynamics operating at higher levels than the neighborhood (e.g., the MSA) [33,34]. Thus, it may be that differences in MSAs’ levels of neighborhood segregation, to take one example, help to explain differences in MSAs’ rates of fatal police violence; policy interventions at the MSA level may be necessary in order to alter residential segregation or other characteristics of neighborhoods.

Second, there is a potential for misclassification of race/ethnicity of the person who died because it is not self-reported, whereby people of color may be classified as White by police and media reports; this would bias racial/ethnic comparisons of the rates of fatalities involving police towards the null [35]. Third, there may also be misclassification of cause of death [11], which would bias our estimates if causes of death were inaccurately reported generally and especially if causes of death were differentially misclassified across race [3]. We also cannot definitively rule out the existence of cases that were included in our subset of the Fatal Encounters dataset but would have happened in the absence of police intervention. Our sensitivity analysis comparing all causes of death to non-“accidental” deaths found that our main results were a slightly conservative estimate of both incidence rates and racial/ethnic inequities, at least nationally speaking.

Speaking at the MSA level, however, we found that focusing exclusively on non-“accidental” deaths may yield MSA-specific rates of fatal police violence that are as little as two fifths what they would be if police-related fatalities of all causes were analyzed. MSA-specific racial inequities appeared to be similarly sensitive to the causes of death included in analyses of police-related deaths, such that only including non-“accidental” deaths may deflate estimated Black-White inequities by as much as 50% or inflate them by as much as 15%, depending on the city. How accurately causes of death are reported may thus yield dramatically different assessments of how individual cities are doing at addressing fatal police violence. This is not merely a problem of specificity; it is also a question of which kinds of transformations of policing are required to save lives. Reducing non-“accidental” police-related deaths may require different solutions (e.g., altering use of force policies, implementing de-escalation tactics, or addressing the root economic and social forces that expose marginalized people to higher levels of police contact [36]) than reducing “accidental” deaths (e.g., interventions to reduce overdoses in police custody, or deployment of mental health specialists as response teams to acute psychological crises [37]), though this question has not been directly addressed by existing research.

Continued social epidemiologic research is needed to explain the geographic distribution of fatal police violence, overall and across race/ethnicity. Place-specific policy contexts are likely a major cause of the distribution of overall incidence rates. For example, state and local firearm regulations [38], levels of segregation [7] and policy drivers of those levels [33,34], or differences in police training and police department protocols (see the following paragraph for a detailed list) may help explain different rates of fatal police violence overall. Theoretical frameworks from sociology may be a further instructive guide to understanding the racial/ethnic distribution of fatal police violence across MSAs. Decades of sociological scholarship suggest that the targeted policing of people of color, especially Black and Latinx people, serves to reify a racial social order and consequently widens racial/ethnic inequities in health outcomes, including police-related fatalities [39,40]. Sociologic, economic, and epidemiologic research on racism offers one lens to understand the geographic patterns of racial/ethnic inequities in fatal police violence observed in this paper. This work points to historically-rooted racism, manifesting, for example, in different patterns of residential segregation, criminal justice policy, and policing practices across different regions of the United States [6,41,42], as well as contemporary anti-immigrant racism, particularly in the Southwest [43]. Previous empirical evaluation of the geographic distribution of racial/ethnic inequities in police violence has pointed to the “minority threat hypothesis,” which conceptualizes fatal police violence as an exertion of social control legitimized by racialized conceptions of criminality [40,44]. A previous national, county-level analysis found that the racial distribution of police-related deaths is not explained by the racial distribution of crime in those areas [45].

A historically grounded analysis of racial justice is critical as, across the country, different strategies are being implemented to prevent fatalities involving police and racial/ethnic inequities in these deaths. There is active debate around these methods, including the effectiveness of body cameras [46–48], community policing [49,50], crisis intervention teams [51], hiring practices/officer demographics [7,52,53], law enforcement implicit bias training [54], and use/disuse of military equipment by law enforcement [55,56], among other interventions. Our analysis suggests specific MSAs where these discussions between affected communities, advocates, law enforcement, and politicians are especially urgent; places where rates of fatal police violence are relatively low, and thus where lessons may be learned; as well as areas where attention to the racial/ethnic distribution of fatal police violence is desperately needed.

## Supporting information

S1 Fig Distribution across MSAs of the ratio between estimated incidence rate using deaths from all causes and estimated incidence rate using “non-accidental” deaths. (DOCX)

S2 Fig MSA-level scatterplot of the ratio between estimated incidence rate using deaths from all causes and estimated incidence rate using “non-accidental” deaths. (DOCX)

S3 Fig Distribution across MSAs of the ratio between estimated Black-White IRR using deaths from all causes and estimated Black-White IRR using “non-accidental” deaths. (DOCX)

S4 Fig MSA-level scatterplot of the ratio between estimated Black-White IRR using deaths from all causes and estimated Black-White IRR using “non-accidental” deaths. (DOCX)

S5 Fig Distribution across MSAs of the ratio between estimated Latinx-White IRR using deaths from all causes and estimated Latinx-White IRR using “non-accidental” deaths. (DOCX)

S6 Fig MSA-level scatterplot of the ratio between estimated Latinx-White IRR using deaths from all causes and estimated Latinx-White IRR using “non-accidental” deaths. (DOCX)

S7 Fig Caterpillar plots for models estimating the overall incidence rates, race-stratified incidence rates, Black-White IRR, and Latinx-White IRR. (DOCX)

S1 Table Incidence rates of fatalities involving police per 100,000 for MSAs with the top ten highest incidence rates (no causes of death excluded), 2013–2017. (DOCX)

S2 Table Incident rate ratios and 95% confidence intervals for MSAs with largest racial inequities in fatalities involving police (no causes of death excluded), 2013–2017. (DOCX)

S3 Table Estimated annual incident rates of fatalities involving police per 100,000 by MSA (race-specific rates are from stratified models). (DOCX)

S4 Table Estimated annual IRRs comparing Black and Latinx rates of fatalities involving police to White rates by MSA. (DOCX)

S5 Table Comparing estimates of incident rates of fatalities involving police per 100,000 by MSA when using non-“accidental” vs. all-cause fatalities. (DOCX)

S6 Table Comparing estimates of incident rate ratios—comparing Black and Latinx rates to White rates—of fatalities involving police per 100,000 by MSA when using non-“accidental” vs. all-cause fatalities. (DOCX)

## Abbreviations

MSA: Metropolitan Statistical Area
MCMC: Markov chain Monte Carlo
CI: Confidence interval (throughout the paper, these are 95% confidence intervals
IR: Incidence rate
IRR: Incidence rate ratio
