# Supplementary material for: Mapping fatal police violence across U.S. metropolitan areas: Overall rates and racial/ethnic inequities, 2013-2017
Source: PLoS One. 2020 Jun 24;15(6):e0229686. doi: 10.1371/journal.pone.0229686 (PMC7313728; doi:10.1371/journal.pone.0229686)

**S2 Fig.** MSA-level scatterplot of the ratio between estimated incidence rate using deaths from all causes and estimated incidence rate using “non-accidental” deaths


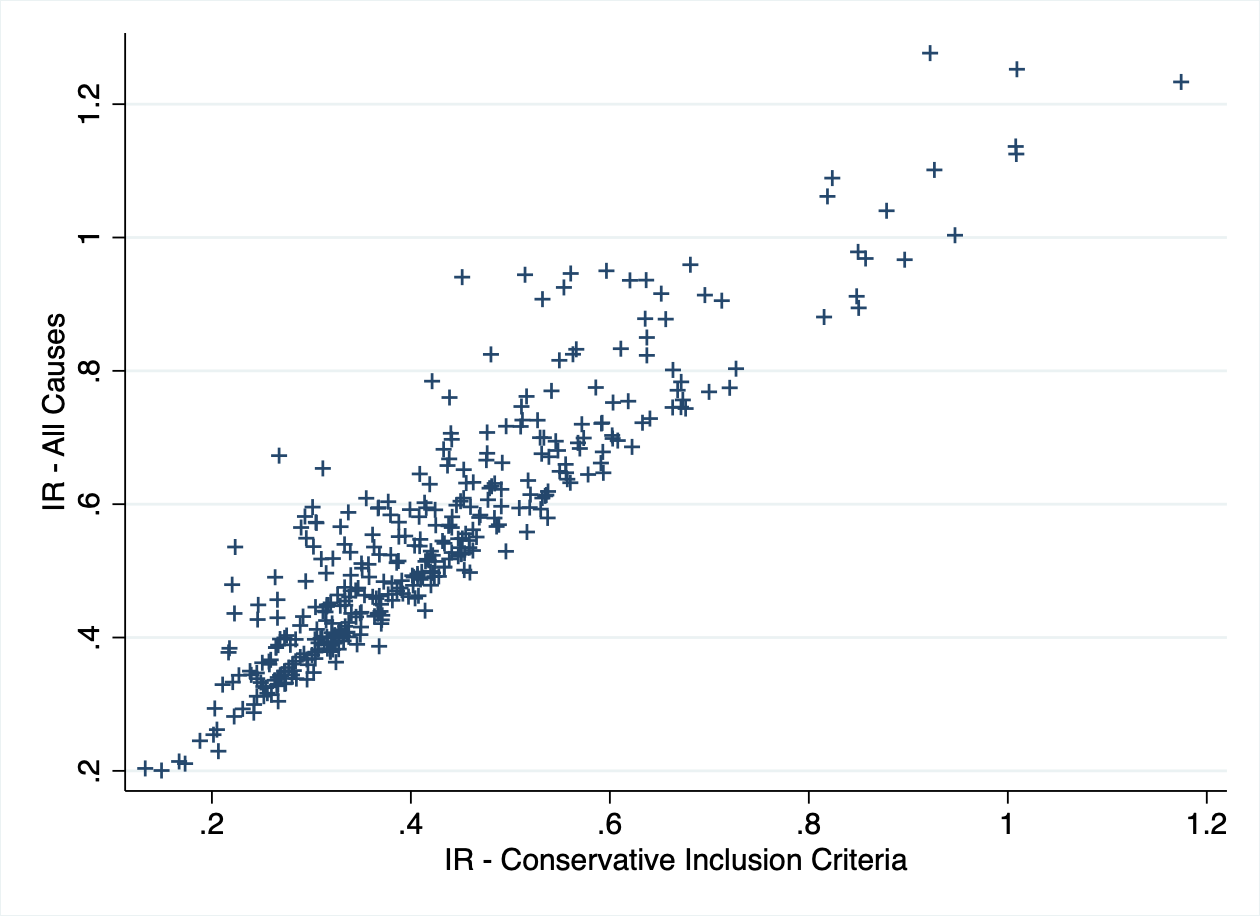

Supplement: S2 Fig — (DOCX) [file pone.0229686.s002.docx]
