# Supplementary material for: Mapping fatal police violence across U.S. metropolitan areas: Overall rates and racial/ethnic inequities, 2013-2017
Source: PLoS One. 2020 Jun 24;15(6):e0229686. doi: 10.1371/journal.pone.0229686 (PMC7313728; doi:10.1371/journal.pone.0229686)

**S3 Fig.** Distribution across MSAs of the ratio between estimated Black-White IRR using deaths from all causes and estimated Black-White IRR using “non-accidental” deaths


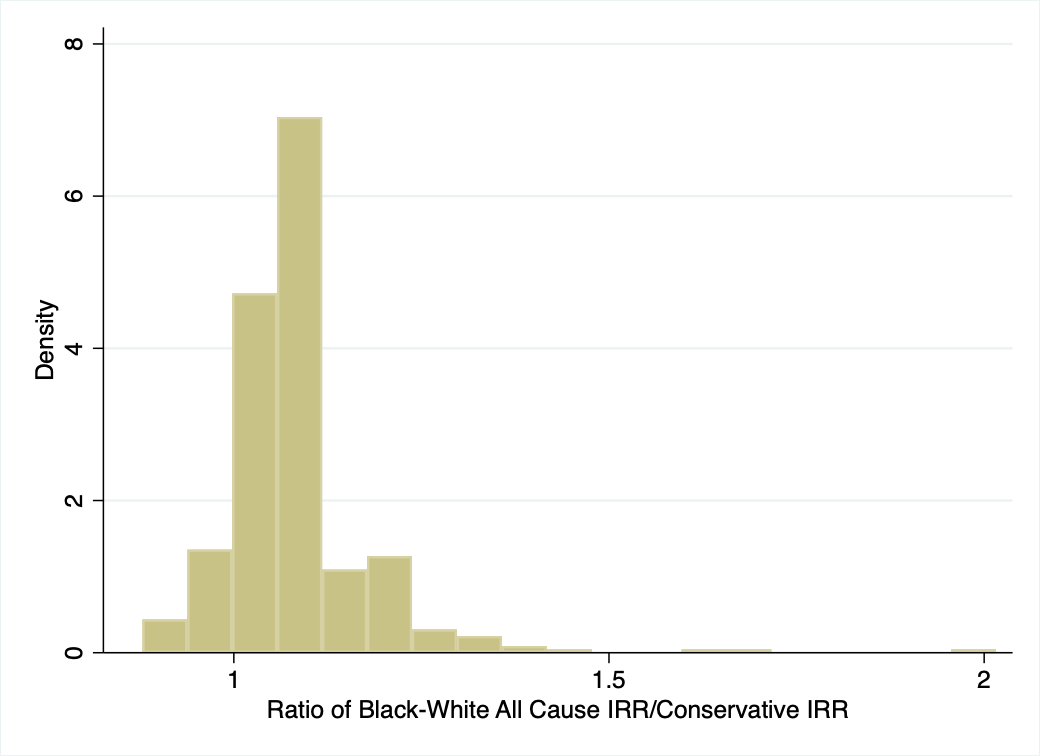

Supplement: S3 Fig — (DOCX) [file pone.0229686.s003.docx]
