# Supplementary material for: Mapping fatal police violence across U.S. metropolitan areas: Overall rates and racial/ethnic inequities, 2013-2017
Source: PLoS One. 2020 Jun 24;15(6):e0229686. doi: 10.1371/journal.pone.0229686 (PMC7313728; doi:10.1371/journal.pone.0229686)
Supplement: S7 Fig — (DOCX) [file pone.0229686.s007.docx]

**S7 Fig.** Caterpillar plots for models estimating the overall incidence rates, race-stratified incidence rates, Black-White IRR, and Latinx-White IRR

Distribution of random intercepts at the MSA level, with 95% confidence intervals, estimating the incidence of fatal police violence, overall and stratified by race

Distribution of random slopes at the MSA level, with 95% confidence intervals, estimating incidence rate ratios between race/ethnicities
