# Supplementary material for: Mapping fatal police violence across U.S. metropolitan areas: Overall rates and racial/ethnic inequities, 2013-2017
Source: PLoS One. 2020 Jun 24;15(6):e0229686. doi: 10.1371/journal.pone.0229686 (PMC7313728; doi:10.1371/journal.pone.0229686)
Supplement: S1 Table — (DOCX) [file pone.0229686.s008.docx]

**S1 Table.** Incidence rates of fatalities involving police per 100,000 for MSAs with the top ten highest incidence rates (no causes of death excluded), 2013-2017

| **Rank** | **Overall** | **Black** | **White** | **Latinx** | **Other** |
| --- | --- | --- | --- | --- | --- |
| 1 | Albuquerque, NM  1.28 (0.40, 4.11) | Oklahoma City, OK  2.64 (0.71, 9.80) | Lake Havasu City-Kingman, AZ  1.17 (0.28, 4.85) | Pueblo, CO  1.39 (0.31, 6.35) | Fairbanks, AK  1.67 (0.28, 9.91) |
| 2 | Farmington, NM  1.25 (0.31, 5.17) | San Francisco-Oakland-Hayward, CA  2.27 (0.67, 7.68) | Anniston-Oxford-Jacksonville, AL  1.10 (0.25, 4.97) | Tucson, AZ  1.20 (0.32, 4.49) | Flagstaff, AZ  1.24 (0.23, 6.78) |
| 3 | Anniston-Oxford-Jacksonville, AL  1.23 (0.30, 5.15) | Kalamazoo-Portage, MI  2.03 (0.47, 8.82) | Albuquerque, NM (1.00, 0.26, 3.77) | Wichita Falls, TX  1.17 (0.23, 5.95) | Rapid City, SD  1.06 (0.17, 6.64) |
| 4 | Billings, MT  1.14 (0.28, 4.61) | San Antonio-New Braunfels, TX  2.00 (0.53, 7.60) | Billings, MT  0.97 (0.23, 4.15) | Greenley, CO  1.10 (0.24, 5.06) | Bismarck, ND  0.68 (0.10, 4.57) |
| 5 | Bakersfield, CA  1.13 (0.34, 3.70) | Dayton, OH  1.97 (0.51, 7.66) | Yuba City, CA  0.96 (0.21, 4.35) | Albuquerque, NM  1.07 (0.29, 3.96) | Billings, MT  0.67 (0.10, 4.54) |
| 6 | Oklahoma City, OK  1.10 (0.35, 3.43) | York-Hanover, PA  1.97 (0.45, 8.67) | Deltona-Daytona Beach-Ormond Beach, FL  0.92 (0.24, 3.44) | Bakersfield, CA  0.95 (0.26, 3.56) | Redding, CA  0.59 (0.09, 3.96) |
| 7 | Redding, CA  1.09 (0.27, 4.40) | Modesto, CA  1.96 (0.43, 8.82) | Redding, CA  0.90 (0.20, 3.86) | Amarillo, TX  0.89 (0.19, 4.24) | Lafayette, LA  0.56 (0.08, 3.67) |
| 8 | Lake Havasu City-Kingman, AZ  1.06 (0.27, 4.24) | St. Louis, MO-IL  1.92 (0.59, 6.30) | Bakersfield, CA  0.86 (0.22, 3.42) | Santa Fe, NM  0.87 (0.18, 4.16) | Farmington, NM  0.55 (0.09, 3.25) |
| 9 | Tulsa, OK  1.04 (0.32, 3.41) | Tulsa, OK  1.91 (0.47, 7.80) | Tulsa, OK  0.82 (0.23, 2.97) | Phoenix-Mesa-Scottsdale, AZ  0.86 (0.27, 2.80) | Anchorage, AK  0.51 (0.09, 2.84) |
| 10 | Pueblo, CO  1.00 (0.24, 4.13) | Flint, MI  1.85 (0.46, 7.54) | Longview, TX  0.82 (0.19, 3.61) | Oklahoma City, OK  0.84 (0.19, 3.67) | Merced, CA  0.48 (0.07, 3.09) |
