# Supplementary material for: Mapping fatal police violence across U.S. metropolitan areas: Overall rates and racial/ethnic inequities, 2013-2017
Source: PLoS One. 2020 Jun 24;15(6):e0229686. doi: 10.1371/journal.pone.0229686 (PMC7313728; doi:10.1371/journal.pone.0229686)
Supplement: S2 Table — (DOCX) [file pone.0229686.s009.docx]

**S2 Table.** Incident rate ratios and 95% confidence intervals for MSAs with largest racial inequities in fatalities involving police (no causes of death excluded), 2013-2017

| **Rank** | **Black-White Inequities** | **Latinx-White Inequities** | **Other-White Inequities** |
| --- | --- | --- | --- |
| 1 | Chicago-Naperville-Elgin, IL-IN-WI  7.03 (2.06, 23.96) | Tucson, AZ  1.83 (0.47, 7.07) | Fairbanks, AK  1.95 (0.34, 11.21) |
| 2 | Flint, MI  5.72 (1.33, 24.65) | Pueblo, CO  1.77 (0.42, 7.50) | Flagstaff, AZ  1.62 (0.30, 8.80) |
| 3 | San Francisco-Oakland-Hayward, CA  5.61 (1.54, 20.40) | Greeley, CO  1.73 (0.41, 7.37) | Rapid City, SD  1.25 (0.22, 7.30) |
| 4 | Milwaukee-Waukesha-West Allis, WI  5.45 (1.35, 21.99) | Wichita Falls, TX  1.66 (0.38, 7.30) | Minneapolis-St. Paul-Bloomington, MN-WI  1.18 (0.25, 5.61) |
| 5 | St. Louis, MO-IL  5.33 (1.48, 19.27) | Kansas City, MO-KS  1.45 (0.36, 5.88) | Bismarck, ND  0.96 (0.16, 5.84) |
| 6 | Kalamazoo-Portage, MI  5.23 (1.19, 23.06) | San Francisco-Oakland-Hayward, CA  1.42 (0.39, 5.22) | Lafayette, LA  0.90 (0.15, 5.32) |
| 7 | York-Hanover, PA  5.14 (1.16, 22.86) | Phoenix-Mesa-Scottsdale, AZ  1.41 (0.41, 4.80) | Miami-Fort Lauderdale-West Palm Beach, FL  0.85 (0.17, 4.31) |
| 8 | Modesto, CA  5.09 (1.13, 22.97) | Providence-Warwick, RI-MA  1.40 (0.33, 6.03) | Anchorage, AK  0.84 (0.16, 4.50) |
| 9 | Asheville, NC  5.08 (1.13, 22.97) | San Jose-Sunnyvale-Santa Clara, CA  1.40 (0.35, 5.52) | Billings, MT  0.84 (0.14, 4.98) |
| 10 | New York-Newark-Jersey City, NY-NJ-PA  5.05 (1.50, 17.02) | Santa Rosa, CA  1.39 (0.33, 5.95) | Redding, CA  0.78 (0.13, 4.61) |
