# Supplementary material for: Mapping fatal police violence across U.S. metropolitan areas: Overall rates and racial/ethnic inequities, 2013-2017
Source: PLoS One. 2020 Jun 24;15(6):e0229686. doi: 10.1371/journal.pone.0229686 (PMC7313728; doi:10.1371/journal.pone.0229686)
Supplement: S3 Table — (DOCX) [file pone.0229686.s010.docx]

**S3 Table.** Estimated annual incident rates of fatalities involving police per 100,000 by MSA (race-specific rates are from stratified models)

| **MSA** | **MSA Name** | **Overall** | | | | **Black** | | | | **White** | | | | **Latinx** | | | |
| --- | --- | --- | --- | --- | --- | --- | --- | --- | --- | --- | --- | --- | --- | --- | --- | --- | --- |
|  |  | *Rank* | *Rate* | *95% CI (Lower Bound)* | *95% CI (Upper Bound)* | *Rank* | *Rate* | *95% CI (Lower Bound)* | *95% CI (Upper Bound)* | *Rank* | *Rate* | *95% CI (Lower Bound)* | *95% CI (Upper Bound)* | *Rank* | *Rate* | *95% CI (Lower Bound)* | *95% CI (Upper Bound)* |
| 11500 | Anniston-Oxford-Jacksonville, AL | 1 | 1.17 | 0.28 | 4.99 | 168 | 0.84 | 0.18 | 3.91 | 1 | 1.10 | 0.24 | 5.02 | 138 | 0.30 | 0.05 | 1.65 |
| 22140 | Farmington, NM | 2 | 1.01 | 0.24 | 4.31 | 146 | 0.84 | 0.17 | 4.05 | 15 | 0.61 | 0.12 | 3.08 | 12 | 0.62 | 0.12 | 3.26 |
| 12540 | Bakersfield, CA | 3 | 1.01 | 0.30 | 3.37 | 80 | 0.95 | 0.21 | 4.33 | 6 | 0.75 | 0.19 | 3.06 | 3 | 0.81 | 0.21 | 3.11 |
| 13740 | Billings, MT | 4 | 1.01 | 0.24 | 4.18 | 155 | 0.84 | 0.17 | 4.04 | 3 | 0.86 | 0.20 | 3.80 | 214 | 0.29 | 0.05 | 1.60 |
| 39380 | Pueblo, CO | 5 | 0.95 | 0.23 | 3.97 | 195 | 0.83 | 0.17 | 3.98 | 141 | 0.33 | 0.06 | 1.67 | 1 | 1.27 | 0.28 | 5.88 |
| 36420 | Oklahoma City, OK | 6 | 0.93 | 0.29 | 2.95 | 1 | 2.10 | 0.54 | 8.08 | 13 | 0.62 | 0.17 | 2.23 | 6 | 0.79 | 0.18 | 3.46 |
| 10740 | Albuquerque, NM | 7 | 0.92 | 0.27 | 3.11 | 56 | 0.99 | 0.21 | 4.62 | 8 | 0.71 | 0.18 | 2.85 | 8 | 0.77 | 0.20 | 2.98 |
| 11260 | Anchorage, AK | 8 | 0.90 | 0.24 | 3.39 | 47 | 1.02 | 0.22 | 4.77 | 55 | 0.43 | 0.10 | 1.91 | 70 | 0.35 | 0.06 | 1.85 |
| 46140 | Tulsa, OK | 9 | 0.88 | 0.26 | 2.95 | 6 | 1.45 | 0.34 | 6.17 | 9 | 0.71 | 0.19 | 2.62 | 18 | 0.50 | 0.10 | 2.47 |
| 29740 | Las Cruces, NM | 10 | 0.86 | 0.21 | 3.52 | 205 | 0.82 | 0.17 | 3.95 | 42 | 0.46 | 0.09 | 2.35 | 11 | 0.63 | 0.14 | 2.89 |
| 47380 | Waco, TX | 11 | 0.85 | 0.21 | 3.41 | 54 | 0.99 | 0.22 | 4.52 | 5 | 0.77 | 0.17 | 3.42 | 25 | 0.47 | 0.09 | 2.37 |
| 49700 | Yuba City, CA | 12 | 0.85 | 0.20 | 3.59 | 215 | 0.82 | 0.17 | 3.93 | 4 | 0.82 | 0.17 | 3.83 | 87 | 0.32 | 0.06 | 1.67 |
| 42140 | Santa Fe, NM | 13 | 0.85 | 0.20 | 3.64 | 157 | 0.84 | 0.17 | 4.04 | 43 | 0.45 | 0.09 | 2.30 | 7 | 0.78 | 0.16 | 3.76 |
| 39820 | Redding, CA | 14 | 0.82 | 0.20 | 3.47 | 187 | 0.83 | 0.17 | 4.01 | 11 | 0.70 | 0.15 | 3.15 | 275 | 0.28 | 0.05 | 1.52 |
| 29420 | Lake Havasu City-Kingman, AZ | 15 | 0.82 | 0.20 | 3.40 | 188 | 0.83 | 0.17 | 4.00 | 2 | 0.90 | 0.21 | 3.90 | 71 | 0.34 | 0.06 | 1.84 |
| 39900 | Reno, NV | 16 | 0.82 | 0.22 | 3.07 | 5 | 1.47 | 0.31 | 6.92 | 20 | 0.59 | 0.14 | 2.50 | 14 | 0.56 | 0.12 | 2.68 |
| 38060 | Phoenix-Mesa-Scottsdale, AZ | 17 | 0.73 | 0.26 | 2.04 | 11 | 1.32 | 0.34 | 5.04 | 23 | 0.56 | 0.18 | 1.77 | 9 | 0.76 | 0.23 | 2.51 |
| 39140 | Prescott, AZ | 18 | 0.72 | 0.17 | 3.01 | 163 | 0.84 | 0.17 | 4.03 | 17 | 0.61 | 0.13 | 2.72 | 331 | 0.26 | 0.05 | 1.40 |
| 46060 | Tucson, AZ | 19 | 0.71 | 0.21 | 2.45 | 9 | 1.34 | 0.30 | 6.08 | 82 | 0.40 | 0.10 | 1.65 | 4 | 0.80 | 0.20 | 3.16 |
| 22520 | Florence-Muscle Shoals, AL | 20 | 0.70 | 0.16 | 3.06 | 316 | 0.75 | 0.16 | 3.53 | 12 | 0.68 | 0.15 | 3.13 | 128 | 0.30 | 0.05 | 1.65 |
| 45820 | Topeka, KS | 21 | 0.70 | 0.17 | 2.90 | 18 | 1.21 | 0.26 | 5.67 | 74 | 0.40 | 0.09 | 1.89 | 298 | 0.27 | 0.05 | 1.47 |
| 26620 | Huntsville, AL | 22 | 0.68 | 0.18 | 2.63 | 52 | 1.00 | 0.23 | 4.28 | 40 | 0.47 | 0.11 | 2.05 | 294 | 0.27 | 0.05 | 1.47 |
| 12220 | Auburn-Opelika, AL | 23 | 0.68 | 0.16 | 2.95 | 115 | 0.89 | 0.19 | 4.08 | 102 | 0.37 | 0.08 | 1.86 | 172 | 0.29 | 0.05 | 1.63 |
| 21820 | Fairbanks, AK | 24 | 0.67 | 0.15 | 3.06 | 212 | 0.82 | 0.17 | 3.93 | 190 | 0.28 | 0.05 | 1.43 | 206 | 0.29 | 0.05 | 1.61 |
| 30980 | Longview, TX | 25 | 0.67 | 0.16 | 2.83 | 351 | 0.67 | 0.14 | 3.12 | 7 | 0.71 | 0.16 | 3.22 | 35 | 0.45 | 0.08 | 2.35 |
| 27180 | Jackson, TN | 26 | 0.67 | 0.15 | 2.98 | 59 | 0.99 | 0.22 | 4.49 | 62 | 0.42 | 0.08 | 2.09 | 167 | 0.30 | 0.05 | 1.63 |
| 49420 | Yakima, WA | 27 | 0.67 | 0.16 | 2.78 | 184 | 0.83 | 0.17 | 4.01 | 161 | 0.30 | 0.06 | 1.52 | 15 | 0.52 | 0.11 | 2.50 |
| 30780 | Little Rock-North Little Rock-Conway, AR | 28 | 0.66 | 0.18 | 2.41 | 79 | 0.96 | 0.23 | 3.92 | 29 | 0.52 | 0.13 | 2.08 | 337 | 0.25 | 0.05 | 1.36 |
| 31020 | Longview, WA | 29 | 0.66 | 0.15 | 3.01 | 51 | 1.00 | 0.21 | 4.80 | 19 | 0.59 | 0.12 | 2.86 | 217 | 0.29 | 0.05 | 1.59 |
| 17020 | Chico, CA | 30 | 0.66 | 0.16 | 2.76 | 65 | 0.97 | 0.20 | 4.67 | 22 | 0.57 | 0.12 | 2.58 | 73 | 0.34 | 0.06 | 1.81 |
| 19660 | Deltona-Daytona Beach-Ormond Beach, FL | 31 | 0.65 | 0.18 | 2.42 | 304 | 0.77 | 0.17 | 3.45 | 10 | 0.70 | 0.18 | 2.71 | 255 | 0.28 | 0.05 | 1.46 |
| 27260 | Jacksonville, FL | 32 | 0.64 | 0.19 | 2.13 | 14 | 1.26 | 0.34 | 4.68 | 39 | 0.48 | 0.13 | 1.77 | 347 | 0.25 | 0.05 | 1.26 |
| 39660 | Rapid City, SD | 33 | 0.64 | 0.14 | 2.82 | 177 | 0.83 | 0.17 | 4.02 | 32 | 0.51 | 0.11 | 2.43 | 183 | 0.29 | 0.05 | 1.62 |
| 12940 | Baton Rouge, LA | 34 | 0.64 | 0.18 | 2.29 | 33 | 1.10 | 0.29 | 4.18 | 129 | 0.34 | 0.08 | 1.46 | 332 | 0.26 | 0.05 | 1.40 |
| 32900 | Merced, CA | 35 | 0.64 | 0.15 | 2.64 | 259 | 0.80 | 0.17 | 3.80 | 130 | 0.34 | 0.07 | 1.74 | 31 | 0.45 | 0.10 | 2.15 |
| 28140 | Kansas City, MO-KS | 36 | 0.64 | 0.20 | 2.01 | 25 | 1.15 | 0.30 | 4.43 | 88 | 0.39 | 0.11 | 1.38 | 62 | 0.36 | 0.07 | 1.70 |
| 24420 | Grants Pass, OR | 37 | 0.63 | 0.14 | 2.92 | 139 | 0.84 | 0.17 | 4.07 | 27 | 0.53 | 0.11 | 2.62 | 176 | 0.29 | 0.05 | 1.63 |
| 13980 | Blacksburg-Christiansburg-Radford, VA | 38 | 0.62 | 0.14 | 2.70 | 260 | 0.80 | 0.17 | 3.80 | 34 | 0.50 | 0.11 | 2.34 | 149 | 0.30 | 0.05 | 1.64 |
| 17980 | Columbus, GA-AL | 39 | 0.62 | 0.15 | 2.54 | 354 | 0.67 | 0.15 | 2.91 | 90 | 0.39 | 0.08 | 1.86 | 57 | 0.37 | 0.07 | 1.99 |
| 12620 | Bangor, ME | 40 | 0.62 | 0.14 | 2.73 | 164 | 0.84 | 0.17 | 4.03 | 14 | 0.61 | 0.13 | 2.82 | 101 | 0.30 | 0.05 | 1.67 |
| 16620 | Charleston, WV | 41 | 0.61 | 0.14 | 2.60 | 300 | 0.77 | 0.16 | 3.65 | 33 | 0.51 | 0.11 | 2.29 | 105 | 0.30 | 0.05 | 1.67 |
| 44060 | Spokane-Spokane Valley, WA | 42 | 0.61 | 0.16 | 2.32 | 91 | 0.93 | 0.20 | 4.45 | 21 | 0.58 | 0.14 | 2.29 | 323 | 0.26 | 0.05 | 1.43 |
| 41180 | St. Louis, MO-IL | 43 | 0.60 | 0.20 | 1.85 | 3 | 1.56 | 0.46 | 5.28 | 199 | 0.27 | 0.07 | 0.96 | 41 | 0.43 | 0.09 | 2.17 |
| 29180 | Lafayette, LA | 44 | 0.60 | 0.16 | 2.34 | 105 | 0.90 | 0.21 | 3.84 | 114 | 0.36 | 0.08 | 1.60 | 54 | 0.37 | 0.07 | 2.02 |
| 44180 | Springfield, MO | 45 | 0.60 | 0.15 | 2.36 | 98 | 0.92 | 0.19 | 4.36 | 47 | 0.45 | 0.11 | 1.89 | 50 | 0.38 | 0.07 | 2.09 |
| 11100 | Amarillo, TX | 46 | 0.60 | 0.14 | 2.50 | 45 | 1.03 | 0.22 | 4.84 | 105 | 0.37 | 0.08 | 1.78 | 33 | 0.45 | 0.09 | 2.28 |
| 29820 | Las Vegas-Henderson-Paradise, NV | 47 | 0.59 | 0.19 | 1.89 | 39 | 1.07 | 0.27 | 4.21 | 16 | 0.61 | 0.17 | 2.18 | 83 | 0.32 | 0.08 | 1.34 |
| 22660 | Fort Collins, CO | 48 | 0.59 | 0.15 | 2.42 | 197 | 0.83 | 0.17 | 3.97 | 68 | 0.41 | 0.09 | 1.82 | 37 | 0.44 | 0.08 | 2.31 |
| 33260 | Midland, TX | 49 | 0.59 | 0.13 | 2.60 | 96 | 0.93 | 0.19 | 4.40 | 59 | 0.42 | 0.08 | 2.11 | 59 | 0.37 | 0.07 | 1.87 |
| 20740 | Eau Claire, WI | 50 | 0.59 | 0.13 | 2.60 | 176 | 0.83 | 0.17 | 4.02 | 31 | 0.52 | 0.11 | 2.40 | 124 | 0.30 | 0.05 | 1.65 |
| 41500 | Salinas, CA | 51 | 0.59 | 0.15 | 2.33 | 284 | 0.78 | 0.16 | 3.72 | 69 | 0.41 | 0.08 | 1.96 | 20 | 0.50 | 0.11 | 2.20 |
| 36540 | Omaha-Council Bluffs, NE-IA | 52 | 0.59 | 0.16 | 2.10 | 13 | 1.28 | 0.29 | 5.53 | 66 | 0.41 | 0.10 | 1.63 | 306 | 0.27 | 0.05 | 1.38 |
| 20100 | Dover, DE | 53 | 0.58 | 0.13 | 2.54 | 131 | 0.86 | 0.19 | 3.93 | 48 | 0.45 | 0.09 | 2.18 | 240 | 0.29 | 0.05 | 1.57 |
| 23420 | Fresno, CA | 54 | 0.57 | 0.16 | 2.04 | 144 | 0.84 | 0.18 | 3.84 | 87 | 0.39 | 0.09 | 1.74 | 34 | 0.45 | 0.11 | 1.84 |
| 19380 | Dayton, OH | 55 | 0.57 | 0.16 | 2.09 | 4 | 1.51 | 0.37 | 6.13 | 138 | 0.33 | 0.08 | 1.37 | 288 | 0.27 | 0.05 | 1.49 |
| 22180 | Fayetteville, NC | 56 | 0.57 | 0.14 | 2.29 | 23 | 1.16 | 0.28 | 4.78 | 224 | 0.25 | 0.05 | 1.25 | 346 | 0.25 | 0.05 | 1.32 |
| 33740 | Monroe, LA | 57 | 0.57 | 0.13 | 2.49 | 132 | 0.86 | 0.19 | 3.84 | 45 | 0.45 | 0.09 | 2.20 | 144 | 0.30 | 0.05 | 1.64 |
| 47300 | Visalia-Porterville, CA | 58 | 0.57 | 0.14 | 2.23 | 244 | 0.81 | 0.17 | 3.87 | 75 | 0.40 | 0.08 | 1.94 | 21 | 0.49 | 0.11 | 2.15 |
| 41700 | San Antonio-New Braunfels, TX | 59 | 0.56 | 0.18 | 1.78 | 20 | 1.18 | 0.29 | 4.78 | 25 | 0.53 | 0.14 | 1.98 | 39 | 0.44 | 0.12 | 1.57 |
| 48620 | Wichita, KS | 60 | 0.56 | 0.15 | 2.12 | 87 | 0.94 | 0.21 | 4.27 | 67 | 0.41 | 0.10 | 1.71 | 40 | 0.44 | 0.09 | 2.19 |
| 24300 | Grand Junction, CO | 61 | 0.56 | 0.13 | 2.50 | 160 | 0.84 | 0.17 | 4.04 | 57 | 0.42 | 0.09 | 2.05 | 22 | 0.49 | 0.09 | 2.62 |
| 48300 | Wenatchee, WA | 62 | 0.56 | 0.12 | 2.54 | 50 | 1.00 | 0.21 | 4.82 | 30 | 0.52 | 0.10 | 2.56 | 335 | 0.26 | 0.05 | 1.39 |
| 24500 | Great Falls, MT | 63 | 0.56 | 0.12 | 2.60 | 161 | 0.84 | 0.17 | 4.04 | 120 | 0.35 | 0.07 | 1.80 | 125 | 0.30 | 0.05 | 1.65 |
| 46700 | Vallejo-Fairfield, CA | 64 | 0.56 | 0.14 | 2.21 | 61 | 0.98 | 0.22 | 4.38 | 222 | 0.25 | 0.05 | 1.25 | 28 | 0.46 | 0.09 | 2.23 |
| 44700 | Stockton-Lodi, CA | 65 | 0.55 | 0.15 | 2.06 | 19 | 1.18 | 0.27 | 5.29 | 126 | 0.35 | 0.07 | 1.60 | 46 | 0.40 | 0.09 | 1.78 |
| 40140 | Riverside-San Bernardino-Ontario, CA | 66 | 0.55 | 0.19 | 1.60 | 32 | 1.10 | 0.29 | 4.16 | 24 | 0.56 | 0.16 | 1.89 | 51 | 0.38 | 0.11 | 1.30 |
| 47220 | Vineland-Bridgeton, NJ | 67 | 0.55 | 0.12 | 2.45 | 37 | 1.08 | 0.23 | 4.96 | 124 | 0.35 | 0.07 | 1.77 | 349 | 0.25 | 0.05 | 1.31 |
| 36220 | Odessa, TX | 68 | 0.55 | 0.12 | 2.44 | 248 | 0.81 | 0.17 | 3.85 | 18 | 0.60 | 0.12 | 3.00 | 74 | 0.34 | 0.07 | 1.71 |
| 31900 | Mansfield, OH | 69 | 0.55 | 0.12 | 2.48 | 35 | 1.08 | 0.23 | 5.13 | 98 | 0.38 | 0.08 | 1.87 | 106 | 0.30 | 0.05 | 1.67 |
| 42340 | Savannah, GA | 70 | 0.54 | 0.13 | 2.20 | 119 | 0.88 | 0.21 | 3.75 | 54 | 0.43 | 0.09 | 1.95 | 295 | 0.27 | 0.05 | 1.47 |
| 28700 | Kingsport-Bristol-Bristol, TN-VA | 71 | 0.54 | 0.13 | 2.24 | 243 | 0.81 | 0.17 | 3.87 | 84 | 0.39 | 0.09 | 1.76 | 149 | 0.30 | 0.05 | 1.64 |
| 32820 | Memphis, TN-MS-AR | 72 | 0.54 | 0.16 | 1.85 | 362 | 0.65 | 0.17 | 2.39 | 49 | 0.45 | 0.11 | 1.78 | 29 | 0.46 | 0.09 | 2.30 |
| 28940 | Knoxville, TN | 73 | 0.54 | 0.15 | 1.96 | 203 | 0.82 | 0.18 | 3.75 | 37 | 0.48 | 0.13 | 1.83 | 330 | 0.26 | 0.05 | 1.41 |
| 43420 | Sierra Vista-Douglas, AZ | 74 | 0.54 | 0.12 | 2.43 | 225 | 0.82 | 0.17 | 3.91 | 189 | 0.28 | 0.05 | 1.44 | 10 | 0.67 | 0.13 | 3.42 |
| 41140 | St. Joseph, MO-KS | 75 | 0.54 | 0.12 | 2.43 | 250 | 0.81 | 0.17 | 3.85 | 26 | 0.53 | 0.11 | 2.54 | 184 | 0.29 | 0.05 | 1.62 |
| 37620 | Parkersburg-Vienna, WV | 76 | 0.53 | 0.11 | 2.48 | 162 | 0.84 | 0.17 | 4.04 | 73 | 0.40 | 0.08 | 2.02 | 93 | 0.30 | 0.05 | 1.68 |
| 24860 | Greenville-Anderson-Mauldin, SC | 77 | 0.53 | 0.15 | 1.94 | 111 | 0.89 | 0.21 | 3.74 | 41 | 0.47 | 0.12 | 1.85 | 92 | 0.31 | 0.06 | 1.60 |
| 13900 | Bismarck, ND | 78 | 0.53 | 0.12 | 2.41 | 181 | 0.83 | 0.17 | 4.01 | 166 | 0.30 | 0.06 | 1.50 | 120 | 0.30 | 0.05 | 1.66 |
| 30020 | Lawton, OK | 79 | 0.53 | 0.12 | 2.41 | 55 | 0.99 | 0.21 | 4.62 | 52 | 0.43 | 0.09 | 2.16 | 265 | 0.28 | 0.05 | 1.53 |
| 21140 | Elkhart-Goshen, IN | 80 | 0.53 | 0.12 | 2.31 | 40 | 1.07 | 0.23 | 5.04 | 149 | 0.32 | 0.07 | 1.56 | 30 | 0.45 | 0.09 | 2.41 |
| 19300 | Daphne-Fairhope-Foley, AL | 81 | 0.53 | 0.12 | 2.31 | 321 | 0.74 | 0.16 | 3.49 | 58 | 0.42 | 0.09 | 1.98 | 218 | 0.29 | 0.05 | 1.59 |
| 35380 | New Orleans-Metairie, LA | 82 | 0.53 | 0.15 | 1.83 | 46 | 1.03 | 0.28 | 3.75 | 196 | 0.27 | 0.06 | 1.14 | 336 | 0.25 | 0.05 | 1.30 |
| 24540 | Greeley, CO | 83 | 0.52 | 0.12 | 2.19 | 63 | 0.98 | 0.20 | 4.68 | 315 | 0.20 | 0.04 | 0.99 | 2 | 0.87 | 0.18 | 4.11 |
| 27780 | Johnstown, PA | 84 | 0.52 | 0.11 | 2.35 | 220 | 0.82 | 0.17 | 3.92 | 35 | 0.49 | 0.10 | 2.34 | 104 | 0.30 | 0.05 | 1.67 |
| 25860 | Hickory-Lenoir-Morganton, NC | 85 | 0.52 | 0.13 | 2.12 | 173 | 0.84 | 0.18 | 3.90 | 28 | 0.53 | 0.12 | 2.26 | 311 | 0.27 | 0.05 | 1.45 |
| 19740 | Denver-Aurora-Lakewood, CO | 86 | 0.52 | 0.17 | 1.62 | 123 | 0.87 | 0.21 | 3.65 | 92 | 0.38 | 0.11 | 1.33 | 13 | 0.58 | 0.15 | 2.23 |
| 13460 | Bend-Redmond, OR | 87 | 0.52 | 0.12 | 2.29 | 156 | 0.84 | 0.17 | 4.04 | 97 | 0.38 | 0.08 | 1.81 | 254 | 0.28 | 0.05 | 1.55 |
| 22380 | Flagstaff, AZ | 88 | 0.51 | 0.11 | 2.33 | 180 | 0.83 | 0.17 | 4.01 | 53 | 0.43 | 0.09 | 2.15 | 283 | 0.27 | 0.05 | 1.50 |
| 13140 | Beaumont-Port Arthur, TX | 89 | 0.51 | 0.13 | 2.07 | 358 | 0.66 | 0.15 | 2.92 | 44 | 0.45 | 0.10 | 2.04 | 23 | 0.49 | 0.10 | 2.49 |
| 23460 | Gadsden, AL | 90 | 0.51 | 0.11 | 2.36 | 311 | 0.76 | 0.16 | 3.58 | 201 | 0.27 | 0.05 | 1.38 | 131 | 0.30 | 0.05 | 1.65 |
| 26420 | Houston-The Woodlands-Sugar Land, TX | 91 | 0.51 | 0.18 | 1.43 | 36 | 1.08 | 0.34 | 3.45 | 80 | 0.40 | 0.12 | 1.33 | 187 | 0.29 | 0.08 | 1.02 |
| 37340 | Palm Bay-Melbourne-Titusville, FL | 92 | 0.51 | 0.13 | 1.98 | 15 | 1.26 | 0.29 | 5.53 | 108 | 0.37 | 0.09 | 1.58 | 356 | 0.24 | 0.04 | 1.26 |
| 25060 | Gulfport-Biloxi-Pascagoula, MS | 93 | 0.50 | 0.12 | 2.03 | 122 | 0.87 | 0.20 | 3.84 | 61 | 0.42 | 0.09 | 1.87 | 290 | 0.27 | 0.05 | 1.48 |
| 16860 | Chattanooga, TN-GA | 94 | 0.50 | 0.13 | 1.94 | 324 | 0.73 | 0.16 | 3.28 | 81 | 0.40 | 0.09 | 1.70 | 60 | 0.36 | 0.07 | 1.95 |
| 28660 | Killeen-Temple, TX | 95 | 0.49 | 0.12 | 1.99 | 337 | 0.71 | 0.16 | 3.15 | 111 | 0.36 | 0.08 | 1.69 | 324 | 0.26 | 0.05 | 1.35 |
| 10500 | Albany, GA | 96 | 0.49 | 0.11 | 2.21 | 273 | 0.79 | 0.18 | 3.51 | 177 | 0.28 | 0.05 | 1.48 | 135 | 0.30 | 0.05 | 1.65 |
| 31080 | Los Angeles-Long Beach-Anaheim, CA | 97 | 0.49 | 0.19 | 1.27 | 7 | 1.38 | 0.43 | 4.44 | 125 | 0.35 | 0.11 | 1.09 | 16 | 0.52 | 0.18 | 1.48 |
| 34620 | Muncie, IN | 98 | 0.49 | 0.11 | 2.25 | 261 | 0.80 | 0.17 | 3.79 | 93 | 0.38 | 0.08 | 1.90 | 116 | 0.30 | 0.05 | 1.66 |
| 36980 | Owensboro, KY | 99 | 0.49 | 0.11 | 2.24 | 72 | 0.96 | 0.20 | 4.61 | 100 | 0.38 | 0.08 | 1.86 | 121 | 0.30 | 0.05 | 1.66 |
| 41660 | San Angelo, TX | 100 | 0.48 | 0.11 | 2.23 | 74 | 0.96 | 0.20 | 4.60 | 116 | 0.36 | 0.07 | 1.85 | 84 | 0.32 | 0.06 | 1.70 |
| 28420 | Kennewick-Richland, WA | 101 | 0.48 | 0.11 | 2.06 | 73 | 0.96 | 0.20 | 4.61 | 36 | 0.48 | 0.10 | 2.22 | 287 | 0.27 | 0.05 | 1.41 |
| 17420 | Cleveland, TN | 102 | 0.48 | 0.10 | 2.21 | 233 | 0.81 | 0.17 | 3.90 | 101 | 0.38 | 0.08 | 1.86 | 181 | 0.29 | 0.05 | 1.62 |
| 16220 | Casper, WY | 103 | 0.48 | 0.10 | 2.27 | 158 | 0.84 | 0.17 | 4.04 | 50 | 0.44 | 0.09 | 2.21 | 188 | 0.29 | 0.05 | 1.62 |
| 13220 | Beckley, WV | 104 | 0.48 | 0.10 | 2.20 | 92 | 0.93 | 0.20 | 4.44 | 106 | 0.37 | 0.08 | 1.84 | 98 | 0.30 | 0.05 | 1.67 |
| 12580 | Baltimore-Columbia-Towson, MD | 105 | 0.48 | 0.15 | 1.51 | 69 | 0.97 | 0.28 | 3.28 | 232 | 0.25 | 0.07 | 0.94 | 363 | 0.23 | 0.04 | 1.13 |
| 16700 | Charleston-North Charleston, SC | 106 | 0.48 | 0.13 | 1.80 | 90 | 0.94 | 0.23 | 3.79 | 180 | 0.28 | 0.06 | 1.23 | 78 | 0.33 | 0.06 | 1.76 |
| 33700 | Modesto, CA | 107 | 0.48 | 0.12 | 1.88 | 109 | 0.90 | 0.19 | 4.23 | 78 | 0.40 | 0.09 | 1.83 | 64 | 0.35 | 0.08 | 1.64 |
| 27140 | Jackson, MS | 108 | 0.48 | 0.12 | 1.86 | 361 | 0.65 | 0.16 | 2.64 | 65 | 0.41 | 0.09 | 1.85 | 249 | 0.28 | 0.05 | 1.55 |
| 45220 | Tallahassee, FL | 109 | 0.47 | 0.11 | 1.94 | 116 | 0.89 | 0.21 | 3.78 | 193 | 0.27 | 0.06 | 1.31 | 301 | 0.27 | 0.05 | 1.46 |
| 26380 | Houma-Thibodaux, LA | 110 | 0.47 | 0.11 | 2.06 | 283 | 0.78 | 0.17 | 3.62 | 94 | 0.38 | 0.08 | 1.83 | 48 | 0.39 | 0.07 | 2.16 |
| 17900 | Columbia, SC | 111 | 0.47 | 0.12 | 1.75 | 348 | 0.68 | 0.17 | 2.74 | 123 | 0.35 | 0.08 | 1.49 | 344 | 0.25 | 0.05 | 1.32 |
| 23060 | Fort Wayne, IN | 112 | 0.46 | 0.11 | 1.88 | 16 | 1.24 | 0.28 | 5.57 | 234 | 0.25 | 0.05 | 1.15 | 65 | 0.35 | 0.07 | 1.89 |
| 31340 | Lynchburg, VA | 113 | 0.46 | 0.11 | 1.99 | 319 | 0.74 | 0.16 | 3.41 | 137 | 0.33 | 0.07 | 1.57 | 44 | 0.40 | 0.07 | 2.23 |
| 17860 | Columbia, MO | 114 | 0.46 | 0.10 | 2.07 | 44 | 1.03 | 0.22 | 4.86 | 135 | 0.33 | 0.07 | 1.63 | 169 | 0.29 | 0.05 | 1.63 |
| 34820 | Myrtle Beach-Conway-North Myrtle Beach, SC-NC | 115 | 0.46 | 0.11 | 1.87 | 338 | 0.71 | 0.15 | 3.22 | 85 | 0.39 | 0.09 | 1.72 | 305 | 0.27 | 0.05 | 1.46 |
| 37860 | Pensacola-Ferry Pass-Brent, FL | 116 | 0.46 | 0.11 | 1.85 | 363 | 0.64 | 0.14 | 2.89 | 38 | 0.48 | 0.11 | 2.04 | 312 | 0.27 | 0.05 | 1.45 |
| 48140 | Wausau, WI | 117 | 0.46 | 0.10 | 2.10 | 154 | 0.84 | 0.17 | 4.04 | 117 | 0.36 | 0.07 | 1.75 | 129 | 0.30 | 0.05 | 1.65 |
| 38220 | Pine Bluff, AR | 118 | 0.46 | 0.10 | 2.15 | 82 | 0.95 | 0.21 | 4.32 | 257 | 0.24 | 0.04 | 1.25 | 103 | 0.30 | 0.05 | 1.67 |
| 20940 | El Centro, CA | 119 | 0.46 | 0.10 | 2.04 | 70 | 0.97 | 0.20 | 4.63 | 217 | 0.26 | 0.05 | 1.40 | 77 | 0.33 | 0.07 | 1.63 |
| 16180 | Carson City, NV | 120 | 0.46 | 0.09 | 2.20 | 149 | 0.84 | 0.17 | 4.05 | 64 | 0.42 | 0.08 | 2.18 | 245 | 0.28 | 0.05 | 1.56 |
| 10420 | Akron, OH | 121 | 0.45 | 0.12 | 1.74 | 75 | 0.96 | 0.22 | 4.18 | 145 | 0.33 | 0.08 | 1.37 | 250 | 0.28 | 0.05 | 1.55 |
| 18140 | Columbus, OH | 122 | 0.45 | 0.14 | 1.51 | 10 | 1.33 | 0.36 | 4.91 | 246 | 0.24 | 0.06 | 0.92 | 261 | 0.28 | 0.05 | 1.45 |
| 49620 | York-Hanover, PA | 123 | 0.45 | 0.11 | 1.84 | 31 | 1.11 | 0.24 | 5.16 | 169 | 0.30 | 0.07 | 1.33 | 326 | 0.26 | 0.05 | 1.41 |
| 16940 | Cheyenne, WY | 124 | 0.45 | 0.10 | 2.12 | 192 | 0.83 | 0.17 | 3.98 | 56 | 0.42 | 0.08 | 2.13 | 257 | 0.28 | 0.05 | 1.55 |
| 29700 | Laredo, TX | 125 | 0.45 | 0.11 | 1.94 | 147 | 0.84 | 0.17 | 4.05 | 104 | 0.37 | 0.07 | 2.03 | 49 | 0.39 | 0.08 | 1.76 |
| 26300 | Hot Springs, AR | 126 | 0.45 | 0.10 | 2.11 | 258 | 0.80 | 0.17 | 3.80 | 133 | 0.34 | 0.07 | 1.71 | 160 | 0.30 | 0.05 | 1.63 |
| 34900 | Napa, CA | 127 | 0.45 | 0.10 | 2.06 | 62 | 0.98 | 0.20 | 4.69 | 195 | 0.27 | 0.05 | 1.41 | 86 | 0.32 | 0.06 | 1.69 |
| 26140 | Homosassa Springs, FL | 128 | 0.45 | 0.10 | 2.05 | 211 | 0.82 | 0.17 | 3.93 | 60 | 0.42 | 0.09 | 2.02 | 203 | 0.29 | 0.05 | 1.61 |
| 47020 | Victoria, TX | 129 | 0.45 | 0.10 | 2.10 | 238 | 0.81 | 0.17 | 3.87 | 83 | 0.39 | 0.08 | 2.05 | 82 | 0.32 | 0.06 | 1.71 |
| 31860 | Mankato-North Mankato, MN | 130 | 0.45 | 0.10 | 2.10 | 202 | 0.83 | 0.17 | 3.96 | 77 | 0.40 | 0.08 | 2.01 | 127 | 0.30 | 0.05 | 1.65 |
| 19140 | Dalton, GA | 131 | 0.45 | 0.10 | 2.04 | 222 | 0.82 | 0.17 | 3.92 | 150 | 0.32 | 0.06 | 1.61 | 79 | 0.33 | 0.06 | 1.75 |
| 18580 | Corpus Christi, TX | 132 | 0.45 | 0.11 | 1.82 | 113 | 0.89 | 0.19 | 4.21 | 148 | 0.32 | 0.07 | 1.56 | 53 | 0.37 | 0.08 | 1.70 |
| 24220 | Grand Forks, ND-MN | 133 | 0.45 | 0.10 | 2.08 | 199 | 0.83 | 0.17 | 3.97 | 214 | 0.26 | 0.05 | 1.34 | 150 | 0.30 | 0.05 | 1.64 |
| 33660 | Mobile, AL | 134 | 0.44 | 0.11 | 1.82 | 78 | 0.96 | 0.23 | 3.99 | 343 | 0.18 | 0.04 | 0.89 | 234 | 0.29 | 0.05 | 1.57 |
| 40900 | Sacramento--Roseville--Arden-Arcade, CA | 135 | 0.44 | 0.13 | 1.45 | 94 | 0.93 | 0.22 | 3.86 | 113 | 0.36 | 0.10 | 1.35 | 24 | 0.47 | 0.11 | 1.93 |
| 25620 | Hattiesburg, MS | 136 | 0.44 | 0.10 | 2.01 | 133 | 0.86 | 0.19 | 3.91 | 229 | 0.25 | 0.05 | 1.29 | 134 | 0.30 | 0.05 | 1.65 |
| 43900 | Spartanburg, SC | 137 | 0.44 | 0.10 | 1.86 | 169 | 0.84 | 0.19 | 3.73 | 115 | 0.36 | 0.08 | 1.67 | 286 | 0.27 | 0.05 | 1.49 |
| 21420 | Enid, OK | 138 | 0.44 | 0.09 | 2.11 | 186 | 0.83 | 0.17 | 4.01 | 86 | 0.39 | 0.08 | 2.04 | 200 | 0.29 | 0.05 | 1.61 |
| 25260 | Hanford-Corcoran, CA | 139 | 0.44 | 0.10 | 2.00 | 272 | 0.79 | 0.17 | 3.77 | 162 | 0.30 | 0.06 | 1.58 | 66 | 0.35 | 0.07 | 1.79 |
| 21060 | Elizabethtown-Fort Knox, KY | 140 | 0.44 | 0.10 | 2.00 | 108 | 0.90 | 0.19 | 4.23 | 172 | 0.29 | 0.06 | 1.46 | 205 | 0.29 | 0.05 | 1.61 |
| 45940 | Trenton, NJ | 141 | 0.44 | 0.11 | 1.83 | 8 | 1.36 | 0.32 | 5.82 | 314 | 0.20 | 0.04 | 0.99 | 360 | 0.23 | 0.04 | 1.22 |
| 13820 | Birmingham-Hoover, AL | 142 | 0.44 | 0.12 | 1.58 | 349 | 0.67 | 0.17 | 2.68 | 99 | 0.38 | 0.09 | 1.49 | 352 | 0.24 | 0.05 | 1.29 |
| 48660 | Wichita Falls, TX | 143 | 0.44 | 0.10 | 1.99 | 300 | 0.77 | 0.16 | 3.65 | 321 | 0.20 | 0.04 | 1.01 | 5 | 0.79 | 0.15 | 4.14 |
| 37460 | Panama City, FL | 144 | 0.44 | 0.10 | 1.94 | 137 | 0.85 | 0.18 | 3.95 | 147 | 0.32 | 0.07 | 1.57 | 239 | 0.29 | 0.05 | 1.57 |
| 19100 | Dallas-Fort Worth-Arlington, TX | 145 | 0.43 | 0.15 | 1.23 | 353 | 0.67 | 0.20 | 2.30 | 72 | 0.40 | 0.13 | 1.28 | 58 | 0.37 | 0.11 | 1.27 |
| 10780 | Alexandria, LA | 146 | 0.43 | 0.10 | 1.97 | 148 | 0.84 | 0.18 | 3.83 | 152 | 0.31 | 0.06 | 1.59 | 156 | 0.30 | 0.05 | 1.64 |
| 31460 | Madera, CA | 147 | 0.43 | 0.10 | 1.97 | 227 | 0.82 | 0.17 | 3.91 | 96 | 0.38 | 0.07 | 1.96 | 72 | 0.34 | 0.07 | 1.73 |
| 35660 | Niles-Benton Harbor, MI | 148 | 0.43 | 0.10 | 1.97 | 29 | 1.12 | 0.24 | 5.19 | 252 | 0.24 | 0.05 | 1.21 | 208 | 0.29 | 0.05 | 1.60 |
| 27740 | Johnson City, TN | 149 | 0.43 | 0.10 | 1.92 | 237 | 0.81 | 0.17 | 3.88 | 76 | 0.40 | 0.09 | 1.89 | 174 | 0.29 | 0.05 | 1.63 |
| 49740 | Yuma, AZ | 150 | 0.43 | 0.10 | 1.90 | 208 | 0.82 | 0.17 | 3.94 | 46 | 0.45 | 0.09 | 2.28 | 162 | 0.30 | 0.06 | 1.48 |
| 34100 | Morristown, TN | 151 | 0.43 | 0.09 | 1.97 | 201 | 0.83 | 0.17 | 3.96 | 153 | 0.31 | 0.06 | 1.57 | 219 | 0.29 | 0.05 | 1.59 |
| 26900 | Indianapolis-Carmel-Anderson, IN | 152 | 0.42 | 0.13 | 1.43 | 26 | 1.15 | 0.30 | 4.33 | 179 | 0.28 | 0.08 | 1.06 | 354 | 0.24 | 0.05 | 1.21 |
| 45300 | Tampa-St. Petersburg-Clearwater, FL | 153 | 0.42 | 0.13 | 1.35 | 322 | 0.74 | 0.19 | 2.88 | 51 | 0.43 | 0.13 | 1.47 | 369 | 0.21 | 0.05 | 0.94 |
| 35840 | North Port-Sarasota-Bradenton, FL | 154 | 0.42 | 0.11 | 1.62 | 93 | 0.93 | 0.21 | 4.20 | 132 | 0.34 | 0.08 | 1.40 | 76 | 0.33 | 0.07 | 1.69 |
| 14260 | Boise City, ID | 155 | 0.42 | 0.11 | 1.64 | 246 | 0.81 | 0.17 | 3.86 | 112 | 0.36 | 0.09 | 1.51 | 17 | 0.50 | 0.10 | 2.49 |
| 42200 | Santa Maria-Santa Barbara, CA | 156 | 0.42 | 0.10 | 1.73 | 256 | 0.80 | 0.17 | 3.82 | 181 | 0.28 | 0.06 | 1.35 | 32 | 0.45 | 0.10 | 2.09 |
| 43340 | Shreveport-Bossier City, LA | 157 | 0.42 | 0.10 | 1.73 | 344 | 0.69 | 0.16 | 2.92 | 165 | 0.30 | 0.06 | 1.41 | 269 | 0.28 | 0.05 | 1.52 |
| 39340 | Provo-Orem, UT | 158 | 0.42 | 0.11 | 1.67 | 64 | 0.98 | 0.20 | 4.68 | 110 | 0.36 | 0.09 | 1.54 | 168 | 0.29 | 0.06 | 1.54 |
| 32780 | Medford, OR | 159 | 0.42 | 0.09 | 1.86 | 167 | 0.84 | 0.17 | 4.03 | 63 | 0.42 | 0.09 | 1.95 | 314 | 0.27 | 0.05 | 1.44 |
| 48260 | Weirton-Steubenville, WV-OH | 160 | 0.42 | 0.09 | 1.95 | 27 | 1.13 | 0.24 | 5.40 | 244 | 0.24 | 0.05 | 1.23 | 97 | 0.30 | 0.05 | 1.68 |
| 12260 | Augusta-Richmond County, GA-SC | 161 | 0.42 | 0.11 | 1.66 | 376 | 0.53 | 0.12 | 2.26 | 71 | 0.41 | 0.09 | 1.78 | 328 | 0.26 | 0.05 | 1.41 |
| 18880 | Crestview-Fort Walton Beach-Destin, FL | 162 | 0.42 | 0.10 | 1.82 | 136 | 0.85 | 0.18 | 3.96 | 140 | 0.33 | 0.07 | 1.56 | 291 | 0.27 | 0.05 | 1.48 |
| 36260 | Ogden-Clearfield, UT | 163 | 0.42 | 0.11 | 1.63 | 252 | 0.80 | 0.17 | 3.84 | 103 | 0.37 | 0.09 | 1.55 | 38 | 0.44 | 0.09 | 2.20 |
| 42220 | Santa Rosa, CA | 164 | 0.42 | 0.10 | 1.68 | 253 | 0.80 | 0.17 | 3.83 | 220 | 0.25 | 0.05 | 1.18 | 19 | 0.50 | 0.10 | 2.37 |
| 38940 | Port St. Lucie, FL | 165 | 0.41 | 0.10 | 1.70 | 21 | 1.17 | 0.27 | 5.13 | 269 | 0.23 | 0.05 | 1.08 | 365 | 0.22 | 0.04 | 1.17 |
| 21340 | El Paso, TX | 166 | 0.41 | 0.11 | 1.57 | 34 | 1.09 | 0.24 | 5.05 | 156 | 0.31 | 0.06 | 1.55 | 91 | 0.31 | 0.07 | 1.27 |
| 26580 | Huntington-Ashland, WV-KY-OH | 167 | 0.41 | 0.10 | 1.74 | 262 | 0.80 | 0.17 | 3.79 | 238 | 0.25 | 0.05 | 1.14 | 137 | 0.30 | 0.05 | 1.65 |
| 16060 | Carbondale-Marion, IL | 168 | 0.41 | 0.09 | 1.91 | 288 | 0.78 | 0.16 | 3.71 | 157 | 0.31 | 0.06 | 1.55 | 140 | 0.30 | 0.05 | 1.65 |
| 14500 | Boulder, CO | 169 | 0.41 | 0.10 | 1.75 | 194 | 0.83 | 0.17 | 3.98 | 91 | 0.39 | 0.08 | 1.76 | 345 | 0.25 | 0.05 | 1.32 |
| 27860 | Jonesboro, AR | 170 | 0.41 | 0.09 | 1.90 | 309 | 0.76 | 0.16 | 3.59 | 154 | 0.31 | 0.06 | 1.56 | 170 | 0.29 | 0.05 | 1.63 |
| 35980 | Norwich-New London, CT | 171 | 0.41 | 0.09 | 1.78 | 112 | 0.89 | 0.19 | 4.22 | 146 | 0.33 | 0.07 | 1.54 | 318 | 0.26 | 0.05 | 1.43 |
| 29020 | Kokomo, IN | 172 | 0.41 | 0.09 | 1.94 | 239 | 0.81 | 0.17 | 3.87 | 121 | 0.35 | 0.07 | 1.79 | 115 | 0.30 | 0.05 | 1.66 |
| 42660 | Seattle-Tacoma-Bellevue, WA | 173 | 0.41 | 0.13 | 1.27 | 17 | 1.23 | 0.31 | 4.82 | 164 | 0.30 | 0.09 | 1.04 | 284 | 0.27 | 0.06 | 1.25 |
| 27900 | Joplin, MO | 174 | 0.41 | 0.09 | 1.84 | 200 | 0.83 | 0.17 | 3.97 | 202 | 0.27 | 0.05 | 1.32 | 242 | 0.28 | 0.05 | 1.56 |
| 12100 | Atlantic City-Hammonton, NJ | 175 | 0.41 | 0.09 | 1.77 | 28 | 1.12 | 0.25 | 5.09 | 207 | 0.26 | 0.05 | 1.31 | 353 | 0.24 | 0.05 | 1.28 |
| 25420 | Harrisburg-Carlisle, PA | 176 | 0.41 | 0.10 | 1.63 | 30 | 1.12 | 0.25 | 4.94 | 293 | 0.21 | 0.05 | 0.96 | 334 | 0.26 | 0.05 | 1.39 |
| 38540 | Pocatello, ID | 177 | 0.41 | 0.09 | 1.93 | 142 | 0.84 | 0.17 | 4.06 | 191 | 0.28 | 0.05 | 1.43 | 195 | 0.29 | 0.05 | 1.62 |
| 23540 | Gainesville, FL | 178 | 0.40 | 0.09 | 1.75 | 102 | 0.91 | 0.20 | 4.12 | 167 | 0.30 | 0.06 | 1.45 | 309 | 0.27 | 0.05 | 1.45 |
| 30860 | Logan, UT-ID | 179 | 0.40 | 0.09 | 1.86 | 152 | 0.84 | 0.17 | 4.04 | 109 | 0.37 | 0.07 | 1.80 | 251 | 0.28 | 0.05 | 1.55 |
| 21300 | Elmira, NY | 180 | 0.40 | 0.08 | 1.91 | 232 | 0.81 | 0.17 | 3.90 | 127 | 0.34 | 0.07 | 1.75 | 114 | 0.30 | 0.05 | 1.66 |
| 49020 | Winchester, VA-WV | 181 | 0.40 | 0.09 | 1.86 | 84 | 0.95 | 0.20 | 4.53 | 163 | 0.30 | 0.06 | 1.51 | 236 | 0.29 | 0.05 | 1.57 |
| 41620 | Salt Lake City, UT | 182 | 0.40 | 0.11 | 1.46 | 48 | 1.00 | 0.21 | 4.69 | 119 | 0.35 | 0.09 | 1.38 | 75 | 0.34 | 0.07 | 1.59 |
| 34060 | Morgantown, WV | 183 | 0.40 | 0.09 | 1.84 | 234 | 0.81 | 0.17 | 3.89 | 118 | 0.35 | 0.07 | 1.73 | 117 | 0.30 | 0.05 | 1.66 |
| 10900 | Allentown-Bethlehem-Easton, PA-NJ | 184 | 0.40 | 0.10 | 1.52 | 124 | 0.87 | 0.19 | 3.99 | 143 | 0.33 | 0.08 | 1.36 | 355 | 0.24 | 0.05 | 1.21 |
| 41860 | San Francisco-Oakland-Hayward, CA | 185 | 0.39 | 0.13 | 1.20 | 2 | 1.85 | 0.53 | 6.47 | 208 | 0.26 | 0.07 | 0.97 | 42 | 0.43 | 0.11 | 1.59 |
| 46660 | Valdosta, GA | 186 | 0.39 | 0.09 | 1.80 | 327 | 0.73 | 0.16 | 3.32 | 200 | 0.27 | 0.05 | 1.38 | 45 | 0.40 | 0.07 | 2.19 |
| 17820 | Colorado Springs, CO | 187 | 0.39 | 0.10 | 1.53 | 125 | 0.87 | 0.19 | 3.99 | 159 | 0.30 | 0.07 | 1.31 | 341 | 0.25 | 0.05 | 1.28 |
| 16020 | Cape Girardeau, MO-IL | 188 | 0.39 | 0.08 | 1.84 | 264 | 0.79 | 0.17 | 3.79 | 136 | 0.33 | 0.07 | 1.69 | 102 | 0.30 | 0.05 | 1.67 |
| 12060 | Atlanta-Sandy Springs-Roswell, GA | 189 | 0.39 | 0.13 | 1.15 | 380 | 0.49 | 0.15 | 1.64 | 142 | 0.33 | 0.10 | 1.10 | 381 | 0.12 | 0.02 | 0.56 |
| 20020 | Dothan, AL | 190 | 0.39 | 0.08 | 1.78 | 285 | 0.78 | 0.17 | 3.62 | 155 | 0.31 | 0.06 | 1.55 | 151 | 0.30 | 0.05 | 1.64 |
| 40580 | Rocky Mount, NC | 191 | 0.39 | 0.08 | 1.78 | 356 | 0.67 | 0.15 | 3.01 | 183 | 0.28 | 0.05 | 1.45 | 213 | 0.29 | 0.05 | 1.60 |
| 19060 | Cumberland, MD-WV | 192 | 0.39 | 0.08 | 1.82 | 251 | 0.80 | 0.17 | 3.84 | 216 | 0.26 | 0.05 | 1.33 | 96 | 0.30 | 0.05 | 1.68 |
| 45500 | Texarkana, TX-AR | 193 | 0.39 | 0.08 | 1.77 | 114 | 0.89 | 0.19 | 4.09 | 231 | 0.25 | 0.05 | 1.28 | 215 | 0.29 | 0.05 | 1.60 |
| 41420 | Salem, OR | 194 | 0.38 | 0.09 | 1.60 | 216 | 0.82 | 0.17 | 3.93 | 79 | 0.40 | 0.09 | 1.79 | 321 | 0.26 | 0.05 | 1.36 |
| 33100 | Miami-Fort Lauderdale-West Palm Beach, FL | 195 | 0.38 | 0.13 | 1.12 | 129 | 0.86 | 0.26 | 2.81 | 226 | 0.25 | 0.07 | 0.93 | 375 | 0.19 | 0.05 | 0.71 |
| 13020 | Bay City, MI | 196 | 0.38 | 0.08 | 1.79 | 57 | 0.99 | 0.21 | 4.75 | 221 | 0.25 | 0.05 | 1.30 | 165 | 0.30 | 0.05 | 1.63 |
| 31180 | Lubbock, TX | 197 | 0.38 | 0.09 | 1.64 | 60 | 0.99 | 0.21 | 4.60 | 355 | 0.17 | 0.03 | 0.86 | 26 | 0.46 | 0.09 | 2.27 |
| 25180 | Hagerstown-Martinsburg, MD-WV | 198 | 0.38 | 0.09 | 1.66 | 68 | 0.97 | 0.21 | 4.51 | 329 | 0.19 | 0.04 | 0.94 | 232 | 0.29 | 0.05 | 1.57 |
| 40220 | Roanoke, VA | 199 | 0.38 | 0.09 | 1.63 | 126 | 0.86 | 0.19 | 3.96 | 348 | 0.18 | 0.04 | 0.88 | 238 | 0.29 | 0.05 | 1.57 |
| 31140 | Louisville/Jefferson County, KY-IN | 200 | 0.37 | 0.10 | 1.36 | 268 | 0.79 | 0.19 | 3.29 | 212 | 0.26 | 0.07 | 1.05 | 358 | 0.24 | 0.04 | 1.25 |
| 15540 | Burlington-South Burlington, VT | 201 | 0.37 | 0.08 | 1.66 | 223 | 0.82 | 0.17 | 3.92 | 134 | 0.33 | 0.07 | 1.58 | 142 | 0.30 | 0.05 | 1.64 |
| 36500 | Olympia-Tumwater, WA | 202 | 0.37 | 0.08 | 1.63 | 89 | 0.94 | 0.20 | 4.47 | 186 | 0.28 | 0.06 | 1.34 | 297 | 0.27 | 0.05 | 1.47 |
| 30700 | Lincoln, NE | 203 | 0.37 | 0.09 | 1.60 | 41 | 1.06 | 0.22 | 4.99 | 243 | 0.24 | 0.05 | 1.15 | 292 | 0.27 | 0.05 | 1.48 |
| 33540 | Missoula, MT | 204 | 0.37 | 0.08 | 1.74 | 140 | 0.84 | 0.17 | 4.06 | 233 | 0.25 | 0.05 | 1.27 | 126 | 0.30 | 0.05 | 1.65 |
| 26980 | Iowa City, IA | 205 | 0.37 | 0.08 | 1.69 | 274 | 0.79 | 0.17 | 3.76 | 185 | 0.28 | 0.06 | 1.39 | 47 | 0.40 | 0.07 | 2.18 |
| 12420 | Austin-Round Rock, TX | 206 | 0.37 | 0.11 | 1.26 | 301 | 0.77 | 0.18 | 3.28 | 70 | 0.41 | 0.11 | 1.52 | 370 | 0.21 | 0.05 | 0.92 |
| 42100 | Santa Cruz-Watsonville, CA | 207 | 0.37 | 0.08 | 1.61 | 189 | 0.83 | 0.17 | 4.00 | 151 | 0.31 | 0.06 | 1.53 | 302 | 0.27 | 0.05 | 1.39 |
| 38900 | Portland-Vancouver-Hillsboro, OR-WA | 208 | 0.37 | 0.11 | 1.23 | 134 | 0.85 | 0.19 | 3.81 | 122 | 0.35 | 0.10 | 1.24 | 56 | 0.37 | 0.08 | 1.68 |
| 43580 | Sioux City, IA-NE-SD | 209 | 0.37 | 0.08 | 1.68 | 213 | 0.82 | 0.17 | 3.93 | 264 | 0.23 | 0.05 | 1.17 | 63 | 0.36 | 0.07 | 1.91 |
| 10180 | Abilene, TX | 210 | 0.37 | 0.08 | 1.68 | 297 | 0.77 | 0.16 | 3.65 | 107 | 0.37 | 0.07 | 1.82 | 340 | 0.25 | 0.05 | 1.35 |
| 14540 | Bowling Green, KY | 211 | 0.37 | 0.08 | 1.68 | 291 | 0.78 | 0.16 | 3.68 | 139 | 0.33 | 0.07 | 1.62 | 199 | 0.29 | 0.05 | 1.61 |
| 36780 | Oshkosh-Neenah, WI | 212 | 0.37 | 0.08 | 1.68 | 66 | 0.97 | 0.20 | 4.65 | 204 | 0.27 | 0.05 | 1.32 | 186 | 0.29 | 0.05 | 1.62 |
| 48900 | Wilmington, NC | 213 | 0.37 | 0.08 | 1.60 | 58 | 0.99 | 0.22 | 4.50 | 266 | 0.23 | 0.05 | 1.12 | 263 | 0.28 | 0.05 | 1.53 |
| 39460 | Punta Gorda, FL | 214 | 0.36 | 0.08 | 1.66 | 279 | 0.79 | 0.17 | 3.76 | 144 | 0.33 | 0.07 | 1.59 | 241 | 0.29 | 0.05 | 1.57 |
| 22900 | Fort Smith, AR-OK | 215 | 0.36 | 0.08 | 1.59 | 289 | 0.78 | 0.16 | 3.70 | 95 | 0.38 | 0.08 | 1.76 | 317 | 0.26 | 0.05 | 1.44 |
| 11700 | Asheville, NC | 216 | 0.36 | 0.09 | 1.51 | 12 | 1.32 | 0.28 | 6.09 | 268 | 0.23 | 0.05 | 1.06 | 325 | 0.26 | 0.05 | 1.41 |
| 40060 | Richmond, VA | 217 | 0.36 | 0.10 | 1.32 | 329 | 0.72 | 0.19 | 2.81 | 354 | 0.17 | 0.04 | 0.75 | 235 | 0.29 | 0.05 | 1.48 |
| 35100 | New Bern, NC | 218 | 0.36 | 0.08 | 1.67 | 217 | 0.82 | 0.18 | 3.81 | 209 | 0.26 | 0.05 | 1.36 | 210 | 0.29 | 0.05 | 1.60 |
| 36100 | Ocala, FL | 219 | 0.36 | 0.08 | 1.54 | 359 | 0.66 | 0.14 | 3.03 | 223 | 0.25 | 0.05 | 1.21 | 43 | 0.42 | 0.08 | 2.22 |
| 25220 | Hammond, LA | 220 | 0.36 | 0.08 | 1.66 | 120 | 0.88 | 0.19 | 4.03 | 302 | 0.21 | 0.04 | 1.09 | 161 | 0.30 | 0.05 | 1.63 |
| 41740 | San Diego-Carlsbad, CA | 221 | 0.35 | 0.11 | 1.14 | 270 | 0.79 | 0.19 | 3.34 | 194 | 0.27 | 0.07 | 1.02 | 67 | 0.35 | 0.09 | 1.33 |
| 19180 | Danville, IL | 222 | 0.35 | 0.07 | 1.69 | 287 | 0.78 | 0.16 | 3.71 | 176 | 0.29 | 0.05 | 1.49 | 132 | 0.30 | 0.05 | 1.65 |
| 47580 | Warner Robins, GA | 223 | 0.35 | 0.08 | 1.59 | 346 | 0.69 | 0.15 | 3.12 | 158 | 0.31 | 0.06 | 1.54 | 243 | 0.28 | 0.05 | 1.56 |
| 25980 | Hinesville, GA | 224 | 0.35 | 0.07 | 1.68 | 245 | 0.81 | 0.17 | 3.76 | 242 | 0.24 | 0.05 | 1.30 | 224 | 0.29 | 0.05 | 1.59 |
| 14010 | Bloomington, IL | 225 | 0.35 | 0.08 | 1.59 | 302 | 0.77 | 0.16 | 3.63 | 206 | 0.27 | 0.05 | 1.31 | 212 | 0.29 | 0.05 | 1.60 |
| 31540 | Madison, WI | 226 | 0.35 | 0.09 | 1.40 | 88 | 0.94 | 0.20 | 4.36 | 255 | 0.24 | 0.05 | 1.04 | 36 | 0.44 | 0.08 | 2.32 |
| 18020 | Columbus, IN | 227 | 0.35 | 0.07 | 1.67 | 171 | 0.84 | 0.17 | 4.03 | 182 | 0.28 | 0.05 | 1.45 | 159 | 0.30 | 0.05 | 1.63 |
| 30140 | Lebanon, PA | 228 | 0.35 | 0.07 | 1.62 | 191 | 0.83 | 0.17 | 3.98 | 250 | 0.24 | 0.05 | 1.22 | 271 | 0.28 | 0.05 | 1.52 |
| 15180 | Brownsville-Harlingen, TX | 229 | 0.35 | 0.08 | 1.46 | 175 | 0.84 | 0.17 | 4.02 | 251 | 0.24 | 0.04 | 1.28 | 299 | 0.27 | 0.06 | 1.23 |
| 14100 | Bloomsburg-Berwick, PA | 230 | 0.34 | 0.07 | 1.65 | 174 | 0.84 | 0.17 | 4.02 | 198 | 0.27 | 0.05 | 1.39 | 108 | 0.30 | 0.05 | 1.67 |
| 26820 | Idaho Falls, ID | 231 | 0.34 | 0.07 | 1.60 | 145 | 0.84 | 0.17 | 4.05 | 171 | 0.29 | 0.06 | 1.47 | 273 | 0.28 | 0.05 | 1.52 |
| 47260 | Virginia Beach-Norfolk-Newport News, VA-NC | 232 | 0.34 | 0.10 | 1.20 | 315 | 0.75 | 0.20 | 2.78 | 364 | 0.16 | 0.04 | 0.67 | 371 | 0.20 | 0.04 | 1.04 |
| 16740 | Charlotte-Concord-Gastonia, NC-SC | 233 | 0.34 | 0.10 | 1.15 | 350 | 0.67 | 0.18 | 2.52 | 249 | 0.24 | 0.06 | 0.92 | 364 | 0.22 | 0.05 | 1.09 |
| 12020 | Athens-Clarke County, GA | 234 | 0.34 | 0.07 | 1.54 | 127 | 0.86 | 0.19 | 3.95 | 340 | 0.18 | 0.04 | 0.93 | 272 | 0.28 | 0.05 | 1.52 |
| 20500 | Durham-Chapel Hill, NC | 235 | 0.34 | 0.08 | 1.39 | 117 | 0.89 | 0.21 | 3.72 | 371 | 0.14 | 0.03 | 0.67 | 52 | 0.38 | 0.07 | 1.97 |
| 42680 | Sebastian-Vero Beach, FL | 236 | 0.34 | 0.07 | 1.57 | 106 | 0.90 | 0.19 | 4.25 | 245 | 0.24 | 0.05 | 1.23 | 277 | 0.28 | 0.05 | 1.51 |
| 41940 | San Jose-Sunnyvale-Santa Clara, CA | 237 | 0.34 | 0.10 | 1.17 | 83 | 0.95 | 0.21 | 4.30 | 128 | 0.34 | 0.08 | 1.41 | 27 | 0.46 | 0.11 | 1.88 |
| 29340 | Lake Charles, LA | 238 | 0.34 | 0.07 | 1.53 | 364 | 0.64 | 0.14 | 2.93 | 192 | 0.27 | 0.06 | 1.36 | 190 | 0.29 | 0.05 | 1.62 |
| 22500 | Florence, SC | 239 | 0.34 | 0.07 | 1.53 | 343 | 0.69 | 0.16 | 3.09 | 241 | 0.24 | 0.05 | 1.24 | 147 | 0.30 | 0.05 | 1.64 |
| 17660 | Coeur d'Alene, ID | 240 | 0.34 | 0.07 | 1.56 | 143 | 0.84 | 0.17 | 4.06 | 187 | 0.28 | 0.06 | 1.38 | 182 | 0.29 | 0.05 | 1.62 |
| 27620 | Jefferson City, MO | 241 | 0.34 | 0.07 | 1.56 | 290 | 0.78 | 0.16 | 3.70 | 267 | 0.23 | 0.05 | 1.16 | 133 | 0.30 | 0.05 | 1.65 |
| 33340 | Milwaukee-Waukesha-West Allis, WI | 242 | 0.33 | 0.09 | 1.20 | 42 | 1.04 | 0.27 | 4.04 | 367 | 0.15 | 0.03 | 0.62 | 313 | 0.27 | 0.05 | 1.32 |
| 11180 | Ames, IA | 243 | 0.33 | 0.07 | 1.59 | 182 | 0.83 | 0.17 | 4.01 | 203 | 0.27 | 0.05 | 1.38 | 122 | 0.30 | 0.05 | 1.66 |
| 16540 | Chambersburg-Waynesboro, PA | 244 | 0.33 | 0.07 | 1.55 | 241 | 0.81 | 0.17 | 3.87 | 344 | 0.18 | 0.04 | 0.93 | 207 | 0.29 | 0.05 | 1.60 |
| 40660 | Rome, GA | 245 | 0.33 | 0.07 | 1.59 | 110 | 0.89 | 0.19 | 4.22 | 286 | 0.22 | 0.04 | 1.14 | 227 | 0.29 | 0.05 | 1.58 |
| 20220 | Dubuque, IA | 246 | 0.33 | 0.07 | 1.59 | 196 | 0.83 | 0.17 | 3.98 | 215 | 0.26 | 0.05 | 1.34 | 110 | 0.30 | 0.05 | 1.67 |
| 13380 | Bellingham, WA | 247 | 0.33 | 0.07 | 1.50 | 22 | 1.16 | 0.24 | 5.59 | 357 | 0.17 | 0.03 | 0.86 | 55 | 0.37 | 0.07 | 2.01 |
| 41100 | St. George, UT | 248 | 0.33 | 0.07 | 1.54 | 159 | 0.84 | 0.17 | 4.04 | 270 | 0.23 | 0.05 | 1.16 | 262 | 0.28 | 0.05 | 1.53 |
| 42700 | Sebring, FL | 249 | 0.33 | 0.07 | 1.57 | 275 | 0.79 | 0.17 | 3.76 | 184 | 0.28 | 0.05 | 1.45 | 281 | 0.28 | 0.05 | 1.50 |
| 41540 | Salisbury, MD-DE | 250 | 0.33 | 0.08 | 1.41 | 43 | 1.04 | 0.24 | 4.56 | 356 | 0.17 | 0.03 | 0.83 | 320 | 0.26 | 0.05 | 1.43 |
| 27100 | Jackson, MI | 251 | 0.33 | 0.07 | 1.52 | 296 | 0.77 | 0.16 | 3.65 | 274 | 0.23 | 0.05 | 1.15 | 164 | 0.30 | 0.05 | 1.63 |
| 23900 | Gettysburg, PA | 252 | 0.33 | 0.07 | 1.56 | 170 | 0.84 | 0.17 | 4.03 | 218 | 0.26 | 0.05 | 1.32 | 192 | 0.29 | 0.05 | 1.62 |
| 22540 | Fond du Lac, WI | 253 | 0.33 | 0.07 | 1.56 | 166 | 0.84 | 0.17 | 4.03 | 219 | 0.26 | 0.05 | 1.32 | 157 | 0.30 | 0.05 | 1.64 |
| 10580 | Albany-Schenectady-Troy, NY | 254 | 0.33 | 0.08 | 1.27 | 76 | 0.96 | 0.22 | 4.26 | 225 | 0.25 | 0.06 | 1.07 | 80 | 0.33 | 0.06 | 1.73 |
| 15980 | Cape Coral-Fort Myers, FL | 255 | 0.33 | 0.08 | 1.31 | 280 | 0.79 | 0.17 | 3.56 | 131 | 0.34 | 0.08 | 1.45 | 377 | 0.19 | 0.04 | 0.95 |
| 20260 | Duluth, MN-WI | 256 | 0.33 | 0.07 | 1.45 | 218 | 0.82 | 0.17 | 3.93 | 173 | 0.29 | 0.06 | 1.36 | 143 | 0.30 | 0.05 | 1.64 |
| 30620 | Lima, OH | 257 | 0.33 | 0.07 | 1.55 | 294 | 0.77 | 0.16 | 3.68 | 210 | 0.26 | 0.05 | 1.36 | 119 | 0.30 | 0.05 | 1.66 |
| 38860 | Portland-South Portland, ME | 258 | 0.32 | 0.08 | 1.35 | 97 | 0.92 | 0.19 | 4.37 | 188 | 0.28 | 0.06 | 1.21 | 222 | 0.29 | 0.05 | 1.59 |
| 14020 | Bloomington, IN | 259 | 0.32 | 0.07 | 1.50 | 71 | 0.96 | 0.20 | 4.61 | 278 | 0.22 | 0.04 | 1.12 | 156 | 0.30 | 0.05 | 1.64 |
| 30340 | Lewiston-Auburn, ME | 260 | 0.32 | 0.07 | 1.54 | 179 | 0.83 | 0.17 | 4.01 | 316 | 0.20 | 0.04 | 1.03 | 100 | 0.30 | 0.05 | 1.67 |
| 44940 | Sumter, SC | 261 | 0.32 | 0.07 | 1.54 | 330 | 0.72 | 0.16 | 3.30 | 263 | 0.23 | 0.04 | 1.23 | 136 | 0.30 | 0.05 | 1.65 |
| 29460 | Lakeland-Winter Haven, FL | 262 | 0.32 | 0.08 | 1.31 | 375 | 0.53 | 0.12 | 2.39 | 89 | 0.39 | 0.09 | 1.67 | 357 | 0.24 | 0.05 | 1.20 |
| 20700 | East Stroudsburg, PA | 263 | 0.32 | 0.07 | 1.49 | 326 | 0.73 | 0.16 | 3.43 | 168 | 0.30 | 0.06 | 1.50 | 308 | 0.27 | 0.05 | 1.45 |
| 22420 | Flint, MI | 264 | 0.32 | 0.08 | 1.37 | 38 | 1.07 | 0.25 | 4.66 | 370 | 0.14 | 0.03 | 0.68 | 253 | 0.28 | 0.05 | 1.55 |
| 16820 | Charlottesville, VA | 265 | 0.32 | 0.07 | 1.45 | 99 | 0.92 | 0.20 | 4.23 | 301 | 0.21 | 0.04 | 1.04 | 233 | 0.29 | 0.05 | 1.57 |
| 31420 | Macon, GA | 266 | 0.32 | 0.07 | 1.45 | 360 | 0.65 | 0.15 | 2.88 | 248 | 0.24 | 0.05 | 1.22 | 175 | 0.29 | 0.05 | 1.63 |
| 29540 | Lancaster, PA | 267 | 0.32 | 0.08 | 1.33 | 130 | 0.86 | 0.18 | 4.04 | 296 | 0.21 | 0.05 | 0.96 | 89 | 0.31 | 0.06 | 1.63 |
| 45460 | Terre Haute, IN | 268 | 0.32 | 0.07 | 1.48 | 257 | 0.80 | 0.17 | 3.82 | 205 | 0.27 | 0.05 | 1.31 | 130 | 0.30 | 0.05 | 1.65 |
| 39740 | Reading, PA | 269 | 0.32 | 0.07 | 1.36 | 121 | 0.87 | 0.19 | 4.12 | 323 | 0.19 | 0.04 | 0.93 | 270 | 0.28 | 0.05 | 1.44 |
| 22020 | Fargo, ND-MN | 270 | 0.32 | 0.07 | 1.44 | 269 | 0.79 | 0.17 | 3.78 | 258 | 0.23 | 0.05 | 1.14 | 191 | 0.29 | 0.05 | 1.62 |
| 11540 | Appleton, WI | 271 | 0.32 | 0.07 | 1.44 | 24 | 1.16 | 0.24 | 5.54 | 324 | 0.19 | 0.04 | 0.96 | 223 | 0.29 | 0.05 | 1.59 |
| 43100 | Sheboygan, WI | 272 | 0.32 | 0.07 | 1.50 | 183 | 0.83 | 0.17 | 4.01 | 230 | 0.25 | 0.05 | 1.29 | 196 | 0.29 | 0.05 | 1.61 |
| 48700 | Williamsport, PA | 273 | 0.32 | 0.07 | 1.50 | 81 | 0.95 | 0.20 | 4.56 | 322 | 0.20 | 0.04 | 1.01 | 109 | 0.30 | 0.05 | 1.67 |
| 15260 | Brunswick, GA | 274 | 0.32 | 0.07 | 1.50 | 214 | 0.82 | 0.18 | 3.82 | 294 | 0.21 | 0.04 | 1.10 | 179 | 0.29 | 0.05 | 1.62 |
| 24780 | Greenville, NC | 275 | 0.32 | 0.07 | 1.46 | 345 | 0.69 | 0.15 | 3.12 | 227 | 0.25 | 0.05 | 1.29 | 229 | 0.29 | 0.05 | 1.58 |
| 49660 | Youngstown-Warren-Boardman, OH-PA | 276 | 0.32 | 0.08 | 1.31 | 278 | 0.79 | 0.17 | 3.57 | 262 | 0.23 | 0.05 | 1.04 | 278 | 0.28 | 0.05 | 1.51 |
| 17460 | Cleveland-Elyria, OH | 277 | 0.32 | 0.09 | 1.10 | 312 | 0.76 | 0.20 | 2.89 | 328 | 0.19 | 0.05 | 0.76 | 373 | 0.20 | 0.04 | 1.03 |
| 45540 | The Villages, FL | 278 | 0.32 | 0.07 | 1.49 | 265 | 0.79 | 0.17 | 3.79 | 317 | 0.20 | 0.04 | 1.03 | 185 | 0.29 | 0.05 | 1.62 |
| 46220 | Tuscaloosa, AL | 279 | 0.31 | 0.07 | 1.42 | 336 | 0.71 | 0.16 | 3.16 | 281 | 0.22 | 0.04 | 1.12 | 209 | 0.29 | 0.05 | 1.60 |
| 21660 | Eugene, OR | 280 | 0.31 | 0.07 | 1.36 | 209 | 0.82 | 0.17 | 3.94 | 160 | 0.30 | 0.07 | 1.38 | 327 | 0.26 | 0.05 | 1.41 |
| 36740 | Orlando-Kissimmee-Sanford, FL | 281 | 0.31 | 0.09 | 1.07 | 342 | 0.70 | 0.18 | 2.72 | 175 | 0.29 | 0.07 | 1.11 | 378 | 0.18 | 0.04 | 0.80 |
| 44420 | Staunton-Waynesboro, VA | 282 | 0.31 | 0.07 | 1.48 | 263 | 0.80 | 0.17 | 3.79 | 235 | 0.25 | 0.05 | 1.26 | 139 | 0.30 | 0.05 | 1.65 |
| 17140 | Cincinnati, OH-KY-IN | 283 | 0.31 | 0.09 | 1.08 | 198 | 0.83 | 0.21 | 3.29 | 277 | 0.22 | 0.06 | 0.85 | 152 | 0.30 | 0.06 | 1.55 |
| 19780 | Des Moines-West Des Moines, IA | 284 | 0.31 | 0.08 | 1.27 | 255 | 0.80 | 0.17 | 3.72 | 197 | 0.27 | 0.06 | 1.17 | 348 | 0.25 | 0.05 | 1.32 |
| 30300 | Lewiston, ID-WA | 285 | 0.31 | 0.06 | 1.51 | 138 | 0.84 | 0.18 | 4.07 | 276 | 0.23 | 0.04 | 1.20 | 112 | 0.30 | 0.05 | 1.67 |
| 33860 | Montgomery, AL | 286 | 0.31 | 0.07 | 1.34 | 373 | 0.54 | 0.12 | 2.33 | 310 | 0.20 | 0.04 | 1.01 | 237 | 0.29 | 0.05 | 1.57 |
| 47460 | Walla Walla, WA | 287 | 0.31 | 0.06 | 1.51 | 150 | 0.84 | 0.17 | 4.05 | 261 | 0.23 | 0.04 | 1.24 | 248 | 0.28 | 0.05 | 1.55 |
| 24580 | Green Bay, WI | 288 | 0.31 | 0.07 | 1.35 | 240 | 0.81 | 0.17 | 3.87 | 178 | 0.28 | 0.06 | 1.32 | 296 | 0.27 | 0.05 | 1.47 |
| 38340 | Pittsfield, MA | 289 | 0.31 | 0.07 | 1.45 | 204 | 0.82 | 0.17 | 3.96 | 247 | 0.24 | 0.05 | 1.22 | 166 | 0.30 | 0.05 | 1.63 |
| 19340 | Davenport-Moline-Rock Island, IA-IL | 290 | 0.31 | 0.07 | 1.32 | 235 | 0.81 | 0.17 | 3.77 | 211 | 0.26 | 0.06 | 1.22 | 333 | 0.26 | 0.05 | 1.40 |
| 43780 | South Bend-Mishawaka, IN-MI | 291 | 0.31 | 0.07 | 1.34 | 295 | 0.77 | 0.17 | 3.57 | 288 | 0.22 | 0.04 | 1.05 | 303 | 0.27 | 0.05 | 1.46 |
| 32580 | McAllen-Edinburg-Mission, TX | 292 | 0.30 | 0.08 | 1.20 | 207 | 0.82 | 0.17 | 3.95 | 170 | 0.29 | 0.06 | 1.54 | 259 | 0.28 | 0.07 | 1.16 |
| 38300 | Pittsburgh, PA | 293 | 0.30 | 0.09 | 1.05 | 190 | 0.83 | 0.20 | 3.42 | 282 | 0.22 | 0.06 | 0.82 | 339 | 0.25 | 0.05 | 1.36 |
| 25500 | Harrisonburg, VA | 294 | 0.30 | 0.06 | 1.43 | 230 | 0.81 | 0.17 | 3.90 | 237 | 0.25 | 0.05 | 1.26 | 260 | 0.28 | 0.05 | 1.54 |
| 41060 | St. Cloud, MN | 295 | 0.30 | 0.07 | 1.39 | 95 | 0.93 | 0.20 | 4.41 | 304 | 0.21 | 0.04 | 1.04 | 171 | 0.29 | 0.05 | 1.63 |
| 14740 | Bremerton-Silverdale, WA | 296 | 0.30 | 0.07 | 1.36 | 85 | 0.95 | 0.20 | 4.52 | 256 | 0.24 | 0.05 | 1.15 | 282 | 0.28 | 0.05 | 1.50 |
| 37100 | Oxnard-Thousand Oaks-Ventura, CA | 297 | 0.30 | 0.08 | 1.19 | 107 | 0.90 | 0.19 | 4.24 | 174 | 0.29 | 0.06 | 1.29 | 367 | 0.21 | 0.05 | 1.00 |
| 12980 | Battle Creek, MI | 298 | 0.30 | 0.06 | 1.42 | 306 | 0.77 | 0.16 | 3.62 | 236 | 0.25 | 0.05 | 1.26 | 189 | 0.29 | 0.05 | 1.62 |
| 44220 | Springfield, OH | 299 | 0.30 | 0.06 | 1.42 | 286 | 0.78 | 0.16 | 3.71 | 327 | 0.19 | 0.04 | 0.98 | 141 | 0.30 | 0.05 | 1.64 |
| 29100 | La Crosse-Onalaska, WI-MN | 300 | 0.30 | 0.06 | 1.41 | 178 | 0.83 | 0.17 | 4.01 | 260 | 0.23 | 0.05 | 1.18 | 111 | 0.30 | 0.05 | 1.67 |
| 25940 | Hilton Head Island-Bluffton-Beaufort, SC | 301 | 0.30 | 0.06 | 1.36 | 320 | 0.74 | 0.16 | 3.41 | 336 | 0.18 | 0.04 | 0.94 | 61 | 0.36 | 0.07 | 1.95 |
| 29620 | Lansing-East Lansing, MI | 302 | 0.30 | 0.07 | 1.25 | 305 | 0.77 | 0.17 | 3.53 | 254 | 0.24 | 0.05 | 1.09 | 69 | 0.35 | 0.06 | 1.86 |
| 48540 | Wheeling, WV-OH | 303 | 0.30 | 0.06 | 1.39 | 229 | 0.81 | 0.17 | 3.90 | 272 | 0.23 | 0.05 | 1.15 | 94 | 0.30 | 0.05 | 1.68 |
| 40420 | Rockford, IL | 304 | 0.29 | 0.07 | 1.29 | 118 | 0.89 | 0.19 | 4.06 | 287 | 0.22 | 0.04 | 1.05 | 350 | 0.24 | 0.05 | 1.31 |
| 44100 | Springfield, IL | 305 | 0.29 | 0.06 | 1.35 | 333 | 0.72 | 0.15 | 3.36 | 228 | 0.25 | 0.05 | 1.24 | 145 | 0.30 | 0.05 | 1.64 |
| 21500 | Erie, PA | 306 | 0.29 | 0.07 | 1.31 | 49 | 1.00 | 0.21 | 4.68 | 337 | 0.18 | 0.04 | 0.90 | 231 | 0.29 | 0.05 | 1.57 |
| 33220 | Midland, MI | 307 | 0.29 | 0.06 | 1.41 | 165 | 0.84 | 0.17 | 4.03 | 292 | 0.21 | 0.04 | 1.11 | 107 | 0.30 | 0.05 | 1.67 |
| 24260 | Grand Island, NE | 308 | 0.29 | 0.06 | 1.41 | 172 | 0.84 | 0.17 | 4.02 | 284 | 0.22 | 0.04 | 1.16 | 274 | 0.28 | 0.05 | 1.52 |
| 19460 | Decatur, AL | 309 | 0.29 | 0.06 | 1.36 | 318 | 0.75 | 0.16 | 3.51 | 253 | 0.24 | 0.05 | 1.21 | 226 | 0.29 | 0.05 | 1.58 |
| 18700 | Corvallis, OR | 310 | 0.29 | 0.06 | 1.39 | 151 | 0.84 | 0.17 | 4.04 | 290 | 0.21 | 0.04 | 1.12 | 180 | 0.29 | 0.05 | 1.62 |
| 24340 | Grand Rapids-Wyoming, MI | 311 | 0.29 | 0.07 | 1.12 | 77 | 0.96 | 0.22 | 4.26 | 275 | 0.23 | 0.05 | 0.95 | 319 | 0.26 | 0.05 | 1.36 |
| 46540 | Utica-Rome, NY | 312 | 0.28 | 0.06 | 1.27 | 307 | 0.76 | 0.16 | 3.62 | 291 | 0.21 | 0.04 | 1.03 | 256 | 0.28 | 0.05 | 1.55 |
| 36140 | Ocean City, NJ | 313 | 0.28 | 0.06 | 1.37 | 219 | 0.82 | 0.17 | 3.92 | 300 | 0.21 | 0.04 | 1.09 | 195 | 0.29 | 0.05 | 1.62 |
| 27500 | Janesville-Beloit, WI | 314 | 0.28 | 0.06 | 1.33 | 86 | 0.94 | 0.20 | 4.50 | 342 | 0.18 | 0.04 | 0.93 | 252 | 0.28 | 0.05 | 1.55 |
| 27980 | Kahului-Wailuku-Lahaina, HI | 315 | 0.28 | 0.06 | 1.32 | 53 | 0.99 | 0.21 | 4.79 | 265 | 0.23 | 0.04 | 1.22 | 279 | 0.28 | 0.05 | 1.51 |
| 31740 | Manhattan, KS | 316 | 0.28 | 0.06 | 1.35 | 228 | 0.81 | 0.17 | 3.90 | 295 | 0.21 | 0.04 | 1.10 | 201 | 0.29 | 0.05 | 1.61 |
| 22220 | Fayetteville-Springdale-Rogers, AR-MO | 317 | 0.28 | 0.07 | 1.18 | 104 | 0.91 | 0.19 | 4.29 | 213 | 0.26 | 0.06 | 1.18 | 366 | 0.22 | 0.04 | 1.14 |
| 47940 | Waterloo-Cedar Falls, IA | 318 | 0.28 | 0.06 | 1.30 | 292 | 0.78 | 0.16 | 3.68 | 283 | 0.22 | 0.04 | 1.11 | 177 | 0.29 | 0.05 | 1.62 |
| 27060 | Ithaca, NY | 319 | 0.28 | 0.06 | 1.33 | 210 | 0.82 | 0.17 | 3.94 | 299 | 0.21 | 0.04 | 1.09 | 154 | 0.30 | 0.05 | 1.64 |
| 19500 | Decatur, IL | 320 | 0.28 | 0.06 | 1.32 | 308 | 0.76 | 0.16 | 3.60 | 303 | 0.21 | 0.04 | 1.08 | 113 | 0.30 | 0.05 | 1.67 |
| 45780 | Toledo, OH | 321 | 0.27 | 0.07 | 1.14 | 341 | 0.70 | 0.16 | 3.11 | 259 | 0.23 | 0.05 | 1.05 | 342 | 0.25 | 0.05 | 1.35 |
| 17780 | College Station-Bryan, TX | 322 | 0.27 | 0.06 | 1.24 | 224 | 0.82 | 0.18 | 3.81 | 349 | 0.18 | 0.04 | 0.91 | 99 | 0.30 | 0.06 | 1.58 |
| 28740 | Kingston, NY | 323 | 0.27 | 0.06 | 1.27 | 276 | 0.79 | 0.17 | 3.76 | 280 | 0.22 | 0.04 | 1.12 | 276 | 0.28 | 0.05 | 1.51 |
| 43620 | Sioux Falls, SD | 324 | 0.27 | 0.06 | 1.24 | 277 | 0.79 | 0.17 | 3.76 | 271 | 0.23 | 0.05 | 1.11 | 225 | 0.29 | 0.05 | 1.58 |
| 28100 | Kankakee, IL | 325 | 0.27 | 0.06 | 1.31 | 313 | 0.75 | 0.16 | 3.56 | 297 | 0.21 | 0.04 | 1.09 | 230 | 0.29 | 0.05 | 1.57 |
| 33140 | Michigan City-La Porte, IN | 326 | 0.27 | 0.06 | 1.31 | 293 | 0.78 | 0.16 | 3.68 | 306 | 0.20 | 0.04 | 1.06 | 193 | 0.29 | 0.05 | 1.62 |
| 15680 | California-Lexington Park, MD | 327 | 0.27 | 0.06 | 1.30 | 310 | 0.76 | 0.16 | 3.58 | 305 | 0.21 | 0.04 | 1.08 | 163 | 0.30 | 0.05 | 1.63 |
| 15940 | Canton-Massillon, OH | 328 | 0.27 | 0.06 | 1.17 | 335 | 0.71 | 0.15 | 3.32 | 298 | 0.21 | 0.04 | 0.98 | 204 | 0.29 | 0.05 | 1.61 |
| 48060 | Watertown-Fort Drum, NY | 329 | 0.27 | 0.06 | 1.29 | 247 | 0.81 | 0.17 | 3.85 | 312 | 0.20 | 0.04 | 1.04 | 211 | 0.29 | 0.05 | 1.60 |
| 29940 | Lawrence, KS | 330 | 0.27 | 0.06 | 1.28 | 226 | 0.82 | 0.17 | 3.91 | 311 | 0.20 | 0.04 | 1.05 | 198 | 0.29 | 0.05 | 1.61 |
| 16980 | Chicago-Naperville-Elgin, IL-IN-WI | 331 | 0.27 | 0.09 | 0.78 | 100 | 0.92 | 0.29 | 2.86 | 379 | 0.10 | 0.03 | 0.38 | 372 | 0.20 | 0.05 | 0.75 |
| 28020 | Kalamazoo-Portage, MI | 332 | 0.27 | 0.06 | 1.19 | 340 | 0.70 | 0.15 | 3.26 | 240 | 0.24 | 0.05 | 1.16 | 289 | 0.27 | 0.05 | 1.49 |
| 42540 | Scranton--Wilkes-Barre--Hazleton, PA | 333 | 0.27 | 0.06 | 1.12 | 314 | 0.75 | 0.16 | 3.55 | 350 | 0.18 | 0.04 | 0.81 | 85 | 0.32 | 0.06 | 1.69 |
| 10540 | Albany, OR | 334 | 0.27 | 0.06 | 1.27 | 141 | 0.84 | 0.17 | 4.06 | 320 | 0.20 | 0.04 | 1.01 | 228 | 0.29 | 0.05 | 1.58 |
| 23580 | Gainesville, GA | 335 | 0.27 | 0.06 | 1.23 | 303 | 0.77 | 0.16 | 3.63 | 332 | 0.19 | 0.04 | 0.97 | 88 | 0.31 | 0.06 | 1.63 |
| 34580 | Mount Vernon-Anacortes, WA | 336 | 0.27 | 0.06 | 1.27 | 153 | 0.84 | 0.17 | 4.04 | 309 | 0.20 | 0.04 | 1.05 | 293 | 0.27 | 0.05 | 1.48 |
| 16300 | Cedar Rapids, IA | 337 | 0.27 | 0.06 | 1.20 | 103 | 0.91 | 0.19 | 4.32 | 338 | 0.18 | 0.04 | 0.90 | 202 | 0.29 | 0.05 | 1.61 |
| 34980 | Nashville-Davidson--Murfreesboro--Franklin, TN | 338 | 0.27 | 0.07 | 0.96 | 374 | 0.54 | 0.13 | 2.22 | 307 | 0.20 | 0.05 | 0.81 | 153 | 0.30 | 0.06 | 1.48 |
| 40980 | Saginaw, MI | 339 | 0.27 | 0.06 | 1.23 | 347 | 0.68 | 0.15 | 3.16 | 273 | 0.23 | 0.05 | 1.15 | 266 | 0.28 | 0.05 | 1.53 |
| 39540 | Racine, WI | 340 | 0.27 | 0.06 | 1.23 | 323 | 0.73 | 0.16 | 3.46 | 279 | 0.22 | 0.04 | 1.12 | 307 | 0.27 | 0.05 | 1.45 |
| 24140 | Goldsboro, NC | 341 | 0.26 | 0.06 | 1.26 | 352 | 0.67 | 0.14 | 3.12 | 285 | 0.22 | 0.04 | 1.14 | 258 | 0.28 | 0.05 | 1.54 |
| 11020 | Altoona, PA | 342 | 0.26 | 0.06 | 1.26 | 185 | 0.83 | 0.17 | 4.01 | 330 | 0.19 | 0.04 | 0.97 | 95 | 0.30 | 0.05 | 1.68 |
| 43300 | Sherman-Denison, TX | 343 | 0.26 | 0.06 | 1.25 | 254 | 0.80 | 0.17 | 3.83 | 313 | 0.20 | 0.04 | 1.04 | 264 | 0.28 | 0.05 | 1.53 |
| 24020 | Glens Falls, NY | 344 | 0.26 | 0.06 | 1.25 | 193 | 0.83 | 0.17 | 3.98 | 331 | 0.19 | 0.04 | 0.97 | 123 | 0.30 | 0.05 | 1.66 |
| 42020 | San Luis Obispo-Paso Robles-Arroyo Grande, CA | 345 | 0.26 | 0.06 | 1.17 | 231 | 0.81 | 0.17 | 3.90 | 318 | 0.20 | 0.04 | 0.98 | 118 | 0.30 | 0.06 | 1.57 |
| 34940 | Naples-Immokalee-Marco Island, FL | 346 | 0.26 | 0.06 | 1.14 | 332 | 0.72 | 0.15 | 3.37 | 333 | 0.19 | 0.04 | 0.92 | 322 | 0.26 | 0.05 | 1.36 |
| 33460 | Minneapolis-St. Paul-Bloomington, MN-WI | 347 | 0.26 | 0.08 | 0.86 | 317 | 0.75 | 0.19 | 2.98 | 351 | 0.17 | 0.05 | 0.63 | 351 | 0.24 | 0.05 | 1.19 |
| 11460 | Ann Arbor, MI | 348 | 0.26 | 0.06 | 1.14 | 67 | 0.97 | 0.21 | 4.41 | 366 | 0.15 | 0.03 | 0.72 | 268 | 0.28 | 0.05 | 1.52 |
| 40340 | Rochester, MN | 349 | 0.26 | 0.06 | 1.18 | 267 | 0.79 | 0.17 | 3.78 | 308 | 0.20 | 0.04 | 1.01 | 220 | 0.29 | 0.05 | 1.59 |
| 46520 | Urban Honolulu, HI | 350 | 0.25 | 0.06 | 1.01 | 325 | 0.73 | 0.16 | 3.44 | 239 | 0.24 | 0.05 | 1.20 | 316 | 0.27 | 0.05 | 1.36 |
| 46340 | Tyler, TX | 351 | 0.25 | 0.05 | 1.16 | 355 | 0.67 | 0.14 | 3.09 | 341 | 0.18 | 0.04 | 0.93 | 343 | 0.25 | 0.05 | 1.33 |
| 37900 | Peoria, IL | 352 | 0.25 | 0.06 | 1.11 | 282 | 0.78 | 0.17 | 3.63 | 326 | 0.19 | 0.04 | 0.91 | 246 | 0.28 | 0.05 | 1.56 |
| 33780 | Monroe, MI | 353 | 0.25 | 0.05 | 1.19 | 206 | 0.82 | 0.17 | 3.95 | 345 | 0.18 | 0.04 | 0.93 | 158 | 0.30 | 0.05 | 1.63 |
| 37980 | Philadelphia-Camden-Wilmington, PA-NJ-DE-MD | 354 | 0.25 | 0.08 | 0.78 | 372 | 0.55 | 0.16 | 1.91 | 376 | 0.12 | 0.03 | 0.45 | 379 | 0.17 | 0.04 | 0.76 |
| 15500 | Burlington, NC | 355 | 0.25 | 0.05 | 1.16 | 339 | 0.70 | 0.15 | 3.26 | 319 | 0.20 | 0.04 | 1.02 | 285 | 0.27 | 0.05 | 1.50 |
| 21780 | Evansville, IN-KY | 356 | 0.25 | 0.05 | 1.10 | 135 | 0.85 | 0.18 | 3.98 | 352 | 0.17 | 0.03 | 0.83 | 197 | 0.29 | 0.05 | 1.61 |
| 16580 | Champaign-Urbana, IL | 357 | 0.25 | 0.05 | 1.13 | 221 | 0.82 | 0.18 | 3.81 | 358 | 0.17 | 0.03 | 0.86 | 244 | 0.28 | 0.05 | 1.56 |
| 44300 | State College, PA | 358 | 0.25 | 0.05 | 1.16 | 242 | 0.81 | 0.17 | 3.87 | 346 | 0.18 | 0.04 | 0.92 | 146 | 0.30 | 0.05 | 1.64 |
| 47900 | Washington-Arlington-Alexandria, DC-VA-MD-WV | 359 | 0.25 | 0.08 | 0.76 | 371 | 0.55 | 0.17 | 1.86 | 377 | 0.12 | 0.03 | 0.47 | 380 | 0.15 | 0.03 | 0.64 |
| 13780 | Binghamton, NY | 360 | 0.24 | 0.05 | 1.11 | 281 | 0.79 | 0.17 | 3.74 | 325 | 0.19 | 0.04 | 0.95 | 216 | 0.29 | 0.05 | 1.60 |
| 31700 | Manchester-Nashua, NH | 361 | 0.24 | 0.06 | 1.06 | 266 | 0.79 | 0.17 | 3.79 | 347 | 0.18 | 0.04 | 0.86 | 310 | 0.27 | 0.05 | 1.45 |
| 34740 | Muskegon, MI | 362 | 0.24 | 0.05 | 1.13 | 328 | 0.72 | 0.15 | 3.40 | 335 | 0.18 | 0.04 | 0.94 | 221 | 0.29 | 0.05 | 1.59 |
| 30460 | Lexington-Fayette, KY | 363 | 0.24 | 0.06 | 1.03 | 367 | 0.62 | 0.14 | 2.86 | 353 | 0.17 | 0.04 | 0.81 | 68 | 0.35 | 0.07 | 1.87 |
| 27340 | Jacksonville, NC | 364 | 0.23 | 0.05 | 1.08 | 334 | 0.71 | 0.15 | 3.33 | 334 | 0.18 | 0.04 | 0.95 | 300 | 0.27 | 0.05 | 1.46 |
| 44140 | Springfield, MA | 365 | 0.23 | 0.05 | 0.96 | 128 | 0.86 | 0.19 | 3.94 | 362 | 0.16 | 0.03 | 0.75 | 374 | 0.20 | 0.04 | 1.01 |
| 19820 | Detroit-Warren-Dearborn, MI | 366 | 0.22 | 0.07 | 0.74 | 382 | 0.43 | 0.12 | 1.59 | 378 | 0.11 | 0.03 | 0.44 | 338 | 0.25 | 0.05 | 1.25 |
| 12700 | Barnstable Town, MA | 367 | 0.22 | 0.05 | 1.04 | 236 | 0.81 | 0.17 | 3.88 | 361 | 0.16 | 0.03 | 0.82 | 173 | 0.29 | 0.05 | 1.63 |
| 29200 | Lafayette-West Lafayette, IN | 368 | 0.22 | 0.05 | 1.04 | 271 | 0.79 | 0.17 | 3.78 | 359 | 0.17 | 0.03 | 0.86 | 267 | 0.28 | 0.05 | 1.52 |
| 45060 | Syracuse, NY | 369 | 0.22 | 0.05 | 0.93 | 101 | 0.91 | 0.20 | 4.12 | 368 | 0.14 | 0.03 | 0.66 | 315 | 0.27 | 0.05 | 1.44 |
| 24660 | Greensboro-High Point, NC | 370 | 0.22 | 0.05 | 0.92 | 379 | 0.50 | 0.12 | 2.13 | 339 | 0.18 | 0.04 | 0.85 | 359 | 0.23 | 0.04 | 1.23 |
| 14860 | Bridgeport-Stamford-Norwalk, CT | 371 | 0.22 | 0.05 | 0.88 | 357 | 0.66 | 0.15 | 2.92 | 365 | 0.15 | 0.03 | 0.70 | 368 | 0.21 | 0.04 | 1.05 |
| 35300 | New Haven-Milford, CT | 372 | 0.22 | 0.05 | 0.89 | 370 | 0.58 | 0.13 | 2.57 | 369 | 0.14 | 0.03 | 0.65 | 362 | 0.23 | 0.05 | 1.15 |
| 39580 | Raleigh, NC | 373 | 0.21 | 0.05 | 0.83 | 381 | 0.48 | 0.11 | 2.04 | 289 | 0.22 | 0.05 | 0.91 | 376 | 0.19 | 0.04 | 0.98 |
| 25540 | Hartford-West Hartford-East Hartford, CT | 374 | 0.21 | 0.05 | 0.82 | 369 | 0.60 | 0.14 | 2.63 | 363 | 0.16 | 0.04 | 0.69 | 329 | 0.26 | 0.05 | 1.29 |
| 49340 | Worcester, MA-CT | 375 | 0.21 | 0.05 | 0.84 | 298 | 0.77 | 0.17 | 3.55 | 372 | 0.14 | 0.03 | 0.61 | 81 | 0.32 | 0.06 | 1.64 |
| 49180 | Winston-Salem, NC | 376 | 0.20 | 0.05 | 0.86 | 366 | 0.62 | 0.14 | 2.74 | 373 | 0.13 | 0.03 | 0.64 | 361 | 0.23 | 0.04 | 1.21 |
| 17300 | Clarksville, TN-KY | 377 | 0.20 | 0.04 | 0.93 | 365 | 0.63 | 0.14 | 2.89 | 360 | 0.16 | 0.03 | 0.83 | 304 | 0.27 | 0.05 | 1.46 |
| 14460 | Boston-Cambridge-Newton, MA-NH | 378 | 0.19 | 0.06 | 0.63 | 331 | 0.72 | 0.18 | 2.82 | 374 | 0.13 | 0.03 | 0.48 | 280 | 0.28 | 0.06 | 1.20 |
| 40380 | Rochester, NY | 379 | 0.17 | 0.04 | 0.71 | 368 | 0.62 | 0.14 | 2.72 | 375 | 0.13 | 0.03 | 0.56 | 247 | 0.28 | 0.05 | 1.47 |
| 39300 | Providence-Warwick, RI-MA | 380 | 0.17 | 0.04 | 0.66 | 249 | 0.81 | 0.18 | 3.57 | 380 | 0.10 | 0.02 | 0.43 | 178 | 0.29 | 0.06 | 1.42 |
| 35620 | New York-Newark-Jersey City, NY-NJ-PA | 381 | 0.15 | 0.05 | 0.42 | 378 | 0.51 | 0.16 | 1.57 | 382 | 0.08 | 0.02 | 0.27 | 382 | 0.11 | 0.03 | 0.39 |
| 15380 | Buffalo-Cheektowaga-Niagara Falls, NY | 382 | 0.13 | 0.03 | 0.56 | 377 | 0.53 | 0.12 | 2.32 | 381 | 0.09 | 0.02 | 0.43 | 90 | 0.31 | 0.06 | 1.63 |
|  |  |  |  |  |  |  |  |  |  |  |  |  |  |  |  |  |  |
