# Supplementary material for: Mapping fatal police violence across U.S. metropolitan areas: Overall rates and racial/ethnic inequities, 2013-2017
Source: PLoS One. 2020 Jun 24;15(6):e0229686. doi: 10.1371/journal.pone.0229686 (PMC7313728; doi:10.1371/journal.pone.0229686)
Supplement: S4 Table — (DOCX) [file pone.0229686.s011.docx]

**S4 Table.** Estimated annual IRRs comparing Black and Latinx rates of fatalities involving police to White rates by MSA

| **MSA** | **MSA Name** | **Black-White** | | | | **Latinx-White** | | | |
| --- | --- | --- | --- | --- | --- | --- | --- | --- | --- |
|  |  | **IRR Rank** | **IRR** | **95% CI (Lower Bound)** | **95% CI (Upper Bound)** | **IRR Rank** | **IRR** | **95% CI (Lower Bound)** | **95% CI (Upper Bound)** |
| 21820 | Fairbanks, AK | 240 | 2.86 | 0.61 | 13.48 | 253 | 1.01 | 0.25 | 4.18 |
| 33460 | Minneapolis-St. Paul-Bloomington, MN-WI | 76 | 3.32 | 0.80 | 13.72 | 179 | 1.02 | 0.25 | 4.12 |
| 13900 | Bismarck, ND | 177 | 2.95 | 0.62 | 13.96 | 145 | 1.02 | 0.25 | 4.22 |
| 11260 | Anchorage, AK | 93 | 3.23 | 0.71 | 14.81 | 88 | 1.04 | 0.25 | 4.24 |
| 39660 | Rapid City, SD | 191 | 2.93 | 0.62 | 13.86 | 255 | 1.01 | 0.25 | 4.17 |
| 13740 | Billings, MT | 183 | 2.94 | 0.62 | 13.92 | 303 | 1.00 | 0.24 | 4.10 |
| 39820 | Redding, CA | 216 | 2.90 | 0.61 | 13.70 | 336 | 0.98 | 0.24 | 4.03 |
| 42660 | Seattle-Tacoma-Bellevue, WA | 16 | 3.98 | 1.00 | 15.87 | 348 | 0.97 | 0.25 | 3.79 |
| 32900 | Merced, CA | 306 | 2.75 | 0.59 | 12.85 | 47 | 1.09 | 0.27 | 4.38 |
| 10420 | Akron, OH | 123 | 3.06 | 0.70 | 13.36 | 272 | 1.01 | 0.24 | 4.16 |
| 22380 | Flagstaff, AZ | 184 | 2.94 | 0.62 | 13.92 | 317 | 0.99 | 0.24 | 4.08 |
| 36540 | Omaha-Council Bluffs, NE-IA | 38 | 3.65 | 0.85 | 15.72 | 362 | 0.95 | 0.24 | 3.85 |
| 44060 | Spokane-Spokane Valley, WA | 116 | 3.10 | 0.67 | 14.42 | 349 | 0.97 | 0.24 | 3.96 |
| 23420 | Fresno, CA | 325 | 2.66 | 0.59 | 11.90 | 50 | 1.09 | 0.28 | 4.20 |
| 46520 | Urban Honolulu, HI | 329 | 2.64 | 0.57 | 12.30 | 288 | 1.00 | 0.25 | 4.09 |
| 29340 | Lake Charles, LA | 366 | 2.34 | 0.51 | 10.74 | 183 | 1.02 | 0.25 | 4.21 |
| 44700 | Stockton-Lodi, CA | 48 | 3.54 | 0.80 | 15.60 | 91 | 1.03 | 0.26 | 4.06 |
| 13460 | Bend-Redmond, OR | 150 | 2.97 | 0.63 | 14.11 | 286 | 1.00 | 0.24 | 4.14 |
| 33260 | Midland, TX | 117 | 3.10 | 0.66 | 14.41 | 92 | 1.03 | 0.25 | 4.18 |
| 24500 | Great Falls, MT | 159 | 2.97 | 0.63 | 14.08 | 161 | 1.02 | 0.25 | 4.22 |
| 43580 | Sioux City, IA-NE-SD | 213 | 2.91 | 0.62 | 13.74 | 63 | 1.07 | 0.26 | 4.39 |
| 29740 | Las Cruces, NM | 253 | 2.84 | 0.60 | 13.39 | 26 | 1.15 | 0.29 | 4.58 |
| 46540 | Utica-Rome, NY | 294 | 2.77 | 0.59 | 12.97 | 246 | 1.01 | 0.25 | 4.18 |
| 25260 | Hanford-Corcoran, CA | 290 | 2.77 | 0.59 | 13.01 | 74 | 1.05 | 0.26 | 4.29 |
| 48140 | Wausau, WI | 148 | 2.97 | 0.63 | 14.11 | 164 | 1.02 | 0.25 | 4.22 |
| 20740 | Eau Claire, WI | 188 | 2.94 | 0.62 | 13.90 | 189 | 1.02 | 0.25 | 4.21 |
| 39900 | Reno, NV | 10 | 4.33 | 0.95 | 19.78 | 62 | 1.07 | 0.27 | 4.25 |
| 33660 | Mobile, AL | 49 | 3.53 | 0.81 | 15.42 | 234 | 1.02 | 0.25 | 4.19 |
| 46700 | Vallejo-Fairfield, CA | 115 | 3.10 | 0.70 | 13.79 | 28 | 1.14 | 0.28 | 4.60 |
| 42100 | Santa Cruz-Watsonville, CA | 178 | 2.95 | 0.62 | 13.96 | 330 | 0.99 | 0.24 | 4.01 |
| 29180 | Lafayette, LA | 310 | 2.73 | 0.63 | 11.81 | 59 | 1.07 | 0.26 | 4.41 |
| 11100 | Amarillo, TX | 81 | 3.32 | 0.72 | 15.25 | 44 | 1.10 | 0.27 | 4.44 |
| 22660 | Fort Collins, CO | 214 | 2.90 | 0.61 | 13.71 | 41 | 1.10 | 0.27 | 4.52 |
| 49420 | Yakima, WA | 185 | 2.94 | 0.62 | 13.92 | 21 | 1.16 | 0.29 | 4.68 |
| 24660 | Greensboro-High Point, NC | 369 | 2.25 | 0.50 | 10.01 | 323 | 0.99 | 0.24 | 4.06 |
| 16860 | Chattanooga, TN-GA | 364 | 2.35 | 0.53 | 10.43 | 65 | 1.07 | 0.26 | 4.38 |
| 14500 | Boulder, CO | 190 | 2.93 | 0.62 | 13.87 | 351 | 0.97 | 0.24 | 3.95 |
| 25420 | Harrisburg-Carlisle, PA | 18 | 3.93 | 0.88 | 17.56 | 314 | 0.99 | 0.24 | 4.08 |
| 22140 | Farmington, NM | 166 | 2.96 | 0.62 | 14.04 | 20 | 1.16 | 0.29 | 4.75 |
| 36500 | Olympia-Tumwater, WA | 88 | 3.27 | 0.70 | 15.38 | 300 | 1.00 | 0.24 | 4.11 |
| 41740 | San Diego-Carlsbad, CA | 297 | 2.76 | 0.65 | 11.71 | 29 | 1.14 | 0.31 | 4.24 |
| 17900 | Columbia, SC | 367 | 2.31 | 0.54 | 9.81 | 338 | 0.98 | 0.24 | 4.01 |
| 14860 | Bridgeport-Stamford-Norwalk, CT | 273 | 2.80 | 0.62 | 12.67 | 326 | 0.99 | 0.24 | 4.02 |
| 19740 | Denver-Aurora-Lakewood, CO | 352 | 2.52 | 0.61 | 10.48 | 6 | 1.32 | 0.36 | 4.87 |
| 17020 | Chico, CA | 79 | 3.32 | 0.71 | 15.61 | 236 | 1.01 | 0.25 | 4.14 |
| 28940 | Knoxville, TN | 343 | 2.57 | 0.58 | 11.44 | 340 | 0.97 | 0.24 | 4.00 |
| 12540 | Bakersfield, CA | 360 | 2.42 | 0.56 | 10.58 | 22 | 1.15 | 0.31 | 4.35 |
| 17460 | Cleveland-Elyria, OH | 58 | 3.43 | 0.84 | 13.98 | 370 | 0.95 | 0.23 | 3.86 |
| 49700 | Yuba City, CA | 275 | 2.80 | 0.60 | 13.17 | 341 | 0.97 | 0.24 | 3.96 |
| 10740 | Albuquerque, NM | 238 | 2.86 | 0.64 | 12.89 | 35 | 1.13 | 0.30 | 4.26 |
| 26420 | Houston-The Woodlands-Sugar Land, TX | 210 | 2.91 | 0.83 | 10.16 | 377 | 0.83 | 0.24 | 2.92 |
| 47900 | Washington-Arlington-Alexandria, DC-VA-MD-WV | 31 | 3.80 | 1.00 | 14.39 | 366 | 0.95 | 0.24 | 3.73 |
| 45300 | Tampa-St. Petersburg-Clearwater, FL | 374 | 2.15 | 0.54 | 8.52 | 381 | 0.79 | 0.21 | 3.05 |
| 36420 | Oklahoma City, OK | 26 | 3.83 | 0.98 | 14.95 | 12 | 1.23 | 0.32 | 4.76 |
| 46140 | Tulsa, OK | 114 | 3.10 | 0.75 | 12.90 | 70 | 1.06 | 0.27 | 4.22 |
| 16740 | Charlotte-Concord-Gastonia, NC-SC | 296 | 2.76 | 0.69 | 11.04 | 346 | 0.97 | 0.24 | 3.89 |
| 41620 | Salt Lake City, UT | 66 | 3.38 | 0.73 | 15.54 | 114 | 1.02 | 0.26 | 4.08 |
| 38060 | Phoenix-Mesa-Scottsdale, AZ | 305 | 2.75 | 0.72 | 10.47 | 7 | 1.30 | 0.38 | 4.40 |
| 40140 | Riverside-San Bernardino-Ontario, CA | 351 | 2.53 | 0.66 | 9.70 | 378 | 0.83 | 0.24 | 2.89 |
| 12060 | Atlanta-Sandy Springs-Roswell, GA | 382 | 1.81 | 0.50 | 6.53 | 382 | 0.71 | 0.18 | 2.77 |
| 36740 | Orlando-Kissimmee-Sanford, FL | 313 | 2.71 | 0.67 | 11.08 | 375 | 0.85 | 0.22 | 3.32 |
| 24260 | Grand Island, NE | 147 | 2.98 | 0.63 | 14.12 | 260 | 1.01 | 0.25 | 4.17 |
| 38220 | Pine Bluff, AR | 83 | 3.31 | 0.73 | 15.09 | 108 | 1.02 | 0.25 | 4.24 |
| 36140 | Ocean City, NJ | 194 | 2.93 | 0.62 | 13.86 | 171 | 1.02 | 0.25 | 4.22 |
| 19180 | Danville, IL | 284 | 2.79 | 0.59 | 13.09 | 135 | 1.02 | 0.25 | 4.23 |
| 45540 | The Villages, FL | 236 | 2.87 | 0.61 | 13.52 | 159 | 1.02 | 0.25 | 4.22 |
| 24020 | Glens Falls, NY | 165 | 2.96 | 0.62 | 14.04 | 113 | 1.02 | 0.25 | 4.24 |
| 14100 | Bloomsburg-Berwick, PA | 158 | 2.97 | 0.63 | 14.09 | 110 | 1.02 | 0.25 | 4.24 |
| 28100 | Kankakee, IL | 298 | 2.76 | 0.59 | 12.94 | 211 | 1.02 | 0.25 | 4.20 |
| 23900 | Gettysburg, PA | 149 | 2.97 | 0.63 | 14.11 | 191 | 1.02 | 0.25 | 4.21 |
| 11020 | Altoona, PA | 153 | 2.97 | 0.63 | 14.10 | 96 | 1.03 | 0.25 | 4.24 |
| 19060 | Cumberland, MD-WV | 232 | 2.87 | 0.61 | 13.54 | 99 | 1.03 | 0.25 | 4.24 |
| 16540 | Chambersburg-Waynesboro, PA | 206 | 2.91 | 0.62 | 13.78 | 173 | 1.02 | 0.25 | 4.22 |
| 33140 | Michigan City-La Porte, IN | 265 | 2.82 | 0.60 | 13.25 | 165 | 1.02 | 0.25 | 4.22 |
| 23460 | Gadsden, AL | 309 | 2.73 | 0.58 | 12.77 | 127 | 1.02 | 0.25 | 4.23 |
| 48540 | Wheeling, WV-OH | 207 | 2.91 | 0.62 | 13.77 | 97 | 1.03 | 0.25 | 4.24 |
| 48700 | Williamsport, PA | 68 | 3.37 | 0.71 | 15.90 | 105 | 1.03 | 0.25 | 4.24 |
| 29700 | Laredo, TX | 143 | 2.98 | 0.63 | 14.15 | 72 | 1.06 | 0.26 | 4.25 |
| 33780 | Monroe, MI | 174 | 2.95 | 0.62 | 13.97 | 133 | 1.02 | 0.25 | 4.23 |
| 47460 | Walla Walla, WA | 140 | 2.99 | 0.63 | 14.18 | 245 | 1.01 | 0.25 | 4.18 |
| 33220 | Midland, MI | 142 | 2.98 | 0.63 | 14.15 | 102 | 1.03 | 0.25 | 4.24 |
| 37620 | Parkersburg-Vienna, WV | 156 | 2.97 | 0.63 | 14.09 | 98 | 1.03 | 0.25 | 4.24 |
| 44940 | Sumter, SC | 314 | 2.71 | 0.59 | 12.49 | 122 | 1.02 | 0.25 | 4.23 |
| 44420 | Staunton-Waynesboro, VA | 249 | 2.85 | 0.61 | 13.42 | 132 | 1.02 | 0.25 | 4.23 |
| 40660 | Rome, GA | 100 | 3.18 | 0.68 | 14.89 | 221 | 1.02 | 0.25 | 4.20 |
| 30140 | Lebanon, PA | 168 | 2.95 | 0.62 | 14.00 | 264 | 1.01 | 0.24 | 4.17 |
| 15260 | Brunswick, GA | 136 | 2.99 | 0.64 | 13.88 | 163 | 1.02 | 0.25 | 4.22 |
| 20220 | Dubuque, IA | 180 | 2.94 | 0.62 | 13.95 | 109 | 1.02 | 0.25 | 4.24 |
| 42700 | Sebring, FL | 261 | 2.83 | 0.60 | 13.30 | 279 | 1.01 | 0.24 | 4.15 |
| 47020 | Victoria, TX | 252 | 2.85 | 0.60 | 13.40 | 90 | 1.03 | 0.25 | 4.23 |
| 40580 | Rocky Mount, NC | 354 | 2.52 | 0.55 | 11.49 | 196 | 1.02 | 0.25 | 4.21 |
| 25220 | Hammond, LA | 101 | 3.18 | 0.69 | 14.63 | 151 | 1.02 | 0.25 | 4.22 |
| 16020 | Cape Girardeau, MO-IL | 271 | 2.81 | 0.60 | 13.20 | 111 | 1.02 | 0.25 | 4.24 |
| 22540 | Fond du Lac, WI | 146 | 2.98 | 0.63 | 14.13 | 157 | 1.02 | 0.25 | 4.22 |
| 48260 | Weirton-Steubenville, WV-OH | 23 | 3.89 | 0.83 | 18.29 | 103 | 1.03 | 0.25 | 4.24 |
| 13020 | Bay City, MI | 57 | 3.46 | 0.73 | 16.40 | 185 | 1.02 | 0.25 | 4.21 |
| 34100 | Morristown, TN | 192 | 2.93 | 0.62 | 13.86 | 230 | 1.02 | 0.25 | 4.19 |
| 19140 | Dalton, GA | 221 | 2.89 | 0.61 | 13.66 | 80 | 1.05 | 0.25 | 4.29 |
| 15180 | Brownsville-Harlingen, TX | 152 | 2.97 | 0.63 | 14.10 | 148 | 1.02 | 0.25 | 4.11 |
| 24140 | Goldsboro, NC | 346 | 2.56 | 0.55 | 11.89 | 225 | 1.02 | 0.25 | 4.20 |
| 30620 | Lima, OH | 287 | 2.78 | 0.59 | 13.05 | 115 | 1.02 | 0.25 | 4.23 |
| 15500 | Burlington, NC | 327 | 2.65 | 0.57 | 12.34 | 249 | 1.01 | 0.25 | 4.18 |
| 29020 | Kokomo, IN | 241 | 2.86 | 0.61 | 13.47 | 118 | 1.02 | 0.25 | 4.23 |
| 30300 | Lewiston, ID-WA | 129 | 3.00 | 0.63 | 14.25 | 107 | 1.03 | 0.25 | 4.24 |
| 25620 | Hattiesburg, MS | 125 | 3.05 | 0.67 | 13.94 | 139 | 1.02 | 0.25 | 4.23 |
| 17420 | Cleveland, TN | 235 | 2.87 | 0.61 | 13.53 | 206 | 1.02 | 0.25 | 4.20 |
| 44220 | Springfield, OH | 257 | 2.84 | 0.60 | 13.37 | 117 | 1.02 | 0.25 | 4.23 |
| 46340 | Tyler, TX | 335 | 2.60 | 0.56 | 12.10 | 299 | 1.00 | 0.24 | 4.12 |
| 16220 | Casper, WY | 154 | 2.97 | 0.63 | 14.10 | 228 | 1.02 | 0.25 | 4.19 |
| 20020 | Dothan, AL | 293 | 2.77 | 0.60 | 12.75 | 156 | 1.02 | 0.25 | 4.22 |
| 26300 | Hot Springs, AR | 263 | 2.82 | 0.60 | 13.27 | 178 | 1.02 | 0.25 | 4.22 |
| 27620 | Jefferson City, MO | 269 | 2.81 | 0.60 | 13.21 | 120 | 1.02 | 0.25 | 4.23 |
| 27100 | Jackson, MI | 281 | 2.79 | 0.59 | 13.11 | 144 | 1.02 | 0.25 | 4.22 |
| 22500 | Florence, SC | 324 | 2.66 | 0.59 | 12.07 | 137 | 1.02 | 0.25 | 4.23 |
| 26820 | Idaho Falls, ID | 137 | 2.99 | 0.63 | 14.19 | 278 | 1.01 | 0.24 | 4.15 |
| 42680 | Sebastian-Vero Beach, FL | 99 | 3.19 | 0.68 | 14.91 | 275 | 1.01 | 0.24 | 4.15 |
| 10500 | Albany, GA | 258 | 2.84 | 0.63 | 12.82 | 140 | 1.02 | 0.25 | 4.23 |
| 48060 | Watertown-Fort Drum, NY | 218 | 2.90 | 0.61 | 13.69 | 188 | 1.02 | 0.25 | 4.21 |
| 30340 | Lewiston-Auburn, ME | 151 | 2.97 | 0.63 | 14.11 | 100 | 1.03 | 0.25 | 4.24 |
| 46220 | Tuscaloosa, AL | 304 | 2.75 | 0.60 | 12.46 | 187 | 1.02 | 0.25 | 4.21 |
| 38340 | Pittsfield, MA | 186 | 2.94 | 0.62 | 13.91 | 155 | 1.02 | 0.25 | 4.22 |
| 13220 | Beckley, WV | 97 | 3.20 | 0.68 | 15.00 | 112 | 1.02 | 0.25 | 4.24 |
| 25940 | Hilton Head Island-Bluffton-Beaufort, SC | 283 | 2.79 | 0.61 | 12.84 | 53 | 1.08 | 0.26 | 4.46 |
| 23580 | Gainesville, GA | 280 | 2.79 | 0.59 | 13.11 | 73 | 1.05 | 0.26 | 4.33 |
| 41660 | San Angelo, TX | 77 | 3.32 | 0.71 | 15.63 | 104 | 1.03 | 0.25 | 4.19 |
| 15680 | California-Lexington Park, MD | 292 | 2.77 | 0.59 | 13.00 | 141 | 1.02 | 0.25 | 4.23 |
| 27180 | Jackson, TN | 112 | 3.11 | 0.69 | 14.10 | 208 | 1.02 | 0.25 | 4.20 |
| 21300 | Elmira, NY | 230 | 2.88 | 0.61 | 13.58 | 116 | 1.02 | 0.25 | 4.23 |
| 10540 | Albany, OR | 131 | 3.00 | 0.63 | 14.23 | 209 | 1.02 | 0.25 | 4.20 |
| 27780 | Johnstown, PA | 239 | 2.86 | 0.61 | 13.48 | 126 | 1.02 | 0.25 | 4.23 |
| 25500 | Harrisonburg, VA | 212 | 2.91 | 0.62 | 13.75 | 254 | 1.01 | 0.25 | 4.18 |
| 33740 | Monroe, LA | 311 | 2.72 | 0.61 | 12.21 | 184 | 1.02 | 0.25 | 4.21 |
| 25980 | Hinesville, GA | 201 | 2.92 | 0.63 | 13.55 | 217 | 1.02 | 0.25 | 4.20 |
| 34620 | Muncie, IN | 279 | 2.80 | 0.60 | 13.13 | 124 | 1.02 | 0.25 | 4.23 |
| 43300 | Sherman-Denison, TX | 223 | 2.89 | 0.61 | 13.66 | 247 | 1.01 | 0.25 | 4.18 |
| 34740 | Muskegon, MI | 315 | 2.71 | 0.58 | 12.69 | 182 | 1.02 | 0.25 | 4.21 |
| 27500 | Janesville-Beloit, WI | 71 | 3.35 | 0.71 | 15.80 | 239 | 1.01 | 0.25 | 4.19 |
| 16180 | Carson City, NV | 145 | 2.98 | 0.63 | 14.13 | 277 | 1.01 | 0.24 | 4.15 |
| 31420 | Macon, GA | 345 | 2.57 | 0.57 | 11.61 | 149 | 1.02 | 0.25 | 4.22 |
| 40980 | Saginaw, MI | 344 | 2.57 | 0.55 | 11.91 | 242 | 1.01 | 0.25 | 4.18 |
| 16940 | Cheyenne, WY | 196 | 2.93 | 0.62 | 13.85 | 283 | 1.01 | 0.24 | 4.14 |
| 46660 | Valdosta, GA | 337 | 2.59 | 0.57 | 11.88 | 45 | 1.09 | 0.27 | 4.52 |
| 34940 | Naples-Immokalee-Marco Island, FL | 319 | 2.70 | 0.58 | 12.60 | 94 | 1.03 | 0.25 | 4.21 |
| 31860 | Mankato-North Mankato, MN | 208 | 2.91 | 0.62 | 13.76 | 153 | 1.02 | 0.25 | 4.22 |
| 47940 | Waterloo-Cedar Falls, IA | 270 | 2.81 | 0.60 | 13.20 | 152 | 1.02 | 0.25 | 4.22 |
| 27860 | Jonesboro, AR | 317 | 2.71 | 0.58 | 12.67 | 175 | 1.02 | 0.25 | 4.22 |
| 45500 | Texarkana, TX-AR | 107 | 3.15 | 0.69 | 14.46 | 216 | 1.02 | 0.25 | 4.20 |
| 39540 | Racine, WI | 318 | 2.71 | 0.58 | 12.67 | 281 | 1.01 | 0.24 | 4.15 |
| 38540 | Pocatello, ID | 133 | 2.99 | 0.63 | 14.21 | 197 | 1.02 | 0.25 | 4.21 |
| 19460 | Decatur, AL | 312 | 2.72 | 0.58 | 12.71 | 210 | 1.02 | 0.25 | 4.20 |
| 41100 | St. George, UT | 141 | 2.98 | 0.63 | 14.17 | 256 | 1.01 | 0.25 | 4.17 |
| 34580 | Mount Vernon-Anacortes, WA | 138 | 2.99 | 0.63 | 14.19 | 274 | 1.01 | 0.24 | 4.16 |
| 19500 | Decatur, IL | 288 | 2.78 | 0.59 | 13.04 | 101 | 1.03 | 0.25 | 4.24 |
| 39460 | Punta Gorda, FL | 277 | 2.80 | 0.60 | 13.14 | 248 | 1.01 | 0.25 | 4.18 |
| 12700 | Barnstable Town, MA | 200 | 2.92 | 0.62 | 13.83 | 136 | 1.02 | 0.25 | 4.23 |
| 31740 | Manhattan, KS | 204 | 2.92 | 0.62 | 13.80 | 177 | 1.02 | 0.25 | 4.22 |
| 10780 | Alexandria, LA | 211 | 2.91 | 0.64 | 13.27 | 170 | 1.02 | 0.25 | 4.22 |
| 36980 | Owensboro, KY | 75 | 3.33 | 0.71 | 15.67 | 147 | 1.02 | 0.25 | 4.22 |
| 49020 | Winchester, VA-WV | 84 | 3.31 | 0.70 | 15.55 | 257 | 1.01 | 0.25 | 4.17 |
| 26140 | Homosassa Springs, FL | 225 | 2.89 | 0.61 | 13.63 | 232 | 1.02 | 0.25 | 4.19 |
| 24780 | Greenville, NC | 334 | 2.60 | 0.57 | 11.93 | 213 | 1.02 | 0.25 | 4.20 |
| 24220 | Grand Forks, ND-MN | 181 | 2.94 | 0.62 | 13.93 | 150 | 1.02 | 0.25 | 4.22 |
| 35100 | New Bern, NC | 197 | 2.93 | 0.63 | 13.55 | 205 | 1.02 | 0.25 | 4.20 |
| 16060 | Carbondale-Marion, IL | 291 | 2.77 | 0.59 | 13.01 | 143 | 1.02 | 0.25 | 4.22 |
| 20940 | El Centro, CA | 67 | 3.37 | 0.71 | 15.90 | 86 | 1.04 | 0.26 | 4.21 |
| 17660 | Coeur d'Alene, ID | 134 | 2.99 | 0.63 | 14.21 | 193 | 1.02 | 0.25 | 4.21 |
| 41060 | St. Cloud, MN | 86 | 3.30 | 0.70 | 15.49 | 162 | 1.02 | 0.25 | 4.22 |
| 45460 | Terre Haute, IN | 243 | 2.85 | 0.61 | 13.45 | 129 | 1.02 | 0.25 | 4.23 |
| 20700 | East Stroudsburg, PA | 328 | 2.64 | 0.57 | 12.32 | 294 | 1.00 | 0.24 | 4.13 |
| 36220 | Odessa, TX | 285 | 2.79 | 0.59 | 13.07 | 289 | 1.00 | 0.25 | 4.07 |
| 12980 | Battle Creek, MI | 295 | 2.77 | 0.59 | 12.97 | 172 | 1.02 | 0.25 | 4.22 |
| 18020 | Columbus, IN | 155 | 2.97 | 0.63 | 14.09 | 168 | 1.02 | 0.25 | 4.22 |
| 29100 | La Crosse-Onalaska, WI-MN | 157 | 2.97 | 0.63 | 14.09 | 106 | 1.03 | 0.25 | 4.24 |
| 33540 | Missoula, MT | 132 | 2.99 | 0.63 | 14.23 | 125 | 1.02 | 0.25 | 4.23 |
| 10180 | Abilene, TX | 308 | 2.73 | 0.58 | 12.78 | 335 | 0.98 | 0.24 | 4.03 |
| 28740 | Kingston, NY | 247 | 2.85 | 0.61 | 13.43 | 259 | 1.01 | 0.25 | 4.17 |
| 24420 | Grants Pass, OR | 135 | 2.99 | 0.63 | 14.21 | 233 | 1.02 | 0.25 | 4.19 |
| 44100 | Springfield, IL | 333 | 2.63 | 0.56 | 12.24 | 134 | 1.02 | 0.25 | 4.23 |
| 26580 | Huntington-Ashland, WV-KY-OH | 245 | 2.85 | 0.61 | 13.44 | 131 | 1.02 | 0.25 | 4.23 |
| 19300 | Daphne-Fairhope-Foley, AL | 340 | 2.58 | 0.56 | 11.96 | 240 | 1.01 | 0.25 | 4.19 |
| 30860 | Logan, UT-ID | 144 | 2.98 | 0.63 | 14.14 | 273 | 1.01 | 0.24 | 4.16 |
| 22520 | Florence-Muscle Shoals, AL | 358 | 2.46 | 0.53 | 11.36 | 192 | 1.02 | 0.25 | 4.21 |
| 43100 | Sheboygan, WI | 162 | 2.97 | 0.63 | 14.07 | 190 | 1.02 | 0.25 | 4.21 |
| 18700 | Corvallis, OR | 139 | 2.99 | 0.63 | 14.18 | 166 | 1.02 | 0.25 | 4.22 |
| 27740 | Johnson City, TN | 251 | 2.85 | 0.60 | 13.40 | 204 | 1.02 | 0.25 | 4.21 |
| 48300 | Wenatchee, WA | 51 | 3.50 | 0.74 | 16.60 | 353 | 0.96 | 0.24 | 3.95 |
| 21420 | Enid, OK | 176 | 2.95 | 0.62 | 13.97 | 226 | 1.02 | 0.25 | 4.20 |
| 34060 | Morgantown, WV | 233 | 2.87 | 0.61 | 13.53 | 130 | 1.02 | 0.25 | 4.23 |
| 41140 | St. Joseph, MO-KS | 282 | 2.79 | 0.59 | 13.09 | 237 | 1.01 | 0.25 | 4.19 |
| 33860 | Montgomery, AL | 363 | 2.37 | 0.53 | 10.65 | 195 | 1.02 | 0.25 | 4.21 |
| 21780 | Evansville, IN-KY | 108 | 3.14 | 0.67 | 14.63 | 158 | 1.02 | 0.25 | 4.22 |
| 12020 | Athens-Clarke County, GA | 95 | 3.22 | 0.70 | 14.83 | 251 | 1.01 | 0.25 | 4.18 |
| 14540 | Bowling Green, KY | 302 | 2.75 | 0.59 | 12.88 | 203 | 1.02 | 0.25 | 4.21 |
| 44300 | State College, PA | 205 | 2.91 | 0.62 | 13.78 | 121 | 1.02 | 0.25 | 4.23 |
| 24300 | Grand Junction, CO | 164 | 2.96 | 0.63 | 14.05 | 33 | 1.14 | 0.28 | 4.66 |
| 36780 | Oshkosh-Neenah, WI | 63 | 3.40 | 0.72 | 16.07 | 198 | 1.02 | 0.25 | 4.21 |
| 27340 | Jacksonville, NC | 321 | 2.69 | 0.58 | 12.57 | 263 | 1.01 | 0.24 | 4.17 |
| 35660 | Niles-Benton Harbor, MI | 32 | 3.79 | 0.82 | 17.45 | 220 | 1.02 | 0.25 | 4.20 |
| 47580 | Warner Robins, GA | 350 | 2.53 | 0.55 | 11.56 | 238 | 1.01 | 0.25 | 4.19 |
| 31900 | Mansfield, OH | 41 | 3.61 | 0.77 | 16.78 | 128 | 1.02 | 0.25 | 4.23 |
| 16300 | Cedar Rapids, IA | 87 | 3.28 | 0.70 | 15.38 | 176 | 1.02 | 0.25 | 4.22 |
| 28700 | Kingsport-Bristol-Bristol, TN-VA | 254 | 2.84 | 0.60 | 13.38 | 186 | 1.02 | 0.25 | 4.21 |
| 21060 | Elizabethtown-Fort Knox, KY | 109 | 3.13 | 0.67 | 14.61 | 212 | 1.02 | 0.25 | 4.20 |
| 17300 | Clarksville, TN-KY | 349 | 2.53 | 0.55 | 11.74 | 252 | 1.01 | 0.25 | 4.18 |
| 49740 | Yuma, AZ | 217 | 2.90 | 0.61 | 13.69 | 324 | 0.99 | 0.24 | 4.00 |
| 48900 | Wilmington, NC | 53 | 3.49 | 0.77 | 15.90 | 266 | 1.01 | 0.24 | 4.16 |
| 32580 | McAllen-Edinburg-Mission, TX | 195 | 2.93 | 0.62 | 13.85 | 258 | 1.01 | 0.25 | 4.02 |
| 48660 | Wichita Falls, TX | 322 | 2.69 | 0.58 | 12.56 | 5 | 1.32 | 0.32 | 5.40 |
| 11180 | Ames, IA | 163 | 2.96 | 0.63 | 14.05 | 119 | 1.02 | 0.25 | 4.23 |
| 29940 | Lawrence, KS | 199 | 2.93 | 0.62 | 13.84 | 169 | 1.02 | 0.25 | 4.22 |
| 39740 | Reading, PA | 104 | 3.17 | 0.68 | 14.80 | 93 | 1.03 | 0.25 | 4.21 |
| 31460 | Madera, CA | 231 | 2.87 | 0.61 | 13.54 | 84 | 1.04 | 0.26 | 4.24 |
| 21500 | Erie, PA | 42 | 3.58 | 0.77 | 16.67 | 219 | 1.02 | 0.25 | 4.20 |
| 13780 | Binghamton, NY | 244 | 2.85 | 0.61 | 13.44 | 181 | 1.02 | 0.25 | 4.21 |
| 27900 | Joplin, MO | 182 | 2.94 | 0.62 | 13.93 | 244 | 1.01 | 0.25 | 4.18 |
| 47220 | Vineland-Bridgeton, NJ | 44 | 3.56 | 0.78 | 16.27 | 354 | 0.96 | 0.24 | 3.95 |
| 40220 | Roanoke, VA | 91 | 3.24 | 0.71 | 14.92 | 215 | 1.02 | 0.25 | 4.20 |
| 25180 | Hagerstown-Martinsburg, MD-WV | 54 | 3.49 | 0.75 | 16.17 | 223 | 1.02 | 0.25 | 4.20 |
| 40340 | Rochester, MN | 234 | 2.87 | 0.61 | 13.53 | 194 | 1.02 | 0.25 | 4.21 |
| 11500 | Anniston-Oxford-Jacksonville, AL | 361 | 2.41 | 0.53 | 10.91 | 243 | 1.01 | 0.25 | 4.18 |
| 22020 | Fargo, ND-MN | 248 | 2.85 | 0.61 | 13.42 | 174 | 1.02 | 0.25 | 4.22 |
| 15940 | Canton-Massillon, OH | 326 | 2.65 | 0.57 | 12.36 | 167 | 1.02 | 0.25 | 4.22 |
| 12220 | Auburn-Opelika, AL | 161 | 2.97 | 0.65 | 13.53 | 199 | 1.02 | 0.25 | 4.21 |
| 49180 | Winston-Salem, NC | 259 | 2.83 | 0.62 | 12.83 | 315 | 0.99 | 0.24 | 4.08 |
| 42540 | Scranton--Wilkes-Barre--Hazleton, PA | 289 | 2.78 | 0.59 | 13.03 | 64 | 1.07 | 0.26 | 4.38 |
| 43420 | Sierra Vista-Douglas, AZ | 246 | 2.85 | 0.61 | 13.43 | 8 | 1.25 | 0.31 | 5.11 |
| 31340 | Lynchburg, VA | 347 | 2.56 | 0.56 | 11.67 | 43 | 1.10 | 0.27 | 4.53 |
| 12620 | Bangor, ME | 171 | 2.95 | 0.62 | 13.99 | 138 | 1.02 | 0.25 | 4.23 |
| 27060 | Ithaca, NY | 187 | 2.94 | 0.62 | 13.91 | 142 | 1.02 | 0.25 | 4.23 |
| 43620 | Sioux Falls, SD | 250 | 2.85 | 0.60 | 13.40 | 207 | 1.02 | 0.25 | 4.20 |
| 30980 | Longview, TX | 376 | 2.08 | 0.46 | 9.44 | 42 | 1.10 | 0.27 | 4.48 |
| 21140 | Elkhart-Goshen, IN | 47 | 3.54 | 0.76 | 16.43 | 38 | 1.12 | 0.27 | 4.59 |
| 29200 | Lafayette-West Lafayette, IN | 229 | 2.88 | 0.61 | 13.61 | 235 | 1.02 | 0.25 | 4.19 |
| 37460 | Panama City, FL | 172 | 2.95 | 0.64 | 13.66 | 250 | 1.01 | 0.25 | 4.18 |
| 14010 | Bloomington, IL | 299 | 2.76 | 0.59 | 12.93 | 202 | 1.02 | 0.25 | 4.21 |
| 43780 | South Bend-Mishawaka, IN-MI | 226 | 2.89 | 0.63 | 13.33 | 282 | 1.01 | 0.24 | 4.15 |
| 16620 | Charleston, WV | 331 | 2.63 | 0.57 | 12.25 | 123 | 1.02 | 0.25 | 4.23 |
| 36100 | Ocala, FL | 362 | 2.39 | 0.52 | 11.00 | 34 | 1.13 | 0.28 | 4.65 |
| 37900 | Peoria, IL | 173 | 2.95 | 0.64 | 13.65 | 218 | 1.02 | 0.25 | 4.20 |
| 49660 | Youngstown-Warren-Boardman, OH-PA | 179 | 2.95 | 0.65 | 13.38 | 262 | 1.01 | 0.24 | 4.17 |
| 31180 | Lubbock, TX | 59 | 3.42 | 0.74 | 15.80 | 17 | 1.19 | 0.29 | 4.81 |
| 22420 | Flint, MI | 11 | 4.21 | 0.94 | 18.81 | 231 | 1.02 | 0.25 | 4.19 |
| 39380 | Pueblo, CO | 237 | 2.87 | 0.61 | 13.51 | 1 | 1.50 | 0.37 | 6.00 |
| 28140 | Kansas City, MO-KS | 96 | 3.21 | 0.82 | 12.67 | 87 | 1.04 | 0.26 | 4.12 |
| 40420 | Rockford, IL | 89 | 3.25 | 0.71 | 14.95 | 328 | 0.99 | 0.24 | 4.05 |
| 11540 | Appleton, WI | 15 | 4.01 | 0.85 | 18.98 | 224 | 1.02 | 0.25 | 4.20 |
| 41540 | Salisbury, MD-DE | 19 | 3.92 | 0.87 | 17.59 | 292 | 1.00 | 0.24 | 4.13 |
| 15540 | Burlington-South Burlington, VT | 220 | 2.90 | 0.61 | 13.67 | 160 | 1.02 | 0.25 | 4.22 |
| 16820 | Charlottesville, VA | 82 | 3.31 | 0.72 | 15.26 | 229 | 1.02 | 0.25 | 4.19 |
| 17780 | College Station-Bryan, TX | 127 | 3.02 | 0.65 | 14.01 | 78 | 1.05 | 0.26 | 4.30 |
| 27140 | Jackson, MS | 372 | 2.18 | 0.51 | 9.35 | 261 | 1.01 | 0.25 | 4.17 |
| 14020 | Bloomington, IN | 64 | 3.39 | 0.72 | 16.00 | 154 | 1.02 | 0.25 | 4.22 |
| 43340 | Shreveport-Bossier City, LA | 342 | 2.57 | 0.58 | 11.29 | 265 | 1.01 | 0.24 | 4.16 |
| 26980 | Iowa City, IA | 276 | 2.80 | 0.60 | 13.14 | 46 | 1.09 | 0.26 | 4.51 |
| 31020 | Longview, WA | 56 | 3.47 | 0.73 | 16.46 | 285 | 1.01 | 0.24 | 4.14 |
| 42020 | San Luis Obispo-Paso Robles-Arroyo Grande, CA | 203 | 2.92 | 0.62 | 13.81 | 81 | 1.05 | 0.25 | 4.29 |
| 20100 | Dover, DE | 274 | 2.80 | 0.62 | 12.73 | 270 | 1.01 | 0.24 | 4.16 |
| 38940 | Port St. Lucie, FL | 12 | 4.14 | 0.93 | 18.40 | 364 | 0.95 | 0.23 | 3.89 |
| 19340 | Davenport-Moline-Rock Island, IA-IL | 189 | 2.93 | 0.63 | 13.55 | 307 | 0.99 | 0.24 | 4.09 |
| 32780 | Medford, OR | 160 | 2.97 | 0.63 | 14.07 | 322 | 0.99 | 0.24 | 4.06 |
| 24540 | Greeley, CO | 65 | 3.38 | 0.72 | 15.96 | 2 | 1.41 | 0.35 | 5.69 |
| 31700 | Manchester-Nashua, NH | 228 | 2.89 | 0.61 | 13.62 | 271 | 1.01 | 0.24 | 4.16 |
| 28020 | Kalamazoo-Portage, MI | 336 | 2.59 | 0.56 | 12.05 | 268 | 1.01 | 0.24 | 4.16 |
| 11700 | Asheville, NC | 8 | 4.48 | 0.98 | 20.57 | 316 | 0.99 | 0.24 | 4.08 |
| 43900 | Spartanburg, SC | 262 | 2.82 | 0.63 | 12.64 | 295 | 1.00 | 0.24 | 4.12 |
| 29540 | Lancaster, PA | 113 | 3.11 | 0.67 | 14.50 | 77 | 1.05 | 0.26 | 4.31 |
| 34820 | Myrtle Beach-Conway-North Myrtle Beach, SC-NC | 359 | 2.43 | 0.54 | 11.00 | 305 | 1.00 | 0.24 | 4.10 |
| 20260 | Duluth, MN-WI | 209 | 2.91 | 0.62 | 13.76 | 146 | 1.02 | 0.25 | 4.22 |
| 30460 | Lexington-Fayette, KY | 357 | 2.46 | 0.53 | 11.36 | 52 | 1.08 | 0.26 | 4.46 |
| 13980 | Blacksburg-Christiansburg-Radford, VA | 301 | 2.75 | 0.59 | 12.89 | 200 | 1.02 | 0.25 | 4.21 |
| 18580 | Corpus Christi, TX | 121 | 3.07 | 0.66 | 14.25 | 60 | 1.07 | 0.27 | 4.27 |
| 39140 | Prescott, AZ | 167 | 2.96 | 0.62 | 14.02 | 350 | 0.97 | 0.24 | 3.96 |
| 49620 | York-Hanover, PA | 34 | 3.77 | 0.82 | 17.27 | 325 | 0.99 | 0.24 | 4.06 |
| 42140 | Santa Fe, NM | 170 | 2.95 | 0.62 | 14.00 | 9 | 1.25 | 0.31 | 5.04 |
| 45220 | Tallahassee, FL | 106 | 3.15 | 0.72 | 13.88 | 297 | 1.00 | 0.24 | 4.12 |
| 34900 | Napa, CA | 60 | 3.41 | 0.72 | 16.14 | 89 | 1.04 | 0.25 | 4.24 |
| 16580 | Champaign-Urbana, IL | 122 | 3.06 | 0.66 | 14.24 | 214 | 1.02 | 0.25 | 4.20 |
| 44140 | Springfield, MA | 70 | 3.37 | 0.73 | 15.52 | 365 | 0.95 | 0.23 | 3.89 |
| 18880 | Crestview-Fort Walton Beach-Destin, FL | 169 | 2.95 | 0.64 | 13.67 | 296 | 1.00 | 0.24 | 4.12 |
| 17980 | Columbus, GA-AL | 368 | 2.27 | 0.51 | 10.10 | 57 | 1.08 | 0.26 | 4.43 |
| 26380 | Houma-Thibodaux, LA | 330 | 2.63 | 0.58 | 12.04 | 48 | 1.09 | 0.26 | 4.49 |
| 17860 | Columbia, MO | 55 | 3.47 | 0.75 | 16.09 | 201 | 1.02 | 0.25 | 4.21 |
| 38860 | Portland-South Portland, ME | 92 | 3.24 | 0.69 | 15.19 | 227 | 1.02 | 0.25 | 4.19 |
| 45780 | Toledo, OH | 316 | 2.71 | 0.60 | 12.23 | 310 | 0.99 | 0.24 | 4.09 |
| 47380 | Waco, TX | 323 | 2.66 | 0.60 | 11.82 | 68 | 1.06 | 0.26 | 4.28 |
| 24580 | Green Bay, WI | 227 | 2.89 | 0.61 | 13.62 | 291 | 1.00 | 0.24 | 4.13 |
| 45820 | Topeka, KS | 21 | 3.90 | 0.85 | 17.95 | 327 | 0.99 | 0.24 | 4.05 |
| 41700 | San Antonio-New Braunfels, TX | 198 | 2.93 | 0.72 | 11.93 | 368 | 0.95 | 0.26 | 3.45 |
| 15980 | Cape Coral-Fort Myers, FL | 266 | 2.81 | 0.62 | 12.70 | 374 | 0.90 | 0.22 | 3.63 |
| 13380 | Bellingham, WA | 14 | 4.03 | 0.85 | 19.09 | 54 | 1.08 | 0.26 | 4.45 |
| 15380 | Buffalo-Cheektowaga-Niagara Falls, NY | 339 | 2.58 | 0.57 | 11.76 | 55 | 1.08 | 0.26 | 4.45 |
| 30700 | Lincoln, NE | 36 | 3.69 | 0.79 | 17.20 | 290 | 1.00 | 0.24 | 4.13 |
| 29460 | Lakeland-Winter Haven, FL | 378 | 1.95 | 0.43 | 8.78 | 355 | 0.96 | 0.24 | 3.91 |
| 21340 | El Paso, TX | 37 | 3.67 | 0.80 | 16.77 | 342 | 0.97 | 0.25 | 3.83 |
| 13140 | Beaumont-Port Arthur, TX | 377 | 2.07 | 0.47 | 9.16 | 23 | 1.15 | 0.28 | 4.69 |
| 42340 | Savannah, GA | 303 | 2.75 | 0.63 | 11.99 | 306 | 0.99 | 0.24 | 4.09 |
| 28420 | Kennewick-Richland, WA | 80 | 3.32 | 0.71 | 15.60 | 359 | 0.96 | 0.24 | 3.89 |
| 45060 | Syracuse, NY | 40 | 3.63 | 0.79 | 16.56 | 269 | 1.01 | 0.24 | 4.16 |
| 23540 | Gainesville, FL | 105 | 3.16 | 0.70 | 14.30 | 301 | 1.00 | 0.24 | 4.10 |
| 25060 | Gulfport-Biloxi-Pascagoula, MS | 286 | 2.78 | 0.63 | 12.29 | 304 | 1.00 | 0.24 | 4.10 |
| 35980 | Norwich-New London, CT | 111 | 3.12 | 0.67 | 14.52 | 311 | 0.99 | 0.24 | 4.09 |
| 12260 | Augusta-Richmond County, GA-SC | 379 | 1.92 | 0.44 | 8.38 | 309 | 0.99 | 0.24 | 4.09 |
| 35300 | New Haven-Milford, CT | 341 | 2.57 | 0.56 | 11.70 | 180 | 1.02 | 0.25 | 4.17 |
| 12420 | Austin-Round Rock, TX | 353 | 2.52 | 0.59 | 10.74 | 380 | 0.82 | 0.21 | 3.15 |
| 40380 | Rochester, NY | 272 | 2.81 | 0.62 | 12.68 | 69 | 1.06 | 0.26 | 4.35 |
| 30020 | Lawton, OK | 90 | 3.25 | 0.71 | 14.95 | 298 | 1.00 | 0.24 | 4.12 |
| 29420 | Lake Havasu City-Kingman, AZ | 224 | 2.89 | 0.61 | 13.63 | 293 | 1.00 | 0.25 | 4.08 |
| 21660 | Eugene, OR | 202 | 2.92 | 0.62 | 13.83 | 312 | 0.99 | 0.24 | 4.08 |
| 25860 | Hickory-Lenoir-Morganton, NC | 307 | 2.73 | 0.60 | 12.52 | 334 | 0.98 | 0.24 | 4.03 |
| 29820 | Las Vegas-Henderson-Paradise, NV | 356 | 2.51 | 0.63 | 9.97 | 379 | 0.82 | 0.22 | 3.09 |
| 14740 | Bremerton-Silverdale, WA | 72 | 3.34 | 0.71 | 15.76 | 280 | 1.01 | 0.24 | 4.15 |
| 23060 | Fort Wayne, IN | 13 | 4.09 | 0.91 | 18.31 | 66 | 1.06 | 0.26 | 4.37 |
| 44180 | Springfield, MO | 119 | 3.09 | 0.66 | 14.38 | 56 | 1.08 | 0.26 | 4.43 |
| 35840 | North Port-Sarasota-Bradenton, FL | 118 | 3.09 | 0.69 | 13.85 | 82 | 1.04 | 0.26 | 4.24 |
| 12100 | Atlantic City-Hammonton, NJ | 27 | 3.83 | 0.85 | 17.36 | 347 | 0.97 | 0.24 | 3.97 |
| 20500 | Durham-Chapel Hill, NC | 43 | 3.58 | 0.82 | 15.68 | 36 | 1.12 | 0.27 | 4.60 |
| 11460 | Ann Arbor, MI | 35 | 3.70 | 0.81 | 16.96 | 241 | 1.01 | 0.25 | 4.19 |
| 41420 | Salem, OR | 222 | 2.89 | 0.61 | 13.66 | 344 | 0.97 | 0.24 | 3.95 |
| 28660 | Killeen-Temple, TX | 355 | 2.51 | 0.56 | 11.25 | 319 | 0.99 | 0.24 | 4.03 |
| 29620 | Lansing-East Lansing, MI | 278 | 2.80 | 0.61 | 12.84 | 61 | 1.07 | 0.26 | 4.41 |
| 16700 | Charleston-North Charleston, SC | 102 | 3.18 | 0.75 | 13.50 | 76 | 1.05 | 0.26 | 4.32 |
| 49340 | Worcester, MA-CT | 128 | 3.00 | 0.65 | 13.90 | 39 | 1.11 | 0.27 | 4.53 |
| 19780 | Des Moines-West Des Moines, IA | 215 | 2.90 | 0.63 | 13.38 | 331 | 0.98 | 0.24 | 4.04 |
| 22220 | Fayetteville-Springdale-Rogers, AR-MO | 94 | 3.23 | 0.69 | 15.13 | 363 | 0.95 | 0.23 | 3.89 |
| 26620 | Huntsville, AL | 219 | 2.90 | 0.67 | 12.58 | 320 | 0.99 | 0.24 | 4.07 |
| 39340 | Provo-Orem, UT | 62 | 3.40 | 0.72 | 16.06 | 287 | 1.00 | 0.25 | 4.09 |
| 33100 | Miami-Fort Lauderdale-West Palm Beach, FL | 50 | 3.52 | 0.96 | 12.88 | 376 | 0.85 | 0.23 | 3.10 |
| 22180 | Fayetteville, NC | 20 | 3.92 | 0.90 | 17.02 | 339 | 0.98 | 0.24 | 4.00 |
| 12940 | Baton Rouge, LA | 69 | 3.37 | 0.82 | 13.89 | 329 | 0.99 | 0.24 | 4.05 |
| 37340 | Palm Bay-Melbourne-Titusville, FL | 22 | 3.90 | 0.89 | 17.14 | 367 | 0.95 | 0.23 | 3.88 |
| 47300 | Visalia-Porterville, CA | 268 | 2.81 | 0.60 | 13.22 | 30 | 1.14 | 0.29 | 4.51 |
| 36260 | Ogden-Clearfield, UT | 267 | 2.81 | 0.60 | 13.22 | 37 | 1.12 | 0.28 | 4.55 |
| 13820 | Birmingham-Hoover, AL | 370 | 2.24 | 0.54 | 9.29 | 345 | 0.97 | 0.24 | 3.97 |
| 10900 | Allentown-Bethlehem-Easton, PA-NJ | 126 | 3.03 | 0.67 | 13.77 | 361 | 0.95 | 0.24 | 3.86 |
| 22900 | Fort Smith, AR-OK | 300 | 2.75 | 0.59 | 12.89 | 313 | 0.99 | 0.24 | 4.08 |
| 42200 | Santa Maria-Santa Barbara, CA | 264 | 2.82 | 0.60 | 13.25 | 18 | 1.17 | 0.29 | 4.70 |
| 37860 | Pensacola-Ferry Pass-Brent, FL | 375 | 2.14 | 0.48 | 9.58 | 318 | 0.99 | 0.24 | 4.07 |
| 14260 | Boise City, ID | 260 | 2.83 | 0.60 | 13.29 | 19 | 1.17 | 0.29 | 4.75 |
| 24340 | Grand Rapids-Wyoming, MI | 46 | 3.54 | 0.79 | 15.82 | 267 | 1.01 | 0.25 | 4.12 |
| 45940 | Trenton, NJ | 7 | 4.69 | 1.06 | 20.71 | 358 | 0.96 | 0.23 | 3.93 |
| 25540 | Hartford-West Hartford-East Hartford, CT | 332 | 2.63 | 0.59 | 11.79 | 67 | 1.06 | 0.26 | 4.32 |
| 31540 | Madison, WI | 85 | 3.30 | 0.72 | 15.17 | 31 | 1.14 | 0.28 | 4.67 |
| 42220 | Santa Rosa, CA | 255 | 2.84 | 0.60 | 13.37 | 14 | 1.22 | 0.30 | 4.90 |
| 30780 | Little Rock-North Little Rock-Conway, AR | 338 | 2.59 | 0.62 | 10.85 | 352 | 0.96 | 0.24 | 3.95 |
| 24860 | Greenville-Anderson-Mauldin, SC | 348 | 2.54 | 0.60 | 10.72 | 284 | 1.01 | 0.25 | 4.10 |
| 37980 | Philadelphia-Camden-Wilmington, PA-NJ-DE-MD | 33 | 3.78 | 0.99 | 14.39 | 333 | 0.98 | 0.25 | 3.91 |
| 39300 | Providence-Warwick, RI-MA | 45 | 3.54 | 0.78 | 16.10 | 27 | 1.14 | 0.28 | 4.65 |
| 19660 | Deltona-Daytona Beach-Ormond Beach, FL | 373 | 2.17 | 0.49 | 9.54 | 371 | 0.95 | 0.23 | 3.82 |
| 31140 | Louisville/Jefferson County, KY-IN | 130 | 3.00 | 0.70 | 12.80 | 343 | 0.97 | 0.24 | 3.99 |
| 17820 | Colorado Springs, CO | 120 | 3.07 | 0.67 | 13.97 | 337 | 0.98 | 0.24 | 3.97 |
| 19380 | Dayton, OH | 9 | 4.44 | 1.06 | 18.57 | 302 | 1.00 | 0.24 | 4.10 |
| 40060 | Richmond, VA | 74 | 3.33 | 0.79 | 13.99 | 83 | 1.04 | 0.25 | 4.28 |
| 41500 | Salinas, CA | 320 | 2.70 | 0.58 | 12.59 | 24 | 1.15 | 0.29 | 4.57 |
| 35380 | New Orleans-Metairie, LA | 39 | 3.64 | 0.90 | 14.71 | 332 | 0.98 | 0.24 | 3.99 |
| 39580 | Raleigh, NC | 371 | 2.22 | 0.51 | 9.74 | 369 | 0.95 | 0.23 | 3.86 |
| 10580 | Albany-Schenectady-Troy, NY | 61 | 3.40 | 0.76 | 15.16 | 75 | 1.05 | 0.26 | 4.32 |
| 34980 | Nashville-Davidson--Murfreesboro--Franklin, TN | 365 | 2.34 | 0.55 | 10.01 | 58 | 1.07 | 0.26 | 4.37 |
| 33340 | Milwaukee-Waukesha-West Allis, WI | 6 | 4.73 | 1.14 | 19.62 | 79 | 1.05 | 0.26 | 4.25 |
| 33700 | Modesto, CA | 124 | 3.06 | 0.66 | 14.20 | 95 | 1.03 | 0.26 | 4.09 |
| 19100 | Dallas-Fort Worth-Arlington, TX | 380 | 1.84 | 0.51 | 6.58 | 321 | 0.99 | 0.28 | 3.44 |
| 48620 | Wichita, KS | 175 | 2.95 | 0.66 | 13.14 | 40 | 1.11 | 0.27 | 4.49 |
| 32820 | Memphis, TN-MS-AR | 381 | 1.81 | 0.45 | 7.28 | 32 | 1.14 | 0.28 | 4.61 |
| 37100 | Oxnard-Thousand Oaks-Ventura, CA | 98 | 3.20 | 0.68 | 14.95 | 372 | 0.93 | 0.23 | 3.69 |
| 47260 | Virginia Beach-Norfolk-Newport News, VA-NC | 25 | 3.87 | 0.94 | 15.91 | 360 | 0.96 | 0.23 | 3.92 |
| 38300 | Pittsburgh, PA | 73 | 3.34 | 0.79 | 14.11 | 308 | 0.99 | 0.24 | 4.09 |
| 17140 | Cincinnati, OH-KY-IN | 78 | 3.32 | 0.80 | 13.70 | 85 | 1.04 | 0.25 | 4.26 |
| 27980 | Kahului-Wailuku-Lahaina, HI | 52 | 3.49 | 0.74 | 16.57 | 276 | 1.01 | 0.24 | 4.15 |
| 26900 | Indianapolis-Carmel-Anderson, IN | 17 | 3.97 | 1.00 | 15.79 | 356 | 0.96 | 0.24 | 3.90 |
| 31080 | Los Angeles-Long Beach-Anaheim, CA | 28 | 3.82 | 1.12 | 13.09 | 4 | 1.37 | 0.43 | 4.39 |
| 19820 | Detroit-Warren-Dearborn, MI | 193 | 2.93 | 0.73 | 11.75 | 49 | 1.09 | 0.27 | 4.42 |
| 18140 | Columbus, OH | 5 | 4.83 | 1.22 | 19.07 | 222 | 1.02 | 0.25 | 4.15 |
| 46060 | Tucson, AZ | 29 | 3.82 | 0.86 | 16.87 | 3 | 1.38 | 0.36 | 5.27 |
| 41180 | St. Louis, MO-IL | 4 | 5.14 | 1.39 | 19.05 | 25 | 1.15 | 0.28 | 4.66 |
| 16980 | Chicago-Naperville-Elgin, IL-IN-WI | 1 | 6.51 | 1.84 | 23.09 | 10 | 1.24 | 0.34 | 4.61 |
| 27260 | Jacksonville, FL | 103 | 3.17 | 0.81 | 12.44 | 373 | 0.92 | 0.23 | 3.70 |
| 12580 | Baltimore-Columbia-Towson, MD | 30 | 3.80 | 1.00 | 14.47 | 357 | 0.96 | 0.24 | 3.89 |
| 14460 | Boston-Cambridge-Newton, MA-NH | 24 | 3.89 | 0.95 | 15.94 | 13 | 1.22 | 0.31 | 4.81 |
| 38900 | Portland-Vancouver-Hillsboro, OR-WA | 256 | 2.84 | 0.64 | 12.54 | 51 | 1.08 | 0.28 | 4.26 |
| 41860 | San Francisco-Oakland-Hayward, CA | 2 | 5.87 | 1.57 | 21.90 | 11 | 1.24 | 0.34 | 4.57 |
| 35620 | New York-Newark-Jersey City, NY-NJ-PA | 3 | 5.38 | 1.54 | 18.75 | 71 | 1.06 | 0.29 | 3.84 |
| 40900 | Sacramento--Roseville--Arden-Arcade, CA | 242 | 2.86 | 0.68 | 11.90 | 15 | 1.20 | 0.32 | 4.60 |
| 41940 | San Jose-Sunnyvale-Santa Clara, CA | 110 | 3.12 | 0.70 | 13.96 | 16 | 1.20 | 0.31 | 4.63 |
