# Supplementary material for: Mapping fatal police violence across U.S. metropolitan areas: Overall rates and racial/ethnic inequities, 2013-2017
Source: PLoS One. 2020 Jun 24;15(6):e0229686. doi: 10.1371/journal.pone.0229686 (PMC7313728; doi:10.1371/journal.pone.0229686)
Supplement: S5 Table — (DOCX) [file pone.0229686.s012.docx]

**S5 Table.** Comparing estimates of incident rates of fatalities involving police per 100,000 by MSA when using non-“accidental” vs. all-cause fatalities

| **MSA** | **MSA Name** | **Incident Rate (All-Cause)** | **Incident Rate (Non-"Accidental")** | **All-Cause /  Non-"Accidental" Ratio** | **Ratio Rank** |
| --- | --- | --- | --- | --- | --- |
| 10180 | Abilene, TX | 0.44 | 0.37 | 1.19 | 292 |
| 10420 | Akron, OH | 0.53 | 0.45 | 1.16 | 326 |
| 10500 | Albany, GA | 0.62 | 0.49 | 1.27 | 194 |
| 10540 | Albany, OR | 0.39 | 0.27 | 1.46 | 72 |
| 10580 | Albany-Schenectady-Troy, NY | 0.41 | 0.33 | 1.24 | 231 |
| 10740 | Albuquerque, NM | 1.28 | 0.92 | 1.38 | 114 |
| 10780 | Alexandria, LA | 0.51 | 0.43 | 1.17 | 316 |
| 10900 | Allentown-Bethlehem-Easton, PA-NJ | 0.46 | 0.40 | 1.16 | 324 |
| 11020 | Altoona, PA | 0.38 | 0.26 | 1.46 | 74 |
| 11100 | Amarillo, TX | 0.95 | 0.60 | 1.59 | 39 |
| 11180 | Ames, IA | 0.42 | 0.33 | 1.25 | 207 |
| 11260 | Anchorage, AK | 0.97 | 0.90 | 1.08 | 373 |
| 11460 | Ann Arbor, MI | 0.36 | 0.26 | 1.41 | 101 |
| 11500 | Anniston-Oxford-Jacksonville, AL | 1.23 | 1.17 | 1.05 | 382 |
| 11540 | Appleton, WI | 0.38 | 0.32 | 1.19 | 300 |
| 11700 | Asheville, NC | 0.46 | 0.36 | 1.28 | 184 |
| 12020 | Athens-Clarke County, GA | 0.49 | 0.34 | 1.45 | 76 |
| 12060 | Atlanta-Sandy Springs-Roswell, GA | 0.55 | 0.39 | 1.42 | 91 |
| 12100 | Atlantic City-Hammonton, NJ | 0.46 | 0.41 | 1.14 | 350 |
| 12220 | Auburn-Opelika, AL | 0.74 | 0.68 | 1.10 | 367 |
| 12260 | Augusta-Richmond County, GA-SC | 0.63 | 0.42 | 1.50 | 54 |
| 12420 | Austin-Round Rock, TX | 0.52 | 0.37 | 1.42 | 87 |
| 12540 | Bakersfield, CA | 1.13 | 1.01 | 1.12 | 360 |
| 12580 | Baltimore-Columbia-Towson, MD | 0.61 | 0.48 | 1.27 | 192 |
| 12620 | Bangor, ME | 0.75 | 0.62 | 1.22 | 248 |
| 12700 | Barnstable Town, MA | 0.44 | 0.22 | 1.96 | 8 |
| 12940 | Baton Rouge, LA | 0.82 | 0.64 | 1.29 | 164 |
| 12980 | Battle Creek, MI | 0.54 | 0.30 | 1.78 | 17 |
| 13020 | Bay City, MI | 0.52 | 0.38 | 1.38 | 117 |
| 13140 | Beaumont-Port Arthur, TX | 0.73 | 0.51 | 1.42 | 94 |
| 13220 | Beckley, WV | 0.62 | 0.48 | 1.30 | 157 |
| 13380 | Bellingham, WA | 0.39 | 0.33 | 1.19 | 297 |
| 13460 | Bend-Redmond, OR | 0.76 | 0.52 | 1.48 | 62 |
| 13740 | Billings, MT | 1.14 | 1.01 | 1.13 | 353 |
| 13780 | Binghamton, NY | 0.30 | 0.24 | 1.24 | 237 |
| 13820 | Birmingham-Hoover, AL | 0.67 | 0.44 | 1.52 | 49 |
| 13900 | Bismarck, ND | 0.61 | 0.53 | 1.14 | 337 |
| 13980 | Blacksburg-Christiansburg-Radford, VA | 0.69 | 0.62 | 1.10 | 366 |
| 14010 | Bloomington, IL | 0.42 | 0.35 | 1.19 | 295 |
| 14020 | Bloomington, IN | 0.39 | 0.32 | 1.21 | 265 |
| 14100 | Bloomsburg-Berwick, PA | 0.43 | 0.34 | 1.25 | 206 |
| 14260 | Boise City, ID | 0.50 | 0.42 | 1.18 | 309 |
| 14460 | Boston-Cambridge-Newton, MA-NH | 0.25 | 0.19 | 1.30 | 154 |
| 14500 | Boulder, CO | 0.50 | 0.41 | 1.21 | 267 |
| 14540 | Bowling Green, KY | 0.59 | 0.37 | 1.62 | 34 |
| 14740 | Bremerton-Silverdale, WA | 0.40 | 0.30 | 1.31 | 153 |
| 14860 | Bridgeport-Stamford-Norwalk, CT | 0.38 | 0.22 | 1.76 | 18 |
| 15180 | Brownsville-Harlingen, TX | 0.39 | 0.35 | 1.13 | 354 |
| 15260 | Brunswick, GA | 0.39 | 0.32 | 1.25 | 216 |
| 15380 | Buffalo-Cheektowaga-Niagara Falls, NY | 0.20 | 0.13 | 1.53 | 48 |
| 15500 | Burlington, NC | 0.45 | 0.25 | 1.82 | 16 |
| 15540 | Burlington-South Burlington, VT | 0.43 | 0.37 | 1.17 | 312 |
| 15680 | California-Lexington Park, MD | 0.35 | 0.27 | 1.28 | 176 |
| 15940 | Canton-Massillon, OH | 0.34 | 0.27 | 1.27 | 191 |
| 15980 | Cape Coral-Fort Myers, FL | 0.40 | 0.33 | 1.22 | 247 |
| 16020 | Cape Girardeau, MO-IL | 0.47 | 0.39 | 1.22 | 256 |
| 16060 | Carbondale-Marion, IL | 0.49 | 0.41 | 1.19 | 287 |
| 16180 | Carson City, NV | 0.56 | 0.46 | 1.22 | 246 |
| 16220 | Casper, WY | 0.82 | 0.48 | 1.72 | 25 |
| 16300 | Cedar Rapids, IA | 0.43 | 0.27 | 1.62 | 35 |
| 16540 | Chambersburg-Waynesboro, PA | 0.45 | 0.33 | 1.36 | 126 |
| 16580 | Champaign-Urbana, IL | 0.34 | 0.25 | 1.38 | 118 |
| 16620 | Charleston, WV | 0.83 | 0.61 | 1.36 | 124 |
| 16700 | Charleston-North Charleston, SC | 0.68 | 0.48 | 1.42 | 93 |
| 16740 | Charlotte-Concord-Gastonia, NC-SC | 0.47 | 0.34 | 1.38 | 115 |
| 16820 | Charlottesville, VA | 0.38 | 0.32 | 1.19 | 298 |
| 16860 | Chattanooga, TN-GA | 0.53 | 0.50 | 1.07 | 377 |
| 16940 | Cheyenne, WY | 0.61 | 0.45 | 1.34 | 131 |
| 16980 | Chicago-Naperville-Elgin, IL-IN-WI | 0.40 | 0.27 | 1.48 | 58 |
| 17020 | Chico, CA | 0.88 | 0.66 | 1.34 | 136 |
| 17140 | Cincinnati, OH-KY-IN | 0.44 | 0.31 | 1.41 | 102 |
| 17300 | Clarksville, TN-KY | 0.25 | 0.20 | 1.26 | 198 |
| 17420 | Cleveland, TN | 0.63 | 0.48 | 1.30 | 156 |
| 17460 | Cleveland-Elyria, OH | 0.45 | 0.32 | 1.42 | 89 |
| 17660 | Coeur d'Alene, ID | 0.41 | 0.34 | 1.21 | 262 |
| 17780 | College Station-Bryan, TX | 0.33 | 0.27 | 1.21 | 273 |
| 17820 | Colorado Springs, CO | 0.49 | 0.39 | 1.24 | 225 |
| 17860 | Columbia, MO | 0.53 | 0.46 | 1.15 | 335 |
| 17900 | Columbia, SC | 0.55 | 0.47 | 1.18 | 305 |
| 17980 | Columbus, GA-AL | 0.94 | 0.62 | 1.51 | 51 |
| 18020 | Columbus, IN | 0.44 | 0.35 | 1.25 | 205 |
| 18140 | Columbus, OH | 0.50 | 0.45 | 1.10 | 365 |
| 18580 | Corpus Christi, TX | 0.55 | 0.45 | 1.23 | 245 |
| 18700 | Corvallis, OR | 0.37 | 0.29 | 1.28 | 168 |
| 18880 | Crestview-Fort Walton Beach-Destin, FL | 0.52 | 0.42 | 1.23 | 239 |
| 19060 | Cumberland, MD-WV | 0.47 | 0.39 | 1.22 | 257 |
| 19100 | Dallas-Fort Worth-Arlington, TX | 0.54 | 0.43 | 1.25 | 208 |
| 19140 | Dalton, GA | 0.52 | 0.45 | 1.17 | 317 |
| 19180 | Danville, IL | 0.50 | 0.35 | 1.44 | 83 |
| 19300 | Daphne-Fairhope-Foley, AL | 0.70 | 0.53 | 1.32 | 146 |
| 19340 | Davenport-Moline-Rock Island, IA-IL | 0.41 | 0.31 | 1.35 | 129 |
| 19380 | Dayton, OH | 0.72 | 0.57 | 1.26 | 200 |
| 19460 | Decatur, AL | 0.57 | 0.29 | 1.95 | 9 |
| 19500 | Decatur, IL | 0.40 | 0.28 | 1.46 | 68 |
| 19660 | Deltona-Daytona Beach-Ormond Beach, FL | 0.92 | 0.65 | 1.41 | 104 |
| 19740 | Denver-Aurora-Lakewood, CO | 0.56 | 0.52 | 1.08 | 371 |
| 19780 | Des Moines-West Des Moines, IA | 0.65 | 0.31 | 2.10 | 4 |
| 19820 | Detroit-Warren-Dearborn, MI | 0.54 | 0.22 | 2.40 | 2 |
| 20020 | Dothan, AL | 0.57 | 0.39 | 1.48 | 60 |
| 20100 | Dover, DE | 0.64 | 0.58 | 1.11 | 361 |
| 20220 | Dubuque, IA | 0.54 | 0.33 | 1.62 | 32 |
| 20260 | Duluth, MN-WI | 0.38 | 0.33 | 1.17 | 310 |
| 20500 | Durham-Chapel Hill, NC | 0.53 | 0.34 | 1.56 | 45 |
| 20700 | East Stroudsburg, PA | 0.39 | 0.32 | 1.21 | 266 |
| 20740 | Eau Claire, WI | 0.72 | 0.59 | 1.22 | 250 |
| 20940 | El Centro, CA | 0.63 | 0.46 | 1.39 | 113 |
| 21060 | Elizabethtown-Fort Knox, KY | 0.57 | 0.44 | 1.30 | 161 |
| 21140 | Elkhart-Goshen, IN | 0.59 | 0.53 | 1.12 | 359 |
| 21300 | Elmira, NY | 0.49 | 0.40 | 1.22 | 255 |
| 21340 | El Paso, TX | 0.44 | 0.41 | 1.06 | 378 |
| 21420 | Enid, OK | 0.71 | 0.44 | 1.60 | 37 |
| 21500 | Erie, PA | 0.58 | 0.29 | 1.98 | 6 |
| 21660 | Eugene, OR | 0.43 | 0.31 | 1.35 | 128 |
| 21780 | Evansville, IN-KY | 0.43 | 0.25 | 1.74 | 21 |
| 21820 | Fairbanks, AK | 0.76 | 0.67 | 1.12 | 356 |
| 22020 | Fargo, ND-MN | 0.38 | 0.32 | 1.19 | 299 |
| 22140 | Farmington, NM | 1.25 | 1.01 | 1.24 | 226 |
| 22180 | Fayetteville, NC | 0.68 | 0.57 | 1.20 | 279 |
| 22220 | Fayetteville-Springdale-Rogers, AR-MO | 0.34 | 0.28 | 1.23 | 244 |
| 22380 | Flagstaff, AZ | 0.94 | 0.51 | 1.83 | 15 |
| 22420 | Flint, MI | 0.52 | 0.32 | 1.61 | 36 |
| 22500 | Florence, SC | 0.59 | 0.34 | 1.74 | 19 |
| 22520 | Florence-Muscle Shoals, AL | 0.77 | 0.70 | 1.10 | 368 |
| 22540 | Fond du Lac, WI | 0.41 | 0.33 | 1.25 | 212 |
| 22660 | Fort Collins, CO | 0.68 | 0.59 | 1.14 | 340 |
| 22900 | Fort Smith, AR-OK | 0.54 | 0.36 | 1.48 | 61 |
| 23060 | Fort Wayne, IN | 0.63 | 0.46 | 1.37 | 121 |
| 23420 | Fresno, CA | 0.70 | 0.57 | 1.22 | 251 |
| 23460 | Gadsden, AL | 0.75 | 0.51 | 1.46 | 70 |
| 23540 | Gainesville, FL | 0.46 | 0.40 | 1.14 | 349 |
| 23580 | Gainesville, GA | 0.33 | 0.27 | 1.24 | 232 |
| 23900 | Gettysburg, PA | 0.41 | 0.33 | 1.25 | 211 |
| 24020 | Glens Falls, NY | 0.34 | 0.26 | 1.28 | 182 |
| 24140 | Goldsboro, NC | 0.39 | 0.26 | 1.46 | 73 |
| 24220 | Grand Forks, ND-MN | 0.60 | 0.45 | 1.34 | 133 |
| 24260 | Grand Island, NE | 0.43 | 0.29 | 1.48 | 59 |
| 24300 | Grand Junction, CO | 0.63 | 0.56 | 1.13 | 352 |
| 24340 | Grand Rapids-Wyoming, MI | 0.42 | 0.29 | 1.45 | 77 |
| 24420 | Grants Pass, OR | 0.72 | 0.63 | 1.14 | 344 |
| 24500 | Great Falls, MT | 0.65 | 0.56 | 1.16 | 323 |
| 24540 | Greeley, CO | 0.61 | 0.52 | 1.18 | 307 |
| 24580 | Green Bay, WI | 0.39 | 0.31 | 1.28 | 183 |
| 24660 | Greensboro-High Point, NC | 0.48 | 0.22 | 2.18 | 3 |
| 24780 | Greenville, NC | 0.38 | 0.32 | 1.21 | 269 |
| 24860 | Greenville-Anderson-Mauldin, SC | 0.91 | 0.53 | 1.70 | 27 |
| 25060 | Gulfport-Biloxi-Pascagoula, MS | 0.72 | 0.50 | 1.45 | 79 |
| 25180 | Hagerstown-Martinsburg, MD-WV | 0.60 | 0.38 | 1.60 | 38 |
| 25220 | Hammond, LA | 0.61 | 0.36 | 1.71 | 26 |
| 25260 | Hanford-Corcoran, CA | 0.76 | 0.44 | 1.73 | 22 |
| 25420 | Harrisburg-Carlisle, PA | 0.49 | 0.41 | 1.21 | 275 |
| 25500 | Harrisonburg, VA | 0.38 | 0.30 | 1.24 | 219 |
| 25540 | Hartford-West Hartford-East Hartford, CT | 0.23 | 0.21 | 1.11 | 362 |
| 25620 | Hattiesburg, MS | 0.70 | 0.44 | 1.58 | 40 |
| 25860 | Hickory-Lenoir-Morganton, NC | 0.64 | 0.52 | 1.23 | 243 |
| 25940 | Hilton Head Island-Bluffton-Beaufort, SC | 0.36 | 0.30 | 1.21 | 271 |
| 25980 | Hinesville, GA | 0.44 | 0.35 | 1.25 | 204 |
| 26140 | Homosassa Springs, FL | 0.53 | 0.45 | 1.17 | 318 |
| 26300 | Hot Springs, AR | 0.94 | 0.45 | 2.08 | 5 |
| 26380 | Houma-Thibodaux, LA | 0.58 | 0.47 | 1.24 | 234 |
| 26420 | Houston-The Woodlands-Sugar Land, TX | 0.72 | 0.51 | 1.40 | 105 |
| 26580 | Huntington-Ashland, WV-KY-OH | 0.60 | 0.41 | 1.46 | 75 |
| 26620 | Huntsville, AL | 0.96 | 0.68 | 1.41 | 103 |
| 26820 | Idaho Falls, ID | 0.47 | 0.34 | 1.37 | 123 |
| 26900 | Indianapolis-Carmel-Anderson, IN | 0.59 | 0.42 | 1.39 | 112 |
| 26980 | Iowa City, IA | 0.44 | 0.37 | 1.19 | 291 |
| 27060 | Ithaca, NY | 0.36 | 0.28 | 1.28 | 174 |
| 27100 | Jackson, MI | 0.45 | 0.33 | 1.36 | 127 |
| 27140 | Jackson, MS | 0.67 | 0.48 | 1.40 | 107 |
| 27180 | Jackson, TN | 0.75 | 0.67 | 1.11 | 363 |
| 27260 | Jacksonville, FL | 0.73 | 0.64 | 1.14 | 347 |
| 27340 | Jacksonville, NC | 0.29 | 0.23 | 1.27 | 193 |
| 27500 | Janesville-Beloit, WI | 0.40 | 0.28 | 1.40 | 108 |
| 27620 | Jefferson City, MO | 0.41 | 0.34 | 1.21 | 263 |
| 27740 | Johnson City, TN | 0.54 | 0.43 | 1.26 | 197 |
| 27780 | Johnstown, PA | 0.59 | 0.52 | 1.15 | 336 |
| 27860 | Jonesboro, AR | 0.49 | 0.41 | 1.19 | 288 |
| 27900 | Joplin, MO | 0.58 | 0.41 | 1.42 | 88 |
| 27980 | Kahului-Wailuku-Lahaina, HI | 0.35 | 0.28 | 1.24 | 227 |
| 28020 | Kalamazoo-Portage, MI | 0.67 | 0.27 | 2.51 | 1 |
| 28100 | Kankakee, IL | 0.35 | 0.27 | 1.28 | 175 |
| 28140 | Kansas City, MO-KS | 0.88 | 0.64 | 1.38 | 116 |
| 28420 | Kennewick-Richland, WA | 0.58 | 0.48 | 1.20 | 280 |
| 28660 | Killeen-Temple, TX | 0.66 | 0.49 | 1.35 | 130 |
| 28700 | Kingsport-Bristol-Bristol, TN-VA | 0.67 | 0.54 | 1.25 | 215 |
| 28740 | Kingston, NY | 0.34 | 0.27 | 1.24 | 230 |
| 28940 | Knoxville, TN | 0.58 | 0.54 | 1.08 | 374 |
| 29020 | Kokomo, IN | 0.65 | 0.41 | 1.58 | 41 |
| 29100 | La Crosse-Onalaska, WI-MN | 0.37 | 0.30 | 1.24 | 220 |
| 29180 | Lafayette, LA | 0.70 | 0.60 | 1.16 | 327 |
| 29200 | Lafayette-West Lafayette, IN | 0.28 | 0.22 | 1.27 | 195 |
| 29340 | Lake Charles, LA | 0.40 | 0.34 | 1.19 | 296 |
| 29420 | Lake Havasu City-Kingman, AZ | 1.06 | 0.82 | 1.30 | 160 |
| 29460 | Lakeland-Winter Haven, FL | 0.40 | 0.32 | 1.24 | 238 |
| 29540 | Lancaster, PA | 0.41 | 0.32 | 1.27 | 188 |
| 29620 | Lansing-East Lansing, MI | 0.34 | 0.30 | 1.14 | 345 |
| 29700 | Laredo, TX | 0.55 | 0.45 | 1.21 | 261 |
| 29740 | Las Cruces, NM | 0.97 | 0.86 | 1.13 | 351 |
| 29820 | Las Vegas-Henderson-Paradise, NV | 0.65 | 0.59 | 1.09 | 369 |
| 29940 | Lawrence, KS | 0.34 | 0.27 | 1.28 | 178 |
| 30020 | Lawton, OK | 0.68 | 0.53 | 1.27 | 187 |
| 30140 | Lebanon, PA | 0.47 | 0.35 | 1.37 | 122 |
| 30300 | Lewiston, ID-WA | 0.40 | 0.31 | 1.29 | 165 |
| 30340 | Lewiston-Auburn, ME | 0.40 | 0.32 | 1.25 | 213 |
| 30460 | Lexington-Fayette, KY | 0.35 | 0.24 | 1.47 | 65 |
| 30620 | Lima, OH | 0.46 | 0.33 | 1.42 | 92 |
| 30700 | Lincoln, NE | 0.42 | 0.37 | 1.14 | 348 |
| 30780 | Little Rock-North Little Rock-Conway, AR | 0.80 | 0.66 | 1.21 | 278 |
| 30860 | Logan, UT-ID | 0.54 | 0.40 | 1.33 | 141 |
| 30980 | Longview, TX | 0.78 | 0.67 | 1.17 | 314 |
| 31020 | Longview, WA | 0.75 | 0.66 | 1.12 | 355 |
| 31080 | Los Angeles-Long Beach-Anaheim, CA | 0.60 | 0.49 | 1.22 | 259 |
| 31140 | Louisville/Jefferson County, KY-IN | 0.48 | 0.37 | 1.30 | 158 |
| 31180 | Lubbock, TX | 0.58 | 0.38 | 1.54 | 46 |
| 31340 | Lynchburg, VA | 0.56 | 0.46 | 1.21 | 260 |
| 31420 | Macon, GA | 0.42 | 0.32 | 1.31 | 150 |
| 31460 | Madera, CA | 0.51 | 0.43 | 1.17 | 315 |
| 31540 | Madison, WI | 0.40 | 0.35 | 1.16 | 328 |
| 31700 | Manchester-Nashua, NH | 0.29 | 0.24 | 1.19 | 303 |
| 31740 | Manhattan, KS | 0.36 | 0.28 | 1.28 | 172 |
| 31860 | Mankato-North Mankato, MN | 0.53 | 0.45 | 1.19 | 284 |
| 31900 | Mansfield, OH | 0.69 | 0.55 | 1.27 | 185 |
| 32580 | McAllen-Edinburg-Mission, TX | 0.57 | 0.30 | 1.88 | 10 |
| 32780 | Medford, OR | 0.53 | 0.42 | 1.26 | 199 |
| 32820 | Memphis, TN-MS-AR | 0.62 | 0.54 | 1.15 | 331 |
| 32900 | Merced, CA | 0.94 | 0.64 | 1.47 | 63 |
| 33100 | Miami-Fort Lauderdale-West Palm Beach, FL | 0.48 | 0.38 | 1.26 | 196 |
| 33140 | Michigan City-La Porte, IN | 0.40 | 0.27 | 1.46 | 69 |
| 33220 | Midland, MI | 0.38 | 0.29 | 1.28 | 167 |
| 33260 | Midland, TX | 0.72 | 0.59 | 1.22 | 249 |
| 33340 | Milwaukee-Waukesha-West Allis, WI | 0.45 | 0.33 | 1.34 | 134 |
| 33460 | Minneapolis-St. Paul-Bloomington, MN-WI | 0.36 | 0.26 | 1.40 | 109 |
| 33540 | Missoula, MT | 0.45 | 0.37 | 1.22 | 258 |
| 33660 | Mobile, AL | 0.58 | 0.44 | 1.32 | 147 |
| 33700 | Modesto, CA | 0.71 | 0.48 | 1.48 | 56 |
| 33740 | Monroe, LA | 0.69 | 0.57 | 1.22 | 252 |
| 33780 | Monroe, MI | 0.36 | 0.25 | 1.44 | 80 |
| 33860 | Montgomery, AL | 0.52 | 0.31 | 1.67 | 30 |
| 34060 | Morgantown, WV | 0.59 | 0.40 | 1.48 | 57 |
| 34100 | Morristown, TN | 0.57 | 0.43 | 1.34 | 137 |
| 34580 | Mount Vernon-Anacortes, WA | 0.34 | 0.27 | 1.28 | 179 |
| 34620 | Muncie, IN | 0.57 | 0.49 | 1.17 | 322 |
| 34740 | Muskegon, MI | 0.34 | 0.24 | 1.44 | 82 |
| 34820 | Myrtle Beach-Conway-North Myrtle Beach, SC-NC | 0.60 | 0.46 | 1.30 | 163 |
| 34900 | Napa, CA | 0.53 | 0.45 | 1.17 | 319 |
| 34940 | Naples-Immokalee-Marco Island, FL | 0.37 | 0.26 | 1.41 | 100 |
| 34980 | Nashville-Davidson--Murfreesboro--Franklin, TN | 0.33 | 0.27 | 1.23 | 241 |
| 35100 | New Bern, NC | 0.49 | 0.36 | 1.37 | 120 |
| 35300 | New Haven-Milford, CT | 0.38 | 0.22 | 1.74 | 20 |
| 35380 | New Orleans-Metairie, LA | 0.73 | 0.53 | 1.38 | 119 |
| 35620 | New York-Newark-Jersey City, NY-NJ-PA | 0.20 | 0.15 | 1.34 | 135 |
| 35660 | Niles-Benton Harbor, MI | 0.68 | 0.43 | 1.58 | 43 |
| 35840 | North Port-Sarasota-Bradenton, FL | 0.49 | 0.42 | 1.16 | 325 |
| 35980 | Norwich-New London, CT | 0.55 | 0.41 | 1.34 | 138 |
| 36100 | Ocala, FL | 0.51 | 0.36 | 1.43 | 85 |
| 36140 | Ocean City, NJ | 0.36 | 0.28 | 1.28 | 169 |
| 36220 | Odessa, TX | 0.68 | 0.55 | 1.24 | 224 |
| 36260 | Ogden-Clearfield, UT | 0.52 | 0.42 | 1.24 | 221 |
| 36420 | Oklahoma City, OK | 1.10 | 0.93 | 1.19 | 285 |
| 36500 | Olympia-Tumwater, WA | 0.43 | 0.37 | 1.15 | 332 |
| 36540 | Omaha-Council Bluffs, NE-IA | 0.78 | 0.59 | 1.32 | 144 |
| 36740 | Orlando-Kissimmee-Sanford, FL | 0.44 | 0.31 | 1.40 | 110 |
| 36780 | Oshkosh-Neenah, WI | 0.44 | 0.37 | 1.19 | 293 |
| 36980 | Owensboro, KY | 0.57 | 0.49 | 1.17 | 321 |
| 37100 | Oxnard-Thousand Oaks-Ventura, CA | 0.35 | 0.30 | 1.15 | 333 |
| 37340 | Palm Bay-Melbourne-Titusville, FL | 0.59 | 0.51 | 1.17 | 311 |
| 37460 | Panama City, FL | 0.66 | 0.44 | 1.51 | 53 |
| 37620 | Parkersburg-Vienna, WV | 0.70 | 0.53 | 1.31 | 152 |
| 37860 | Pensacola-Ferry Pass-Brent, FL | 0.50 | 0.46 | 1.08 | 370 |
| 37900 | Peoria, IL | 0.33 | 0.25 | 1.30 | 159 |
| 37980 | Philadelphia-Camden-Wilmington, PA-NJ-DE-MD | 0.33 | 0.25 | 1.33 | 139 |
| 38060 | Phoenix-Mesa-Scottsdale, AZ | 0.80 | 0.73 | 1.11 | 364 |
| 38220 | Pine Bluff, AR | 0.55 | 0.46 | 1.19 | 283 |
| 38300 | Pittsburgh, PA | 0.45 | 0.30 | 1.47 | 66 |
| 38340 | Pittsfield, MA | 0.38 | 0.31 | 1.24 | 218 |
| 38540 | Pocatello, ID | 0.50 | 0.41 | 1.22 | 254 |
| 38860 | Portland-South Portland, ME | 0.36 | 0.32 | 1.12 | 358 |
| 38900 | Portland-Vancouver-Hillsboro, OR-WA | 0.39 | 0.37 | 1.05 | 381 |
| 38940 | Port St. Lucie, FL | 0.51 | 0.41 | 1.24 | 229 |
| 39140 | Prescott, AZ | 0.77 | 0.72 | 1.08 | 376 |
| 39300 | Providence-Warwick, RI-MA | 0.21 | 0.17 | 1.28 | 171 |
| 39340 | Provo-Orem, UT | 0.48 | 0.42 | 1.14 | 346 |
| 39380 | Pueblo, CO | 1.00 | 0.95 | 1.06 | 379 |
| 39460 | Punta Gorda, FL | 0.43 | 0.36 | 1.19 | 294 |
| 39540 | Racine, WI | 0.33 | 0.27 | 1.24 | 233 |
| 39580 | Raleigh, NC | 0.33 | 0.21 | 1.56 | 44 |
| 39660 | Rapid City, SD | 0.85 | 0.64 | 1.33 | 140 |
| 39740 | Reading, PA | 0.45 | 0.32 | 1.41 | 99 |
| 39820 | Redding, CA | 1.09 | 0.82 | 1.32 | 145 |
| 39900 | Reno, NV | 0.88 | 0.82 | 1.08 | 372 |
| 40060 | Richmond, VA | 0.55 | 0.36 | 1.53 | 47 |
| 40140 | Riverside-San Bernardino-Ontario, CA | 0.65 | 0.55 | 1.18 | 306 |
| 40220 | Roanoke, VA | 0.46 | 0.38 | 1.23 | 240 |
| 40340 | Rochester, MN | 0.32 | 0.26 | 1.24 | 235 |
| 40380 | Rochester, NY | 0.21 | 0.17 | 1.22 | 253 |
| 40420 | Rockford, IL | 0.55 | 0.29 | 1.86 | 13 |
| 40580 | Rocky Mount, NC | 0.51 | 0.39 | 1.33 | 142 |
| 40660 | Rome, GA | 0.48 | 0.33 | 1.43 | 86 |
| 40900 | Sacramento--Roseville--Arden-Arcade, CA | 0.56 | 0.44 | 1.28 | 173 |
| 40980 | Saginaw, MI | 0.46 | 0.27 | 1.72 | 24 |
| 41060 | St. Cloud, MN | 0.37 | 0.30 | 1.21 | 270 |
| 41100 | St. George, UT | 0.40 | 0.33 | 1.21 | 264 |
| 41140 | St. Joseph, MO-KS | 0.61 | 0.54 | 1.14 | 338 |
| 41180 | St. Louis, MO-IL | 0.75 | 0.60 | 1.25 | 209 |
| 41420 | Salem, OR | 0.46 | 0.38 | 1.20 | 282 |
| 41500 | Salinas, CA | 0.66 | 0.59 | 1.12 | 357 |
| 41540 | Salisbury, MD-DE | 0.57 | 0.33 | 1.72 | 23 |
| 41620 | Salt Lake City, UT | 0.49 | 0.40 | 1.23 | 242 |
| 41660 | San Angelo, TX | 0.63 | 0.48 | 1.30 | 155 |
| 41700 | San Antonio-New Braunfels, TX | 0.82 | 0.56 | 1.47 | 67 |
| 41740 | San Diego-Carlsbad, CA | 0.46 | 0.35 | 1.31 | 151 |
| 41860 | San Francisco-Oakland-Hayward, CA | 0.55 | 0.39 | 1.40 | 106 |
| 41940 | San Jose-Sunnyvale-Santa Clara, CA | 0.42 | 0.34 | 1.25 | 203 |
| 42020 | San Luis Obispo-Paso Robles-Arroyo Grande, CA | 0.31 | 0.26 | 1.21 | 276 |
| 42100 | Santa Cruz-Watsonville, CA | 0.46 | 0.37 | 1.26 | 201 |
| 42140 | Santa Fe, NM | 0.91 | 0.85 | 1.08 | 375 |
| 42200 | Santa Maria-Santa Barbara, CA | 0.52 | 0.42 | 1.24 | 228 |
| 42220 | Santa Rosa, CA | 0.59 | 0.42 | 1.43 | 84 |
| 42340 | Savannah, GA | 0.77 | 0.54 | 1.42 | 90 |
| 42540 | Scranton--Wilkes-Barre--Hazleton, PA | 0.30 | 0.27 | 1.14 | 343 |
| 42660 | Seattle-Tacoma-Bellevue, WA | 0.54 | 0.41 | 1.31 | 148 |
| 42680 | Sebastian-Vero Beach, FL | 0.46 | 0.34 | 1.36 | 125 |
| 42700 | Sebring, FL | 0.41 | 0.33 | 1.25 | 210 |
| 43100 | Sheboygan, WI | 0.45 | 0.32 | 1.42 | 95 |
| 43300 | Sherman-Denison, TX | 0.49 | 0.26 | 1.86 | 14 |
| 43340 | Shreveport-Bossier City, LA | 0.78 | 0.42 | 1.86 | 12 |
| 43420 | Sierra Vista-Douglas, AZ | 0.61 | 0.54 | 1.14 | 339 |
| 43580 | Sioux City, IA-NE-SD | 0.59 | 0.37 | 1.62 | 33 |
| 43620 | Sioux Falls, SD | 0.33 | 0.27 | 1.21 | 274 |
| 43780 | South Bend-Mishawaka, IN-MI | 0.57 | 0.31 | 1.87 | 11 |
| 43900 | Spartanburg, SC | 0.53 | 0.44 | 1.20 | 281 |
| 44060 | Spokane-Spokane Valley, WA | 0.70 | 0.61 | 1.14 | 342 |
| 44100 | Springfield, IL | 0.48 | 0.29 | 1.65 | 31 |
| 44140 | Springfield, MA | 0.34 | 0.23 | 1.51 | 50 |
| 44180 | Springfield, MO | 0.70 | 0.60 | 1.17 | 313 |
| 44220 | Springfield, OH | 0.60 | 0.30 | 1.98 | 7 |
| 44300 | State College, PA | 0.31 | 0.25 | 1.27 | 186 |
| 44420 | Staunton-Waynesboro, VA | 0.39 | 0.31 | 1.25 | 217 |
| 44700 | Stockton-Lodi, CA | 0.93 | 0.55 | 1.67 | 29 |
| 44940 | Sumter, SC | 0.40 | 0.32 | 1.25 | 214 |
| 45060 | Syracuse, NY | 0.33 | 0.22 | 1.51 | 52 |
| 45220 | Tallahassee, FL | 0.58 | 0.47 | 1.24 | 223 |
| 45300 | Tampa-St. Petersburg-Clearwater, FL | 0.51 | 0.42 | 1.21 | 272 |
| 45460 | Terre Haute, IN | 0.39 | 0.32 | 1.21 | 268 |
| 45500 | Texarkana, TX-AR | 0.51 | 0.39 | 1.33 | 143 |
| 45540 | The Villages, FL | 0.45 | 0.32 | 1.42 | 97 |
| 45780 | Toledo, OH | 0.33 | 0.27 | 1.21 | 277 |
| 45820 | Topeka, KS | 0.91 | 0.70 | 1.31 | 149 |
| 45940 | Trenton, NJ | 0.52 | 0.44 | 1.18 | 308 |
| 46060 | Tucson, AZ | 0.91 | 0.71 | 1.27 | 189 |
| 46140 | Tulsa, OK | 1.04 | 0.88 | 1.18 | 304 |
| 46220 | Tuscaloosa, AL | 0.50 | 0.31 | 1.58 | 42 |
| 46340 | Tyler, TX | 0.31 | 0.25 | 1.24 | 236 |
| 46520 | Urban Honolulu, HI | 0.32 | 0.25 | 1.28 | 180 |
| 46540 | Utica-Rome, NY | 0.34 | 0.28 | 1.19 | 302 |
| 46660 | Valdosta, GA | 0.47 | 0.39 | 1.19 | 290 |
| 46700 | Vallejo-Fairfield, CA | 0.66 | 0.56 | 1.19 | 301 |
| 47020 | Victoria, TX | 0.60 | 0.45 | 1.34 | 132 |
| 47220 | Vineland-Bridgeton, NJ | 0.82 | 0.55 | 1.49 | 55 |
| 47260 | Virginia Beach-Norfolk-Newport News, VA-NC | 0.44 | 0.34 | 1.28 | 170 |
| 47300 | Visalia-Porterville, CA | 0.83 | 0.57 | 1.47 | 64 |
| 47380 | Waco, TX | 0.89 | 0.85 | 1.05 | 380 |
| 47460 | Walla Walla, WA | 0.40 | 0.31 | 1.29 | 166 |
| 47580 | Warner Robins, GA | 0.51 | 0.35 | 1.46 | 71 |
| 47900 | Washington-Arlington-Alexandria, DC-VA-MD-WV | 0.35 | 0.25 | 1.41 | 98 |
| 47940 | Waterloo-Cedar Falls, IA | 0.39 | 0.28 | 1.40 | 111 |
| 48060 | Watertown-Fort Drum, NY | 0.34 | 0.27 | 1.28 | 177 |
| 48140 | Wausau, WI | 0.53 | 0.46 | 1.17 | 320 |
| 48260 | Weirton-Steubenville, WV-OH | 0.50 | 0.42 | 1.19 | 286 |
| 48300 | Wenatchee, WA | 0.64 | 0.56 | 1.14 | 341 |
| 48540 | Wheeling, WV-OH | 0.37 | 0.30 | 1.24 | 222 |
| 48620 | Wichita, KS | 0.95 | 0.56 | 1.69 | 28 |
| 48660 | Wichita Falls, TX | 0.57 | 0.44 | 1.30 | 162 |
| 48700 | Williamsport, PA | 0.45 | 0.32 | 1.42 | 96 |
| 48900 | Wilmington, NC | 0.46 | 0.37 | 1.26 | 202 |
| 49020 | Winchester, VA-WV | 0.48 | 0.40 | 1.19 | 289 |
| 49180 | Winston-Salem, NC | 0.29 | 0.20 | 1.45 | 78 |
| 49340 | Worcester, MA-CT | 0.26 | 0.21 | 1.28 | 181 |
| 49420 | Yakima, WA | 0.77 | 0.67 | 1.15 | 329 |
| 49620 | York-Hanover, PA | 0.65 | 0.45 | 1.44 | 81 |
| 49660 | Youngstown-Warren-Boardman, OH-PA | 0.40 | 0.32 | 1.27 | 190 |
| 49700 | Yuba City, CA | 0.98 | 0.85 | 1.15 | 330 |
| 49740 | Yuma, AZ | 0.49 | 0.43 | 1.15 | 334 |
